# Microwave‐Driven Dielectric‐Magnetic Regulation of Graphite@α‐MnO_2_ Toward Enhanced Electromagnetic Wave Absorption

**DOI:** 10.1002/advs.202504489

**Published:** 2025-06-20

**Authors:** Junyu Lu, Lei Xu, Cheng Xie, Chang Zhang, Zhaohui Han, Yiyao Ren, Renchao Che

**Affiliations:** ^1^ Faculty of Metallurgical and Energy Engineering Kunming University of Science and Technology Kunming 650093 PR China; ^2^ National Local Joint Laboratory of Engineering Application of Microwave Energy and Equipment Technology Kunming 650093 PR China; ^3^ The Key Laboratory of Unconventional Metallurgy Ministry of Education Kunming 650093 PR China; ^4^ Laboratory of Advanced Materials Shanghai Key Lab of Molecular Catalysis and Innovative Materials Academy for Engineering and Technology Advanced Coatings Research Center of Ministry of Education of China Fudan University Shanghai 220438 PR China

**Keywords:** electromagnetic wave absorption, functional structure, impedance matching, microwave activation, microwave hydrothermal, nanowire

## Abstract

The advancement of wireless communication technologies necessitates materials that absorb electromagnetic waves and shield against electromagnetic interference. The research is propelled by the necessity to develop materials that possess both properties. Here, an electromagnetic wave‐absorbing material that synergistically regulates dielectric and magnetic properties is developed. The material features a nanowire fabric/multilayer composite structure of expanded graphite@α‐MnO_2_ (EG@MO). The manganese oxide nanowires are prepared in situ on the surface of multilayer expanded graphite via microwave‐assisted hydrothermal synthesis. A rapid microwave activation process is subsequently performed to convert manganese oxide‐hydroxide, elevating its oxidation state, and transforming it from a non‐magnetic form to the magnetic α‐MnO_2_. The EG@MO exhibits exceptional electromagnetic wave absorption capabilities, achieving a reflection loss value of −75.56 dB, with a low filler ratio of 7 wt.% and an ultrathin thickness of 1.48 mm. This high‐performance electromagnetic absorption material, fabricated by integrating magnetic manganese dioxide nanowires with multilayer expanded graphite, shows excellent widespread applications.

## Graphical abstract

This study presents a microwave‐activated composite integrating expanded graphite with α‐MnO_2_ nanowires, engineered to synergistically optimize dielectric and magnetic properties. The innovative microwave‐driven process enables rapid structural transformation, yielding ultrathin, scalable electromagnetic wave‐absorbing films. The material demonstrates dual functionality in absorption and shielding, offering a promising probability for high‐performance electromagnetic management.

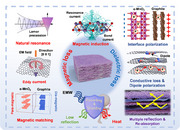

## 1 Introduction

The evolution of wireless communication technology has led to a significant increase in the utilization of electromagnetic waves (EMW), such as radio waves, microwaves, and infrared radiation, for the transmission of information among diverse devices and systems. This advancement has profoundly influenced numerous sectors, including mobile telecommunications, Wi‐Fi networks, satellite communications, and medical imaging. The diffusion of electromagnetic waves in space not only impedes the functionality of precision electronic apparatus^[^ ^1^ ^]^ but also presents a risk to human safety.^[^ ^2^ ^]^ Consequently, high‐performance electromagnetic wave absorption and electromagnetic interference (EMI) shielding materials are a crucial requirement. Their demand has increasingly become a central area of investigation across various sectors, such as electronic information, aerospace, precision instrumentation, military applications, and health.

To maximize the EMW absorption performance, it is essential to meticulously design the microstructure of the material.^[^ ^3^, ^4^, ^5^ ^]^ This involves regulating its internal structure at a microscopic scale and accurately adjusting its electromagnetic characteristics, dielectric, and magnetic properties.^[^ ^6^, ^7^ ^]^ One strategy is to adjust dielectric properties by combining conductors, such as graphene,^[^ ^8^, ^9^ ^]^ MXene,^[^ ^10^, ^11^, ^12^ ^]^ and carbon nanotubes,^[^ ^13^ ^]^ with semiconductors, including transition metal sulfides and oxides. The other strategy focuses on optimizing magnetic loss through the exploitation of magnetic material properties,^[^ ^14^ ^]^ such as resonance^[^ ^15^ ^]^ and space matching.^[^ ^16^ ^]^ The regulation of impedance matching by magnetic properties, in particular the impact of paramagnetic‐diamagnetic transitions on electromagnetic wave absorption performance, has not, however, been thoroughly studied.

The complex electronic and crystal structure of MnO₂^[^ ^17^, ^18^ ^]^ gives rise to the diverse magnetic and dielectric properties observed in its derivatives. Among these, α‐MnO₂ forms a 1D nanowire‐like structure comprising [MnO_6_] octahedral double chains.^[^ ^19^ ^]^ Expanded graphite (EG) is a lightweight and porous substance characterized by a honeycomb configuration. In essence, EG is a modified variant of graphite, distinguished by its markedly increased interlayer spacing resulting from the expansion process.^[^ ^20^, ^21^, ^22^, ^23^ ^]^ Previous studies^[^ ^24^, ^25^ ^]^ have demonstrated the efficacy of EG in electromagnetic wave shielding. More importantly, graphite is expected to enhance EMW absorption and EMI performance in electromagnetic waves due to its diamagnetic property by electromagnetic induction, which matches the paramagnetic property of MnO_2_.

However, the active valence transition between manganese oxides and hydroxides makes the hydrolysis of MnO2 during synthesis unavoidably produce the by‐product MnO(OH). Although both are similar in composition, the spin property of Mn in MnO(OH) makes it virtually nonmagnetic. Therefore, the modulation of dielectric and magnetic properties can be realized by controlling their valence and structure. Conventional heat treatment methods require long roasting times, whereas thermal activation can be achieved in a very short time using microwave heat treatment.

This work presents an approach to develop multilayer fabric EG@MO microwave absorbers that involves the in situ growth of α‐MnO₂ nanowires on the surface of layered EG by microwave hydrothermal to form a fabric‐encapsulated structure. Followed by a microwave activation for the transformation of manganese oxides from biphasic to monophasic, from low‐valent to high‐valent, and from nonmagnetic to magnetic. The synergistic effect between nanowires and layered structures in the design of functional structures^[^ ^26^, ^27^, ^28^, ^29^ ^]^ provides a multitude of avenues for electromagnetic wave absorption in addition to facilitating improvements in impedance matching.^[^ ^30^, ^31^ ^]^ The synthesized EG@MO composites exhibit the ability to effectively absorb electromagnetic waves at minimal thickness and low filling ratios. This work introduces a novel approach for the fabrication of materials that demonstrate high efficiency in the absorption of electromagnetic waves.

## 2 Results and Discussion

### 2.1 Microwave Activation of EG@MO

**Figure 1:**
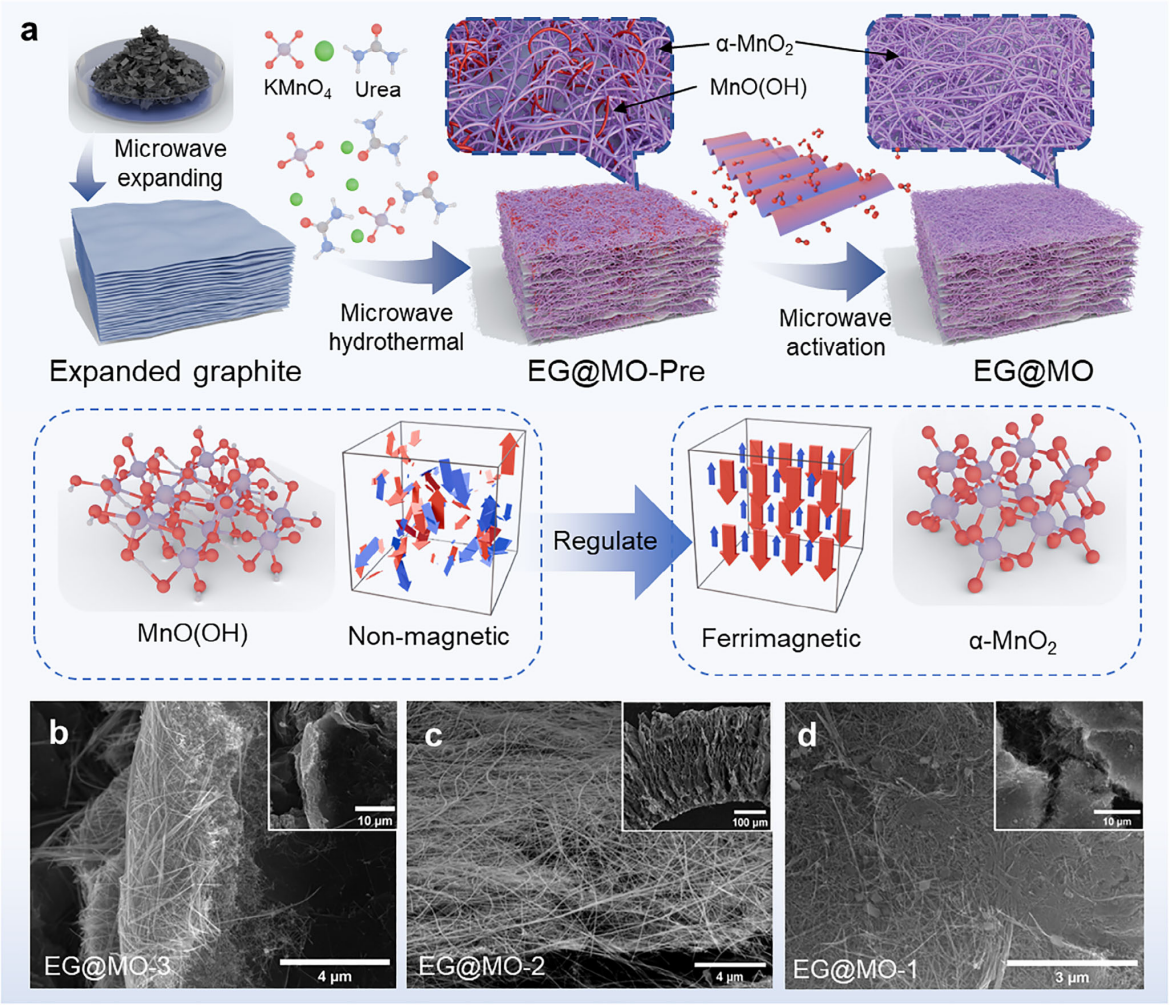
a)Schematic illustration of the synthesis and processing of EG@MO, b–d) SEM images of EG@MO‐1 to 3.

The synthesis of EG@MO was conducted following the method illustrated in **Figure** **1**a, in which KMnO_4_ was reduced to manganese oxides by reacting with urea at 180 °C under microwave hydrothermal conditions for 120 mins. The synthesis process is discussed in detail in the Supporting Information, and the synthesized precursor EG@MO‐Pre contains two manganese oxides, α‐MnO_2_ and MnO(OH). Since MnO(OH) is a non‐magnetic material, its magnetic properties are strengthened when it undergoes a phase homogenization transformation through fast activation with microwave irradiation to become α‐MnO_2_. The synthesized EG@MO shows a uniform EG@MO nanowire/layer structure as shown in Figure 1b–d. KMnO_4_ partially oxidized the edges of EG, leading to the initiation of nanowire growth at these oxidized regions (Figure 1b; Figure S2, Supporting Information). With the increase of Mn content, the nanowires were gradually and uniformly encapsulated on EG to form the EG@MO structure (Figure 1c). XRD analysis showed the same result (Figure S3, Supporting Information). Further, the excess MnO(OH) had precipitated with ammonium formate ions, which are derived from the hydrolysis of urea, leading to the formation of the Mn_3_O_4_ phase (refer to Equations S1–S4, Supporting Information), as illustrated in Figure 1c and Figure S4 (Supporting Information). To achieve superior performance in electromagnetic wave absorption, this study focused on regulating the magnetic and dielectric characteristics of EG@MO composites. This activation was accomplished through the phase homogenization of manganese oxides, utilizing valence transition, electronic structure transition, and magnetic structure transition to ensure effective impedance matching. In addition, the activation of EG@MO by the conventional method requires more than 1 h (40 min of heating and 20 min of roasting), but the microwave activation can reduce the processing time to 10s, which significantly shortens the processing time and improves the preparation efficiency. The microwave activation is discussed in detail in the Supporting Information, and Video S1 (Supporting Information) is provided to show the significant changes in their electromagnetic wave absorption capacity before and after activation.

**Figure 2:**
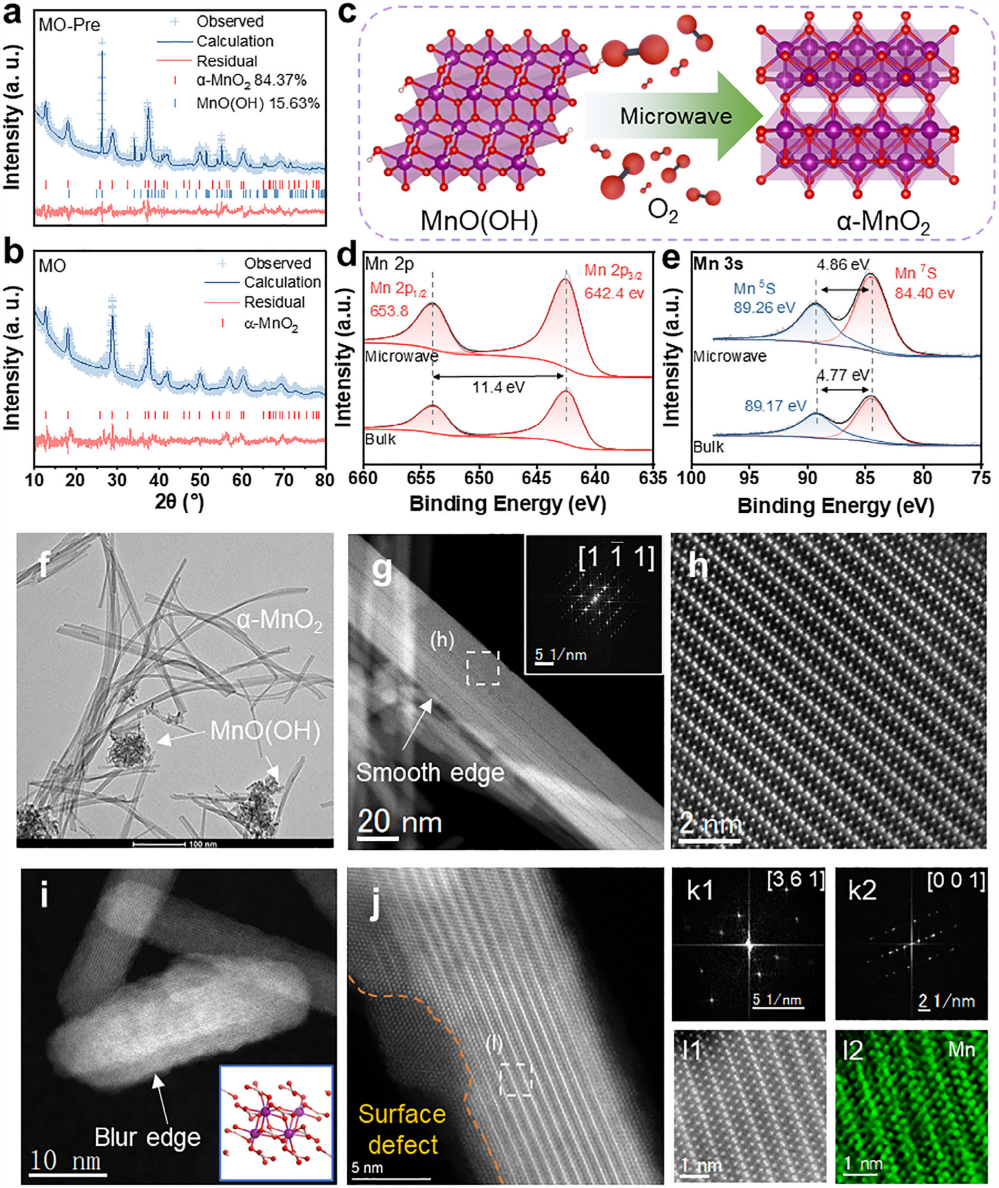
XRD Rietveld refinement of a) MO‐Pre and b) MO; c) Schematic illustration of the transformation of MnO(OH) to α‐MnO_2_; XPS spectra of d) Mn 2p and e) Mn 3s; f) TEM images of MO‐Pre; g,h) HAADF‐STEM image and FFT patterns in [1‐1 1] direction of the α‐MnO_2_ nanowire in MO‐Pre; HAADF‐STEM image of the MnO(OH) nanorods in i) MO‐Pre and j) MO after treatment; k1 and k2) FFT patterns corresponding to (i) and (j); l1 and l2) EDS mapping corresponding to (j).

To understand the microwave activation process of the precursor EG@MO‐Pre into EG@MO through phase homogenization, an analysis was conducted on the alterations in both the crystal structure and electronic structure of MO. The XRD Rietveld refinement spectrum of the microwave hydrothermal precursor MO‐Pre is shown in **Figure** **2**a, and the crystal structure of MO‐Pre, which mainly consisted of 84.37 wt. % α‐MnO_2_ (ICSD‐20227) and 15.63 wt. % MnO(OH) (ICSD‐00296). After the microwave activation (Figure 2b), the characteristic peaks of MnO(OH) in the sample disappeared, and no other new peaks were generated, proving that MnO(OH) had been completely converted to α‐MnO_2_ at this point. The XPS of MO before and after the microwave treatment is shown in Figure 2d,e. The results in the Mn_2p_ orbitals were almost the same, with a pair of 2p_3/2_ and 2p_1/2_ spin‐electron cleavage peaks appearing at 642.4 and 653.8 eV, respectively, with a spacing of 11.4 eV. In the Mn_3s_ orbital, the ^7^S peak appeared at 84.40 eV for both pre‐ and post‐treated samples, while the 5S peaks appeared at 89.26 and 89.17 eV, respectively. The binding energy of Mn in the treated samples increased slightly by 0.09 eV, representing the increase in the Mn valence state.

Figure 2f shows the TEM images of MO‐Pre, revealing a microscopic morphology characterized by elongated nanowires and clusters of short nanorods. The high‐angle annular dark‐field (HAADF) images of the nanowires are shown in Figure 2g,h. The Fourier transform pattern indicates that the elongated nanowires were composed of α‐MnO_2_, exhibiting an atomic phase arrangement along the [1‐1 1] crystallographic band axis. The MnO_2_ crystals were generated in the c‐direction to produce a perfectly smooth hollow nanowire. These nanowires were observed to have smooth and flat surfaces and fewer defects from their linear degree, particularly α‐MnO_2,_ a tetragonal crystal system that forms a double‐chain structure with manganese oxide octahedra [MnO_6_] connected along the c‐axis in a co‐rimmed manner. These double chains of [MnO_6_] share a top angle with neighboring double chains, thus forming 1D (1 × 1) and (2 × 2) tunneling structures. This 1D nanowire structure provides excellent magnetic properties for α‐MnO_2_, while the tunneling structure provides a special conduction path for electromagnetic waves. The HAADF image of the nanorod is presented in Figure 2i, exhibiting rough and blurred edges. This structure can be identified as MnO(OH) based on the fast Fourier transform (FFT) pattern depicted in Figure 2k1. The crystal structure of the nanorod after microwave oxidation roasting (Figure 2j) was transformed into α‐MnO_2_ as evidenced by the FFT pattern presented in Figure 2k2. However, the structure continued to exhibit blurred and rough edges and produce surface defects, which are observable through the unevenness in contrast. This is due to the removal of bound water and surface hydroxyl groups during high‐temperature roasting. The presence of these surface defects is conducive to the polarization of electromagnetic waves. Additionally, the conversion of non‐magnetic MnO(OH) to magnetic α‐MnO_2_ not only enhanced the magnetic properties of the composites but also improved the loss and absorption of electromagnetic waves.

**Figure 3:**
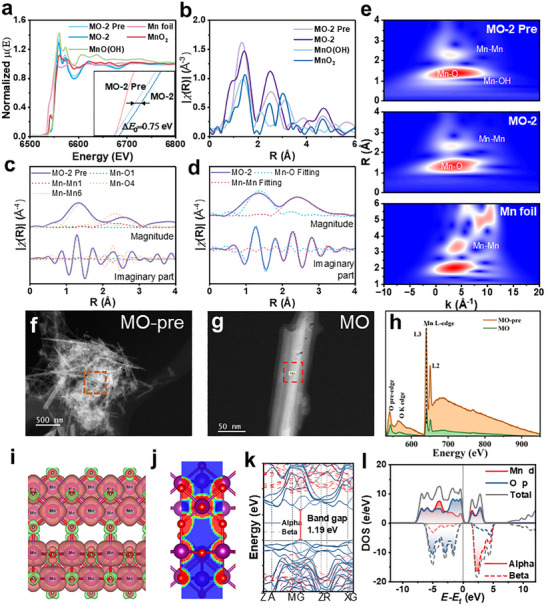
XAFS spectra of the Mn K‐edge. a) normalized XANES spectrum, b) k2 weighted Fourier transform‐EXAFS spectrum, FEFFs paths fitting of FT‐EXAFS spectra in c) MO‐2 Pre and d) MO‐2, e) k1 weighted wavelet transform EXAFS spectrum; HAADF‐STEM image of f) MO‐Pre and g) MO; h) EELS characterization; i) electron spin density, j) electron localization function, k) band structure, l) projected density of state of α‐MnO_2._

It is worth noting that although Mn in MnO(OH) has a Mn^III^ valence, which is lower than the Mn^IV^ valence in α‐MnO_2_, the two are difficult to distinguish in XPS. This occurs due to the presence of the more polar hydroxyl group in MnO(OH), which leads to the delocalization of electrons surrounding the Mn atoms. This changes the surface electron distribution and interferes with the acquisition of accurate information by XPS. Therefore, the samples were analyzed by X‐ray absorption fine structure spectroscopy (XAFS), and the X‐ray absorption near‐edge structure (XANES) spectrum of MO is shown in **Figure** **3**a. The valence state of MO was found to be intermediate between the Mn^III^ valence of MnO(OH) and the Mn^IV^ valence of α‐MnO_2_. Following microwave treatment, its binding energy E_0_ increased by 0.75 eV, indicating a transition in its valence state. In the k^2^‐weighted R‐space of the extended edge (EXAFS, Figure 3b), MO‐Pre corresponds to the MnO(OH) standard sample at 1.34 Å and to the peak of the MnO_2_ standard sample at 2.52 Å. The peaks of MO near 1.47 and 2.52 Å correspond to the MnO_2_ standard sample, illustrating the complete conversion of MO into MnO_2_ after microwave‐activating treatment. To further understand its coordination information, a path‐fitting analysis of MO‐Pre was performed (Figure 3c). In the first shell where R< 2Å, both Mn‐O1 coordination provided by MnO(OH) and Mn‐O4 coordination provided by MnO_2_ appeared. Similarly, Mn‐Mn1 coordination provided by MnO(OH) and Mn‐Mn6 coordination provided by MnO_2_ appeared in the second shell of 2Å< R< 3Å. In contrast, the path analysis of MO after microwave treatment (Figure 3d) showed that MO consisted only of Mn‐O4 coordination provided by MnO_2_ and Mn‐Mn6 coordination. Further analysis of MO‐Pre and MO using k1‐weighted wavelet transformation (Figure 3e) reveals that the Mn‐OH partially disappears near a k of 7 Å^−1^, at which point the MnO(OH) in MO‐Pre has been converted to α‐MnO_2_. It can be inferred from XRD refinement and EXAFS analysis that MO was made up entirely of α‐MnO_2_ and that its electronic structure is the key to its magnetic properties.

To further investigate the evolution of the electronic structure, atomic‐resolution electron energy loss spectroscopy (EELS) line scanning was performed on MO‐pre and MO samples. Figure 3f to h presents the HAADF‐STEM images with the corresponding EELS line‐scan range. Representative EELS spectra of the Mn‐L_2,3_ and O‐K edges acquired from the area with the red block. The Mn‐L_3_ and Mn‐L_2_ peaks exhibit a slight shift toward lower energies of the MO‐pre compared to the MO (Figure 3h), indicating an increased Mn oxidation state. It was demonstrated that the residual MnO(OH) was successfully transformed into α‐MnO_2_ through microwave treatment. Further, the MnO_2_ or MnO(OH) and graphite form a heterogeneous interface where directional charge transfer occurs, resulting in polarization loss. Under microwave irradiation, localized heating generated at the interface promotes the transformation of MnO(OH) to MnO2. Furthermore, studies^[^ ^32^, ^33^ ^]^ have demonstrated that microwave irradiation can effectively lower the activation energy of this transformation process, thereby accelerating the phase transition kinetics.

Consequently, the density functional theory (DFT) method was used to computationally analyze its electron distribution properties. Figure 3i shows the spin density distribution of MnO_2_, which forms an Alpha electron cloud around the atom centered on the atom due to the unsaturation of Mn^4+^ electrons. The O atom presented two distinct scenarios: the Beta electron cloud of the O atom, located at the top corner of the lattice, was distributed toward the bonding direction and was inclined to pair with the Alpha electrons of Mn. Conversely, the O atom located at the common edge of the two octahedra exhibited a pair of p‐orbital antibonding electron clouds, alongside the Beta electrons aligned in the bonding direction. Figure 3j depicts the electron localization function (ELF) of MnO_2,_ wherein the electrons surrounding the atom exhibited pronounced central localization. The ELF between Mn and O was less than 1, indicative of a bonding interaction between the two involving free electron gas. The Mn─O bond between the two cells was analyzed by combining the crystal orbital Hamiltonian population (COHP, Figure S12a, Supporting Information). The bonding and antibonding properties between Mn─O exhibited near‐equality below the Fermi energy level, with ─ICOHP approaching zero. This suggests that the Mn─O bond between the two cells was characterized by a relatively weak interaction. The differential charge density of MnO_2_ is illustrated in Figure S12b (Supporting Information). In this model, functions serve as a donor while O serves as an acceptor, facilitating charge transfer between the two and allowing for partial electron delocalization along the Mn‐O bond connecting the two cells. This interaction improves the polarization characteristics of the MnO_2_ intercell.

By calculating the electron spin density in the crystal, it was determined that the Spin Density of 24.00 ℏ /2 is slightly less than the |Spin Density| of 26.27 ℏ/2. This finding suggests that the MnO_2_ crystal is ferromagnetic or ferrimagnetic. The charged and spin states of the atoms in the crystals were subjected to further analysis using Mulliken analysis and Hirshfeld analysis, as illustrated in Tables S2 and S3 (Supporting Information). The Mn atoms were observed to be predominantly positively charged as electron donors with an upward spin. In contrast, the O atoms were found to be negatively charged as electron acceptors. However, the bond length of planar O to Mn at the octahedral co‐edge (1.88 Å) was noted to be slightly shorter than that of pyramidal O to Mn at the octahedral co‐aperture (1.91 Å). This resulted in a slight increase in charge, with the two exhibiting opposing spin states.^[^ ^34^ ^]^ In conjunction with the total spin state density, it can be determined that MnO₂ is ferrimagnetic.

Moreover, the spin cleavage of O atoms occurs in p orbitals, whereas the spin cleavage of Mn atoms occurs in d orbitals, as can be observed in Table S2 (Supporting Information), which presents the electron population of each atom. Accordingly, the projected density of states (PDOS) on MnO₂ is analyzed as illustrated in Figure 3k,l. The DOS and PDOS of each atom exhibited distinct up‐ and down‐spin probabilities, which contributed to the magnetic properties observed in MnO_2_. The down‐spin electron vibration trends of Mn_d_ and O_p_ were similar in the region below the Fermi energy level, but the up‐spin electrons showed variation. The bias density is employed to calculate the single‐atom magnetic moments of each Mn atom:

(1) $$m=\left(\int_{E_{0}}^{E_{F}}\mathrm{DO}S_{\mathrm{up}}\left(E\right)dE-\int_{E_{0}}^{E_{F}}\mathrm{DO}S_{\mathrm{dn}}\left(E\right)dE\right)\frac{\mu_{B}}{n}$$

where DOS(E) is the density function of the up‐spin and down‐spin fractional wave states below the Fermi energy level. μ_*B*_ denotes the Bohr magneton, where the up‐spin and down‐spin in the electron spin each account for 1/2 of the spin momentum, and thus μ_*B*_  =  ℏ/2, where ℏ is the approximation of Planck's constant. The atomic magnetic moment of Mn was 2.62 *µ*_B_, which is not an integer multiple of the Bohr magneton, indicating that the electrons surrounding the Mn atom were not fully localized at this moment. In conjunction with the Mulliken bond populations (Table S3, Supporting Information), it can be argued that the binding of Mn and O makes the probabilities of their upper and lower spin electrons asymmetric, thus generating the magnetic moment. Furthermore, the ring‐like structure of the material generated magnetically induced currents under the influence of a magnetic field, as illustrated in Figure S21a (Supporting Information). When a magnetic field was applied in the [0 0 1] direction of α‐MnO₂, currents were generated around the Mn and O atoms. The current lines predominantly formed a large loop, as illustrated in Figure S23a (Supporting Information), while smaller loops were observed surrounding each individual atom, as depicted in Figure S23b (Supporting Information). The diatropic and paratropic currents were distributed between the two kinds of Mn─O bonds, respectively (Figure S23c, Supporting Information). Figure S21b (Supporting Information) depicts the induced current lines generated around α‐MnO_2_, with one part of the induced current encircling the Mn─O bond to form a bond current. The remaining portion constitutes a resonant current, which assumes a narrower helix configuration at the point of incidence and expands to a larger helical structure upon interaction with α‐MnO_2_. During this process, the magnetic field energy is converted to electric field energy and dissipated.

### 2.2 Electromagnetic Properties

**Figure 4:**
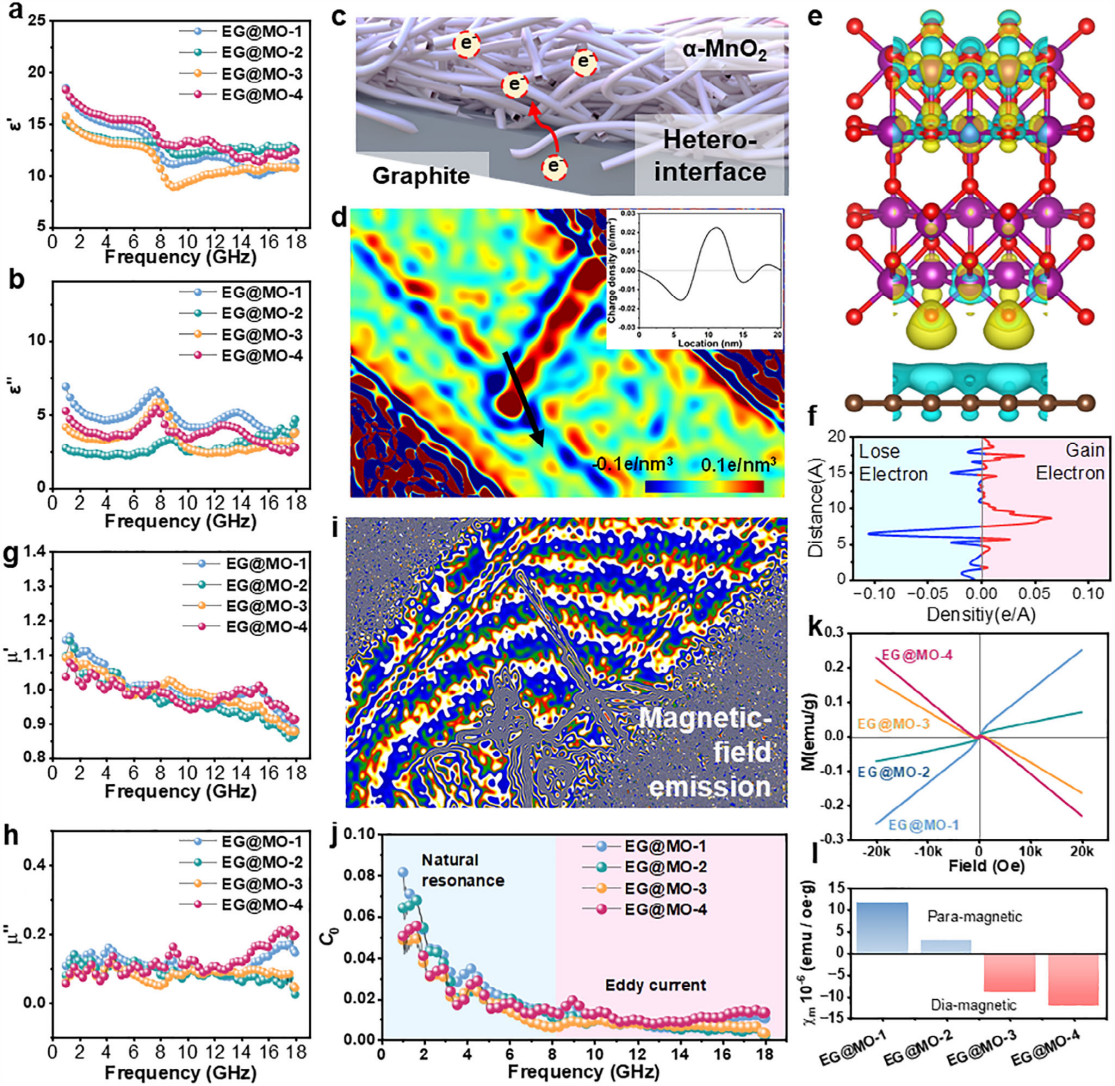
a) real part and b) imaginary part of complex permittivity; c) schematic diagram of the heterointerface between α‐MnO_2_ and Graphite; e) reconstructed charge density hologram; e) electron density differences of interface between α‐MnO_2_ and graphite; f) planar‐averaged electron density differences distribution in c‐axis; g) real part and h) imaginary part of complex permeability; i) reconstructed stray magnetic distribution; j) C_0_; k) M─H magnetization curve and l) dM/dH between ± 2T.

To investigate the electromagnetic wave absorption performance of EG@MO, it was first necessary to analyze its electromagnetic properties. The complex permittivity ε_*r*_ = ε′ − *i*ε′′ is a measure of the dielectric response of a medium to electromagnetic waves. The real part ε′ indicates the medium's ability to respond to the electric field, while the imaginary part ε′′ indicates the loss of the material due to its difficulty in keeping up with the frequency of the alternating electric field. As demonstrated in **Figure** **4**a,b, the real part of the samples showed a decreasing trend as frequency increased. This phenomenon can be linked to the material's hysteresis, which makes it difficult to remain in sync with higher‐frequency electromagnetic waves. From 6 to 10 GHz, the real part of the samples displayed disparate decreasing edges, corresponding to a discernible rising peak in the imaginary part. This indicates that polarization relaxation occurs within this frequency range. Among the samples, EG@MO‐1 exhibited the most pronounced polarization relaxation, which can be attributed to the coexistence of Mn_3_O_4_‐MnO_2_‐graphite phases. The distinct response characteristics of electromagnetic waves in each phase play a significant role in improving polarization relaxation. Likewise, the heterogeneous two‐phase distribution observed in EG@MO‐3 and 4 leads to the emergence of notable polarization characteristics. In contrast, the uniformly distributed nanowires on the surface of EG@MO‐2 result in a weak polarization in this frequency domain. The dielectric loss tangent tan (δ) is a measure of the capacity of a medium to undergo dielectric loss in the presence of electromagnetic waves, as illustrated in Figure S15 (Supporting Information). The enhanced polarization relaxation enables EG@MO‐1 and EG@MO‐3 to exhibit substantial dielectric loss capability within the frequency range of 6–10 GHz, whereas EG@MO‐4 displays a comparable trend but exhibits a relatively lower overall loss value. It is worth noting that EG@MO‐2, despite exhibiting a reduced loss angle tangent at lower frequencies, demonstrates a gradual increase with increasing frequency.

Electron holography further revealed the charge density distribution of the EG@MO. As shown in Figure 4d, the reconstructed charge density distribution clearly demonstrates a distinct heterogeneous interfacial structure. This structure is composed of expanded graphite with a high specific surface area and MnO₂ nanowires with a high aspect ratio. At the interface, the accumulation of positive and negative charges was observed. DFT calculations further support this conclusion. Figure 4e,f show electron density differences of the interface between α‐MnO_2_ and graphite. Graphite acts as the donor while MnO2 acts as the acceptor, and electrons transfer between the interface. Under the influence of external electromagnetic waves, charge migration induces strong interfacial relaxation and polarization, thereby enhancing dielectric loss capability and improving EM scattering.

For the magnetic properties, off‐axis electron holography was employed to investigate the magnetic interactions of EG@MO. In Figure 4i, multiple α‐MnO₂ nanowires emit magnetic field lines with a specific density, forming an interaction network that enables magnetic response. The complex permeability μ_*r*_ = μ′ − *i*μ′′ describes the magnetic response of a medium to electromagnetic waves. The real part μ′ is the magnetic induction generated under an applied magnetic field. As demonstrated in Figure 4g, the real part of EG@MO showed a tendency to decrease as the frequency increased within the 1–8 GHz spectrum, suggesting that it exhibits paramagnetic characteristics in this range. Following this, the EG@MO samples displayed antimagnetic properties after passing through μ′ = 1 at ≈8 GHz. The imaginary part μ′′ represents the hysteresis resulting from the challenge of maintaining the additional magnetic field strength in response to the evolving electromagnetic field at high frequencies. This is illustrated in Figure 4h, which depicts fluctuations between 0 and 0.2 for all samples, except for EG@MO‐1 and 4, which exhibit a slight upward trend at high frequencies. The magnetic loss tangent tan (δ) is a measure of the capacity of a medium to absorb electromagnetic waves, as illustrated in Figure S16 (Supporting Information), demonstrating a comparable trend with μ′′ and exhibiting fluctuations ≈0.1. It was observed that EG@MO‐1 and 4 exhibited an increase at high frequencies, which can be attributed to magnetic unbalance.

At microwave frequencies, magnetic media primarily exhibit natural resonance loss and eddy current loss. The magnetic loss mechanism can be discerned using the magnetic loss coefficient C_0_, as illustrated in Figure 4j. In the frequency range of 1–8 GHz, the C0 of each EG@MO sample exhibits a rapid decline, indicative of the dominance of natural resonance in this region. Natural resonance generally takes place at a specific frequency when atomic magnetic moments are influenced by an external magnetic field. This interaction leads to Larmor precession, which transforms electromagnetic wave energy into thermal energy, ultimately resulting in energy dissipation. In ferromagnetic materials, alongside the atomic magnetic moments, there exist supplementary magnetic fields resulting from crystal orientation and macroscopic magnetization. This phenomenon leads to a broader effective advance frequency compared to that of free electrons. As a result, ferromagnets are capable of generating natural resonance losses across a wider frequency range.

In the frequency range of 9–18 GHz, C_0_ was nearly horizontal, indicating that eddy current losses were mainly generated within this range. In an alternating electromagnetic field, the conductor surface in the plane perpendicular to the normal direction, due to electromagnetic induction, causes electrons to oscillate in a closed loop, thereby forming eddy currents. These currents then convert the electromagnetic wave energy into thermal energy dissipation. Although both natural resonance loss and eddy current loss are fundamentally attributable to the movement of atoms or electrons under the influence of an applied magnetic field, the distinction lies in the disparate locations of the receptor. The natural resonance loss is predominantly associated with magnetic MnO_2_, whereas eddy current loss primarily occurs in the conductive and flat surfaces of graphite. Furthermore, the analysis of the magnetic permeability indicates that μ′ = 1 near 5–9 GHz, which corresponds to the domain wall resonance occurring at this time. Due to the high degree of alignment and narrow diameter of MnO_2_ nanowires, each nanowire can be considered a magnetic domain. These domains oscillate under the influence of an alternating magnetic field, and the frequency of the alternating magnetic field reaches the intrinsic frequency of the domain‐wall vibration when μ′ = 1, which results in resonant absorption.

It is evident that the magnetic properties play a significant role in the electromagnetic wave loss mechanism of EG@MO. To analyze the magnetic properties of EG@MO after the introduction of MnO_2_, a vibrating sample magnetometer (VSM) was employed to assess its magnetic properties, and the M─H curve of the sample is illustrated in Figure 4k. The extent to which the sample is magnetized under the magnetic field is referred to as the magnetic susceptibility χ_*m*_ = *M*/*H* (Figure 4l). In the case of a paramagnetic substance placed in a magnetic field, the magnetic susceptibility is enhanced when χ_*m*_ > 0, and vice versa for a diamagnetic substance, which results in a weakened magnetic susceptibility. EG demonstrates diamagnetic characteristics attributed to its elevated conductivity and stratified architecture. When exposed to an external magnetic field, it undergoes electromagnetic induction, leading to the formation of eddy currents. These eddy currents arise from the advancement of electronic momentum around the magnetic field, consequently producing additional magnetic moments that oppose the direction of the applied magnetic field. As MnO₂ evolves from its paramagnetic or ferrimagnetic state, the magnetic susceptibility of EG@MO‐4 and 3 demonstrates a gradual increase following the addition of a modest quantity of MnO₂; concurrently, the diamagnetism weakens. This phenomenon substantiates the hypothesis that the incorporation of paramagnetic substances and diamagnetism results in a magnetic phase cancellation effect. With the further augmentation of manganese oxide content, the magnetic susceptibility of the samples underwent a reversal, assuming an overall paramagnetic character. The magnetic susceptibility increased from χ_*m*_ = 2.88 × 10^−6^ for EG@MO‐2 to χ_*m*_ = 11.72 × 10^−6^ for EG@MO‐1, and the samples exhibited weak ferrimagnetism in the external magnetic field range of −1k≈+1k Oe (Figure S26, Supporting Information). The electromagnetic waves were canceled out by the induced magnetic fields of the paramagnetic manganese oxides and the diamagnetic graphite, which had opposing directions of magnetic induction. This resulted in an enhancement of the electromagnetic wave absorption properties.

### 2.3 Electromagnetic Wave Absorption

Reflection loss (RL) is a critical parameter for evaluating the electromagnetic wave absorption performance of a material, as illustrated in Equations S7–S10 (Supporting Information). A lower RL corresponds to a lower intensity of the reflected electromagnetic wave, indicating a greater absorption capability. It is generally accepted that an RL of less than −10 dB is sufficient to achieve effective absorption of electromagnetic waves, and this frequency range is referred to as the effective absorption bandwidth (EAB). According to transmission line theory, when a medium receiving electromagnetic waves is impedance matched, its ability to absorb electromagnetic waves is at its greatest. This is indicated by a Z‐index (Equation S11, Supporting Information) close to 1, which signifies a reliable electromagnetic wave absorption performance. In addition to Z, studies^[^ ^35^, ^36^ ^]^ have suggested that phase information should be considered when discussing the degree of impedance matching, i.e., using |Z_in_‐Z_0_| as a measure.

**Figure 5:**
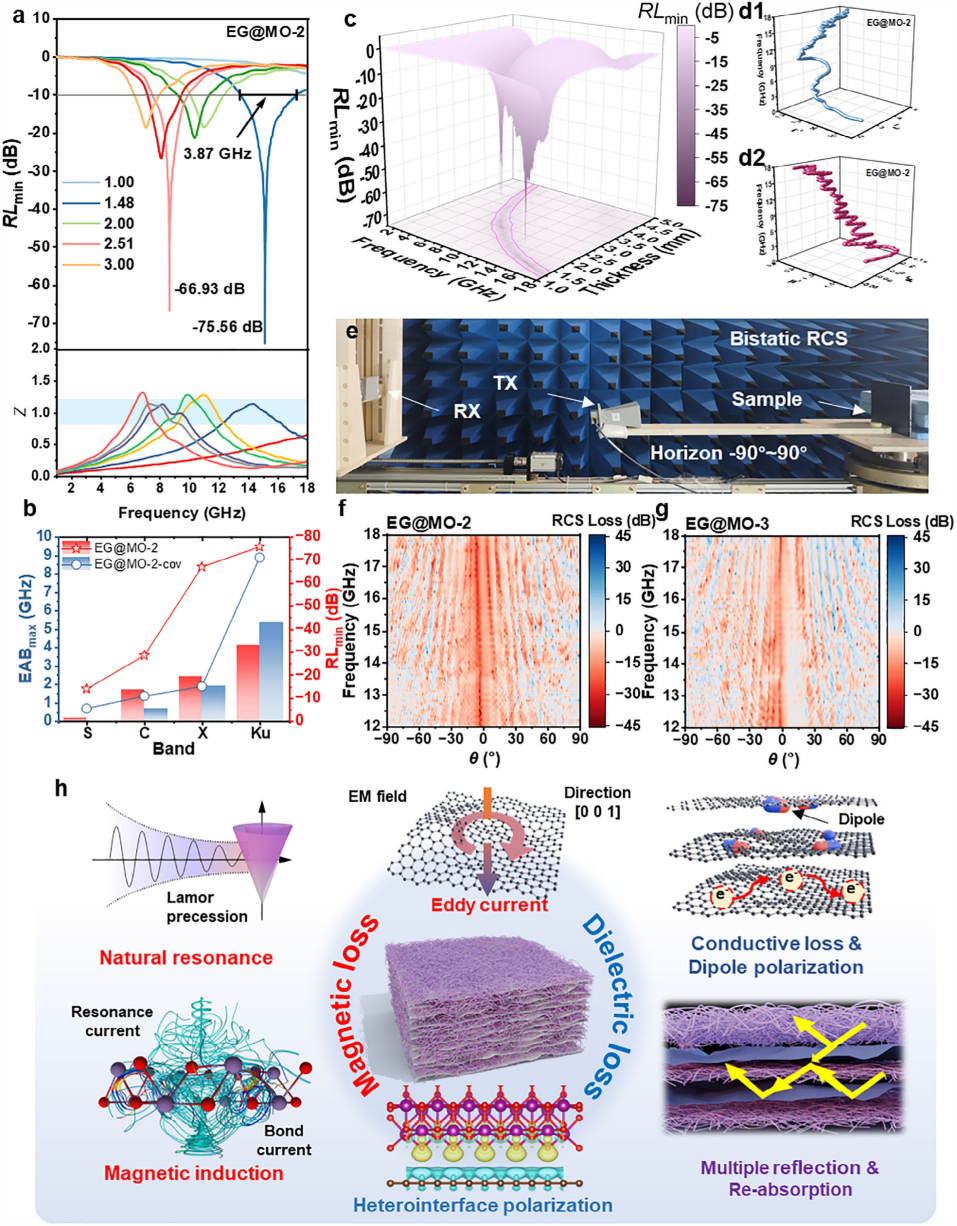
EM absorptions of EG@MO. a) RL at various thicknesses and impendence matching Z of EG@MO‐2; b) comparison of RL between EG@MO‐2 and EG@MO‐2‐cov; c) RL as a function of thickness and frequency of EG@MO‐2; d1) dielectric and d2) magnetic Cole–Cole plot of EG@MO‐2; e) schematic of the bistatic radar cross section (RCS) measurement set‐up; RCS losses distribution for f) EG@MO‐2 and g) EG@MO‐3; h) schematic diagram of the electromagnetic wave absorption mechanism.

**Figures** **5**a,c and S17b1–b4 (Supporting Information) illustrate the RL and the impedance matching index Z and |Z_in_‐Z_0_| (Figure S38, Supporting Information) for each EG@MO sample. The impedance mismatch, indicated by a Z‐value of less than 1, was observed in EG@MO‐1, which contained the lowest amount of graphite. Consequently, this sample exhibited a diminished performance in electromagnetic wave absorption, registering just below −10 dB. EG@MO‐2 exhibited the optimal electromagnetic wave absorption performance, with an RL_min_ of −75.56 dB and an EAB of 3.87 GHz for the electromagnetic wave of 15.16 GHz at a thickness of 1.48 mm. Additionally, it exhibited an RL_min_ of −66.93 dB for an 8.68 GHz electromagnetic wave with an EAB of 2.44 GHz at a thickness of 2.51 mm. It is worth noting that the difference between the two strongest electromagnetic wave absorption frequencies was measured at 1.74, a value that is approximately equal to the difference between the Larmor frequencies of Mn and O atoms of 1.82 (Equation S14, Supporting Information). This suggests that atomic spin resonance may occur at these two frequencies.

The comparison of RL and EAB between EG@MO‐2‐cov and EG@MO‐2 across different frequency bands is shown in Figure 5b. EG@MO‐2 shows better RL performance across all frequency bands and exhibits broader EAB in the S─X band, while EG@MO‐2‐cov achieves wider EAB in the Ku band. As illustrated in Figure S35b (Supporting Information), the RLmin of EG@MO‐2‐cov reaches −70.93 dB at a thickness of 1.71 mm, while EG@MO‐2 achieves −75.56 dB at a reduced thickness of 1.48 mm. This demonstrates that the microwave‐activated sample exhibits enhanced electromagnetic wave absorption performance at smaller thicknesses. This performance limitation originates from the structural collapse of EG during the hydrothermal synthesis of EG@MO‐pre (Figure S36, Supporting Information), where conventional heating fails to restore its expanded morphology. In contrast, microwave activation enables complete structural restoration, forming multilayered honeycomb architectures that enhance electromagnetic wave attenuation through optimized multiple reflection losses. The EAB of the two samples is shown in Figure S43 (Supporting Information). EG@MO‐2 exhibits an EAB of 4.14 GHz at 1.44 mm thickness, whereas EG@MO‐2‐cov achieves a broader EAB of 5.34 GHz at 1.80 mm thickness. Although EG@MO‐2‐cov shows wider EAB at specific thicknesses, EG@MO‐2 maintains more uniform EAB performance across a broader range of frequencies and thicknesses.

EG@MO‐3 demonstrated a marginally lower capacity for electromagnetic wave absorption, recording an RL_min_ of −46.13 dB for the 16.42 GHz electromagnetic wave at a thickness of 1.48 mm, and an RL_min_ of −54.80 dB at a thickness of 2.65 mm, which is associated with the 9.59 GHz electromagnetic wave. In comparison to the currently published carbon/magnetic composite electromagnetic wave‐absorbing materials (Figure S29, Supporting Information), EG@MO‐2 exhibited an exemplary electromagnetic wave‐absorbing performance. Further, the reflectivity Γ of EG@MO‐2 at 1.5 mm thickness was measured using the arch‐method test, as shown in Figure S40 (Supporting Information). EG@MO‐2 achieved a minimum Γ of −15.91 dB at 10.85 GHz, showing good agreement with the corresponding RL result.

The Cole‐Cole dielectric curve describes the response of the dielectric properties of a material to the absorption of electromagnetic waves. The polarization loss is dominated by Debye relaxation at the moment when the curve is semicircular, and conduction loss occurs when the curve is close to a straight line. The dielectric Cole–Cole curves of the four samples are illustrated in Figure 5d, 1 and Figure S17c1–c4 (Supporting Information). There are distinct semicircles and straight lines between the different samples, which signify the collective influence of polarization loss and conduction loss. Similarly, the magnetic Cole–Cole curve can be employed as a reference for the analysis of the magnetic loss capability of EG@MO. In the case of magnetic permeability at the point of resonance, the Landau–Lifshitz–Gilbert equations (Equations S15–S17, Supporting Information) apply, whereby a semicircular shape is observed on the magnetic Cole–Cole diagram. The magnetic Cole–Cole curves of the samples are presented in Figure 5d, 2 and Figure S17d1–d4 (Supporting Information). EG@MO‐2 exhibits multiple semicircles, indicative of significant magnetic resonance loss. In contrast, EG@MO‐1 displays smaller semicircles, while EG@MO‐3 exhibits almost no semicircles, suggesting relatively weak magnetic resonance loss. The findings indicate that the absorption of electromagnetic waves in EG@MO can be attributed to a synergistic effect involving dielectric loss, which includes polarization relaxation and transmission loss, as well as magnetic loss, which is comprised of magnetic resonance contributed by MnO₂ and eddy current loss associated with graphite.

Further, the radar cross section (RCS) of aluminum plates coated with ≈1.5 mm thick EG@MO‐2 and EG@MO‐3 was respectively measured using a bistatic radar system (Figure 5e). Figure 5f,g show the RCS losses (RCS_Sample_ – RCS_PEC_) of both samples, showing their good radar wave absorption performance. At 15.8 GHz and reflection angles of 0°, EG@MO‐2 exhibited an RCS loss of ‐23.46 dBsm, while EG@MO‐3 showed −15.21 dBsm. To further investigate the RCS behavior of these materials, electromagnetic field simulations were conducted and analyzed in the Supporting Information. Among the simulated results, EG@MO‐2 displayed the lowest RCS value of −24.34 dBsm at 0° reflection angle with a 1.48 mm thickness. Notably, the experimental measurements and simulation results exhibited strong consistency.

The process of electromagnetic wave absorption fundamentally involves the transformation of electromagnetic waves into thermal energy. Consequently, the variation in temperature of a material subjected to microwave irradiation at varying power levels serves as an indicator of its actual capacity for electromagnetic wave absorption. Figure S27a–c (Supporting Information) shows the temperature variation and heating/cooling rates of each microwave‐treated sample under 400 W microwave irradiation. Among them, EG@MO‐2 had the highest heating and cooling rates of 105.74 and −81.33 °C s^−1^, respectively. The temperature variation rates of EG@MO‐1 and EG@MO‐3 were close to each other, but the highest temperature of EG@MO‐1, 411.4 °C, was significantly lower than that of EG@MO‐3, 534.9 °C.

The electromagnetic wave absorption of EG@MO was primarily achieved through the coupling of the magnetic and dielectric properties of α‐MnO₂ and EG, as illustrated in Figure 5h. On the one hand, the electron spin property of α‐MnO_2_ under the electromagnetic field causes it to generate a natural resonance (Lamor progression), which converts the electromagnetic wave into heat and dissipates it. In contrast, graphite generates an induced current under the electromagnetic field and converts it into Joule heat. These two processes represent different approaches to magneto‐thermal conversion. Furthermore, the wire‐like α‐MnO_2_ exhibited distinctive magnetic confinement characteristics. The incident magnetic field propagates along the axial direction of the nanowire, while the electromagnetic field propagates in diverse directions within EG@MO, thereby enhancing the magnetic loss. On the other hand, EG shows strong electrical conductivity, and the introduction of α‐MnO_2_ reduces the skin effect on the surface of EG. During the transformation of MnO(OH) to α‐MnO_2_, several defects are formed, which enhance impedance matching and promote the absorption of electromagnetic waves. Additionally, there is a notable disparity in dielectric characteristics between EG and α‐MnO_2_, which facilitates the polarization of EG@MO in response to electromagnetic radiation, resulting in relaxation loss. Moreover, from the perspective of structural design, the α‐MnO_2_ nanowires create a distinctive fabric‐like structure on the surface of the multilayer graphite. This structure enables the electromagnetic wave to resonate within the cavity formed between the graphite sheet layers and to undergo multiple reflections when penetrating the α‐MnO_2_ nanowires. This enhances the loss‐matching performance of the electromagnetic wave. The optimal impedance matching and loss matching, as well as the excellent electromagnetic wave absorption performance, can be attained by adjusting the ratio of EG to α‐MnO_2_. This facilitates the two‐way regulation of dielectric loss and magnetic loss.

## 3 Conclusion

Based on the synergistic regulation strategy of dielectric and magnetic properties, this study presents a method for preparing composites with efficient electromagnetic wave absorption capabilities. The α‐MnO_2_ nanowires were synthesized in situ on the surface of expanded graphite via a microwave hydrothermal process, forming a fabric‐like encapsulated structure. The transformation of manganese oxides from biphasic to monophasic, from low‐valent to high‐valent states, and from nonmagnetic to magnetic phases was achieved through rapid microwave activation. The dielectric and magnetic properties of the composites were adjusted by varying the α‐MnO_2_ content, enabling excellent impedance matching and outstanding electromagnetic wave absorption. The unique functionalized multilayer fabric structure of EG@MO facilitates multiple reflections and dissipation of electromagnetic waves within the material, thereby enhancing the efficiency of electromagnetic wave absorption. The EG@MO‐2 composites demonstrated a reflection loss of −75.56 dB at a filler ratio of 7 wt.% and a thickness of 1.48 mm. These remarkable characteristics position the material as a strong candidate for use in electromagnetic protection and stealth technology applications.

## 4 Experimental Section

### Synthesis of EG@MO—Preparation of Expanded Graphite

Expanded graphite was prepared using microwave expansion wherein 1 gram of Na_2_S_2_O_8_ (analytical grade, Aladdin Scientific Corp.) was dissolved in 10 grams of H_2_SO_4_ (analytical grade, Aladdin Scientific Corp.). Once completely dissolved, 1 g of scaled graphite (50#, Aladdin Scientific Corp.) was added and stirred for 5 min, then transferred to a 40 mL Teflon autoclave. The Teflon autoclave was irradiated with microwaves at 800 W for 30 s to complete the intercalation process. The interpolated mixture was diluted to a pH of ≈7 with deionized water, filtered under vacuum, and dried at 60 °C to obtain expandable graphite. The expandable graphite was evenly distributed on a ceramic disk and irradiated with 800 W microwaves for 30 s to achieve expansion and remove the sulfur to obtain expanded graphite.

### Synthesis of EG@MO—Preparation of EG@MO

EG@MO‐Pre was synthesized via a microwave hydrothermal method. Initially, 0.16 g KMnO₄ (AR, Aladdin Scientific Corp.), 3.28 g urea, and 0.02 g KCl (AR, Aladdin Scientific Corp.) were sequentially dissolved in 20 mL DI water. A specified amount of EG (as indicated in Table S1, Supporting Information) was then added and thoroughly stirred for 20 min before being transferred into a 40 mL Teflon autoclave. The reaction was heated to 180 °C using a microwave digestion system (MWD 620, METASH) and maintained for 2 h. After the reaction was completed, the residual K^+^ ions were removed by rinsing with DI water, and the precursor EG@MO‐Pre was obtained following filtration and drying at 60 °C. Then, the precursor was irradiated with a microwave at 500 W for 10 s, resulting in the EG@MO. The processing temperature was monitored by an infrared sensor, and the microwave power is controlled by a PID controller after reaching the target temperature.

### Synthesis of EG@MO—Characterization

X‐ray diffraction (XRD, SmartLab SE, Rigaku) equipped with Cu K‐α source (8.0478 keV, λ = 1.5406 Å) was utilized to examine the crystal properties of EG@MO. The scanning was performed from 2θ = 10° to 90° with a scan step of 5° min^−1^. A Rietveld refinement was performed with GSAS‐II.^[^ ^37^ ^]^ To analyze the chemical states of EG@MO, X‐ray photoelectron spectroscopy (XPS, K‐Alpha, ESCALAB Xi+, Thermo Scientific) equipped with an Al Kα source (*h_v_* = 1486.6 eV, beam size = 400 µm) was employed. The scanning was conducted five times at a pressure below 2.0 × 10^−7^ mbar. For the survey spectrum, the pass energy was 150 eV with a scanning resolution of 1 eV. For the high‐resolution spectrum, the pass energy was 50 eV with a scanning resolution of 0.1 eV. The results were calibrated with 284.80 eV as a standard reference for C_1s_. Moreover, the Mn K‐edge X‐ray absorption fine structure analysis (XAFS) was performed with Si(111) crystal monochromators at the BL11B beamlines at the Shanghai Synchrotron Radiation Facility (SSRF, Shanghai, China). A 4‐channel Silicon Drift Detector (SDD) Bruker 5040 was used to record the XAFS spectra in transmission mode at room temperature. Both the two samples and the standard samples were measured in the same batch. The XAFS data were processed and analyzed with the Demeter software suite (ATHENA, ARTEMIS, and HEPHAESTUS).^[^ ^38^ ^]^

To analyze the morphology of EG@MO, the samples were ground and uniformly applied to the conductive tape, then observed using scanning electron microscopy (SEM, Nova Nano 450, FEI) at an accelerating voltage of 15 kV. To gain a more comprehensive insight into the morphology and crystalline characteristics, the samples were finely ground and dissolved in ethanol, ultrasonicated for 10 min, and then dropped onto a carbon film‐supported copper hexagonal mesh grid for high‐resolution transmission electron microscopy (HR‐TEM, Talos F200x, FEI, 200 kV). Further, a high‐angle annular dark‐field scanning transmission electron microscopy (HAADF‐STEM) was performed with an aberration‐corrected transmission electron microscope (AC‐TEM, Titan Cubed Themis 300, FEI, 300 kV). The charge density hologram and stray magnetic distribution were measured with an off‐axis Lorentz transmission electron microscope (JEM‐2100F, JEOL).

To analyze the electromagnetic wave absorption properties of EG@MO, the transmission line method was used for the measurements. To prepare the mixture, EG@MO was ground to a particle size of 0.10 mm, mixed with melted paraffin at a ratio of 7 wt. % of EG@MO, comparisons of different concentrations were discussed in detail in the Supporting Information. To create sample rings, the mixture was placed in a mold and compressed for 10 s at a pressure of 10 MPa (*d*_inner_ = 3.04 mm, *d*_outer_ = 7.00 mm, *h* = 2.00 mm). The rings were then placed in a coaxial airline (85051–60007, Keysight) and connected to a vector network analyzer (E5080B, Keysight) to measure their complex permittivity and permeability.

### Numerical Simulations—DFT Simulations

First‐principles calculations were conducted based on the framework of density functional theory using CASTEP^[^ ^39^ ^]^. This involved the application of the exchange‐correlation potential as defined by the Perdew–Burke–Ernzerhof method,^[^ ^40^ ^]^ along with the generalized gradient approximation, utilizing the projector‐augmented plane‐wave method. The cut‐off energy for the plane wave was set to 571.40 eV, and the tolerance limit of the self‐consistent field was 10^−6^ eV atom^−1^. Furthermore, magnetically induced currents were calculated with the B3LYP/6‐311G* method, then processed and analyzed with the GIMIC software.^[^ ^41^ ^]^

### Numerical Simulations—Electromagnetic Simulation

The CST Studio Suite (Dassault Systèmes) was used to conduct the electromagnetic response simulation within the 1–18 GHz waveband range. As illustrated, the model was made of a square perfect electric conductor (PEC) plate (2 mm × 180 mm × 180 mm). The PEC was coated with a specified thickness of the sample. Electromagnetic waves were injected from θ = 0° and 180° (perpendicular to the sample and PEC, respectively), and their radar scattering cross sections from −90° to 90° were analyzed.

## Conflict of Interest

The authors declare no conflict of interest.

## Supporting information



Supporting Information

Supplemental Video 1

Supplemental Video 2

## Data Availability

The data that support the findings of this study are available from the corresponding author upon reasonable request.
