# Supplementary material for: Microwave‐Driven Dielectric‐Magnetic Regulation of Graphite@α‐MnO2 Toward Enhanced Electromagnetic Wave Absorption
Source: Adv Sci (Weinh). 2025 Jun 20;12(34):e04489. doi: 10.1002/advs.202504489 (PMC12442661; doi:10.1002/advs.202504489)
Supplement: Supplementary file 1 — Supporting Information [file ADVS-12-e04489-s002.pdf]

ADVANCED SCIENCE
Open Access

Supporting Information

for *Adv. Sci.*, DOI 10.1002/advs.202504489

Microwave-Driven Dielectric-Magnetic Regulation of Graphite@α-MnO_2 Toward Enhanced Electromagnetic Wave Absorption

Junyu Lu, Lei Xu, Cheng Xie, Chang Zhang, Zhaohui Han, Yiyao Ren and Renchao Che**

Supplementary Information for

Microwave-Driven Dielectric-Magnetic Regulation of Graphite@α-MnO_2 towards Enhanced Electromagnetic Wave Absorption

Junyu Lu [a, b,†], Lei Xu [a, b, c, *], Cheng Xie [a, b], Chang Zhang [d,†], Zhaohui Han [a, b, c], Yiyao Ren [a, b], Renchao Che [d, *]

a. Faculty of Metallurgical and Energy Engineering, Kunming University of Science and Technology, Kunming 650093, PR China.

b. National Local Joint Laboratory of Engineering Application of Microwave Energy and Equipment Technology, Kunming 650093, PR China

c. The Key Laboratory of Unconventional Metallurgy, Ministry of Education, Kunming 650093, PR China

d. Laboratory of Advanced Materials, Shanghai Key Lab of Molecular Catalysis and Innovative Materials, Academy for Engineering and Technology, Advanced Coatings Research Center of Ministry of Education of China, Fudan University, Shanghai 220438, PR China.

† These authors contributed to the work equally.

* Corresponding author: E-mail address: xu_lei@kust.edu.cn (Lei Xu); rcche@fudan.edu.cn (Renchao Che)

This file includes:

Supplementary Text

Equations S1 to S26

Tables S1 to S4

Figures S1 to S43

Supplementary References

Supplementary videos in separate file

Supplementary Text

Synthesis and properties of EG@MO

MnO_2 is the thermodynamically most stable state of manganese in the MnO_4^- aqueous solution system. During hydrothermal processes, MnO_4^- oxidizes water and urea produced MnO_2.

$$4KMnO_4+2H_2O \rightarrow 4MnO_2+4KOH+3O_2 \quad (1)$$

$$4KMnO_4+2CH_4N_2O \rightarrow 2K_2CO_3+4MnO_2+2NH_3+2NO_2+H_2 \quad (2)$$

Part of the MnO2 transformed into MnO(OH) during the hydrothermal process due to the self-generated pressure in the autoclave.

$$4MnO_2+2H_2O \rightarrow 4MnO(OH)+O_2 \quad (3)$$

Ammonium carbamate and ammonium ions are produced from the decomposition of urea during hydrolysis:

$$CH_4N_2O+H_2O \rightarrow NH_2COO^-+NH_4^+ \quad (4)$$

These ions will further react with MnO(OH) to Mn3O4:

$$3MnO(OH)+2NH_2COO^-+3NH_4^+ \rightarrow Mn_3O_4+5NH_3+2H_2O+2CO_2 \quad (5)$$

The X-ray diffraction patterns of the products at various reaction times are presented in Figure S1. It was observed that no significant crystallization occurred before 60 minutes, while both α-MnO_2 and MnO(OH) were formed after 90 minutes The degree of MnO(OH) crystallization increased as a result of the prolonged MnO_2 hydrolysis reaction and the extended reaction time. The XRD at different treatment temperatures is shown in Figure S3. The characteristic peaks of EG@MO-2 appeared at 28.74° and 37.63°, corresponding to the (130) and (211) crystal planes of α-MnO_2, proving that $KMnO_4$ was transformed into α-MnO_2 nanowires at this time. As Mn content continued to increase, the characteristic peaks of α-MnO_2 in EG@MO-1 were

observed; however, the characteristic peaks corresponding to the (112) and (211) crystal planes of Mn_3O_4 emerge at angles of 28.92° and 36.10°, respectively.

Specifically, in this transformation process, the microwave oxidation roasting treatment utilized the electromagnetic wave absorbing ability of EG@MO-Pre to convert MnO(OH) to α-MnO_2 under rapid thermal shock. The thermogravimetric differential curves and XRDs of the manganese oxide precursor MO-Pre at different temperatures are shown in Figures S5 and S6. The free and bound water present in EG@MO-Pre was eliminated within the temperature range of room temperature to 170°C, resulting in the transformation of α-$MnO_2(H_2O)$ into α-MnO_2. In the temperature range of 170 to 300 °C, MnO(OH) was converted to α-MnO_2, resulting in the disappearance of the MnO(OH) peak and a corresponding increase in the intensity of the α-MnO_2 peak at 300 °C. At the temperature range between 300 and 500 °C, the intensity of the α-MnO_2 peak and crystallinity both increased. Above 500 °C, the α-MnO_2 nanowires began to soften, and when the temperature was raised to 650 °C, they were converted into Mn_2O_3 nanoparticles. Therefore, maintaining the treatment temperature within the range of 300 to 500 °C facilitates the uniform transformation of manganese oxides and improves the crystallinity of α-MnO_2, as evidenced by the Raman spectra (Figure S8). The heating characteristics of the microwave facilitated the direct heating of the MO attached to the EG. At a controlled microwave power of 400 W, the temperature increased to 445.3 °C in 10 seconds (Figure S9), resulting in the rapid conversion of EG@MO-Pre to EG@MO.

The chemical state of MO did not change significantly after binding to EG. The

chemical state of EG@MO-2 was examined using XPS, as illustrated in Figure S13. In the 2p orbitals of Mn, a pair of spin–electron cleavage peaks were identified. The peaks corresponding to $2p_{3/2}$ and $2p_{1/2}$ were detected at 642.3 eV and 653.9 eV, respectively, with a peak separation of 11.6 eV. The results substantiated that the chemical state of Mn in EG@MO-2 was positively tetravalent, in alignment with the chemical state of MnO_2. The O 1s orbital was dominated by Mn-O (530.0 eV) and O-Mn-O (529.4 eV) peaks, indicative of manganese oxides. Furthermore, characteristic peaks corresponding to H-O-H and C-O were detected, indicating the adsorption of water and CO_2, respectively.

The surface functional groups of EG@MO were analyzed by FT-IR, as illustrated in Figure S15. The weak peaks observed between 1700 and 1500 cm^{-1} are attributed to the C=C bending vibration of carbon rings in graphite, while the peaks present between 1085 and 1050 cm^{-1} are indicative of C-O stretching vibrations, suggesting that localized oxidation may have occurred on the graphite surface. The strong double peak observed near 2350 cm^{-1} is attributed to the O=C=O stretching vibration of adsorbed CO_2. This peak was not observed in EG@MO-1 and 2, but became evident in 3 and 4, and exhibited an enhanced intensity with increasing EG content. This phenomenon can be attributed to the fact that at low EG content, the active functional groups on the graphite surface underwent complete oxidation by $KMnO_4$, resulting in the occupation of these sites by the resulting MnO_2 nanowires. This process renders the surface less accessible, thereby impeding the adsorption of CO_2 molecules. As the EG content increased, the nanowires failed to completely cover the graphite surface, resulting in

exposed areas that could adsorb CO_2. The peaks detected in the range of 600 to 400 cm^{-1} are associated with the vibration of the Mn-O bond in MnO_2, which contrasts with the peaks related to CO_2 adsorption. These peaks are more pronounced in EG@MO-1 and 2 at low EG content. This result substantiates the hypothesis that the production of MnO_2 at low EG content occupies the surface of graphite, forming a protective layer that encapsulates the expanded graphite and effectively insulates it from CO_2 adsorption.

Microwave activation

Figure S20 shows the temperature change of the precursor EG@MO-2-Pre during three microwave irradiation cycles with microwave powers of 400 W (R1), 600 W (R2), and 400 W (R3). In R1, the temperature of the sample increased from 33.4 °C to 121.2 °C in 46 seconds, at which point further increase ceased, indicating the limited heating capacity of 400 W microwave irradiation for EG@MO-2-Pre. Following a period of cooling to room temperature and subsequent entry into R2, the sample exhibited a rapid initial heating rate, increasing from room temperature to 124.1 °C within 15 seconds under 600 W microwave irradiation. Subsequently, the temperature of the sample increased gradually to 256.7 °C within 68 s. At this stage, the bound water was eliminated, leading to the transformation of MnO(OH) into MnO2, which caused a rapid rise in temperature to 558.7 °C (the upper detection limit) within a span of 6 seconds. This indicates that EG@MO-2-Pre had undergone a complete conversion to EG@MO-2. After cooling to room temperature, the sample was transferred to R3. Upon re-irradiation with 400 W microwaves, a significant heating effect was observed, with the temperature increasing from room temperature to 558.7 °C (upper detection limit)

within 4 seconds. The R1 and R3 phases exhibited significantly disparate heating characteristics, even when subjected to the same microwave power, thereby demonstrating that the thermal reaction process under microwave irradiation of a given power markedly influences its electromagnetic wave absorption capacity.

Microwave absorption properties

The attenuation constant α describes the attenuation capacity of electromagnetic waves, written as:

$$\alpha = \frac{\sqrt{2}\pi f}{c}\sqrt{(\mu''\varepsilon''-\mu'\varepsilon')+\sqrt{(\mu''\varepsilon''-\mu'\varepsilon')^2+(\mu''\varepsilon'-\mu'\varepsilon'')^2}} \tag{6}$$

where, beside the complex permittivity $\varepsilon_{\mathrm{r}} = \varepsilon' - i\varepsilon''$ and complex permeability $\mu_r = \mu' - i\mu''$, f is the frequency of electromagnetic waves and c is the speed of light in vacuum.

The reflection loss (RL) describes the ability of a material to absorb electromagnetic waves, it was calculated based on transmission line theory, which could be written as:

$$\mathrm{RL} = 20\log|\Gamma| = 20\log\left|\frac{Z_{in}-Z_0}{Z_{in}+Z_0}\right| \tag{7}$$

In the lossy transmission line, input impedance $Z_{in} = Z\tanh(\gamma''d)$, where d is the thickness of the material, the propagation constant γ'' is

$$\gamma'' = j\omega\sqrt{\mu_0\mu_r\varepsilon_0\varepsilon_r} = j(2\pi f/c)\sqrt{\mu_r\varepsilon_r} \tag{8}$$

and the characteristic impedance Z is

$$Z = \sqrt{\frac{\mu_0\mu_r}{\varepsilon_0\varepsilon_r}} = Z_0\sqrt{\frac{\mu_r}{\varepsilon_r}} \tag{9}$$

Hence the input impedance can be written as

$$Z_{in} = Z_0\sqrt{\frac{\mu_r}{\varepsilon_r}}\tanh\left[j\frac{2\pi fd}{c}\sqrt{\mu_r\varepsilon_r}\right] \tag{10}$$

where Z_0 is the free space impedance, d is the thickness.

The normalized input impedance Z describes the impedance matching, written as:

$$Z = \left|\frac{Z_{in}}{Z_0}\right| \tag{11}$$

The matching thickness tm can be calculated from the quarter theory, written as:

$$t_m = \frac{n\lambda}{2} = \frac{nc}{4f_m\sqrt{|\varepsilon_r\mu_r|}}(n = 1,3,5...) \tag{12}$$

According to the theory of Debye's relaxation model, ε'' considered as the sum of conduction loss ε_c and polarization loss ε_p, written as:

$$\varepsilon'' = \frac{\varepsilon_S - \varepsilon_\infty}{1+\omega^2\tau^2}\omega\tau + \frac{\sigma}{\omega\varepsilon_0} \tag{13}$$

where σ is the conductivity, ε_S and ε_∞ is the static permittivity and high-frequency permittivity limit; ε_0 is the vacuum permittivity, with a value of 8.854×10^{-12} $CV^{-1}m^{-1}$; τ is the relaxation time.

The Larmor frequency f_0 refers to the resonance frequency that a particular spin will have at a certain primary magnetic field strength B_0 and is written as:

$$f_0 = \frac{\gamma}{2\pi}B_0 \tag{14}$$

where γ is the gyromagnetic ratio, for MnO_2, it has $\gamma_{Mn} = 6.61\times10^7$ rad T^{-1} s^{-1} and $\gamma_O = -3.63\times10^7$ rad T^{-1} s^{-1}. Since the B_0 is consistence in this case, the ratio of f_0 can be considered as $\gamma_{Mn}/\gamma_O = 1.82$.

Landau–Lifshitz–Gilbert equations described the relationship of the permeability for magnetic resonance:

$$\mu' = C + \sum_{i=1}^{n} \chi_i \frac{1-(f/f_{ri})^2(1-\alpha_i^2)}{\left[1-(f/f_{ri})^2(1-\alpha_i^2)\right]^2 + 4\alpha_i^2(f/f_{ri})^2} \quad (15)$$

$$\mu'' = \sum_{i=1}^{n} \chi_i \frac{(f/f_{ri})^2\alpha_i\left[1+(f/f_{ri})^2(1+\alpha_i^2)\right]}{\left[1-(f/f_{ri})^2(1-\alpha_i^2)\right]^2 + 4\alpha_i^2(f/f_{ri})^2} \quad (16)$$

where f is the wave frequency, f_{ri} is the resonance frequency, α_i is the damping constant, χ_i is the resonance intensity. Thus,

$$(\mu_i')^2 + (\mu_i'')^2 = \chi_i^2 \frac{1+(f/f_{ri})^2\alpha_i^2}{\left[1-(f/f_{ri})^2(1+\alpha_i^2)\right]^2 + 4\alpha_i^2(f/f_{ri})^2} \quad (17)$$

Correlation of electromagnetic properties with S-parameters

The correlation between permittivity, permeability and S-parameters can be described by the Nicolson-Ross-Weir relation:

$$\Gamma = X \pm \sqrt{X^2 - 1} \quad (18)$$

where

$$X = \frac{1-(S_{21}^2 - S_{11}^2)}{2S_{11}} \quad (19)$$

Then the transmission coefficient can be written as

$$T = \frac{S_{11} + S_{21} - \Gamma}{1-(S_{11}+S_{21})\Gamma} \quad (20)$$

Thus the permeability and permittivity can be written as:

$$\mu = \frac{\lambda_{0g}}{\Lambda}\left(\frac{1+\Gamma}{1-\Gamma}\right) \quad (21)$$

$$\varepsilon = \frac{\lambda_0^2\left(\frac{1}{\Lambda^2} + \frac{1}{\lambda_c^2}\right)}{\mu} \quad (22)$$

where

$$\frac{1}{\Lambda^2} = -\left(\frac{1}{2\pi d}\ln\frac{1}{T}\right)^2 \quad (23)$$

and λ_0 is the free-space wavelength, λ_{0g} is the guided mode wavelength of the unfilled transmission line, λ_c is the cutoff wavelength of the unfilled transmission line.

Radar cross section simulation

The numerical simulation results of the radar reflectance cross section (RCS) of EG@MO are presented in Figures S23 to S25. A smaller RCS indicates that the material exhibits a reduced intensity of reflection under electromagnetic wave irradiation, thereby demonstrating a higher electromagnetic wave absorption capability. The RCS losses of the four samples was simulated as shown in Figure S25. EG@MO-2 had the lowest RCS, measuring -24.34 dBsm and -35.69 dBsm at reflection angles of 0° and 30°, respectively. EG@MO-3 showed a slightly lower RCS, resulting in RCS_0 = -20.28 dBsm and RCS_{30} = -31.95 dBsm, respectively. According to the reflection loss results, EG@MO-1 and EG@MO-4 had even lower RCS. Furthermore, the RCS loss distributions of EG@MOs at various thickness and frequency were showed in Figure S37. EG@MO-1 demonstrates optimal performance at thinner configurations (<2 mm), whereas EG@MO-2 exhibits broadband effectiveness across both low and high thickness ranges. In contrast, EG@MO-3 and EG@MO-4 require substantially greater thicknesses to achieve effective microwave absorption.

Electromagnetic properties and reflection loss at various EG@MO-2 concentrations

To determine the optimal concentration of EG@MO in paraffin, RL and dielectric constant/magnetic permeability variation data were tested at 3%, 5%, 7%, 9%, and 11% (mass concentration), as shown in Figures S32-33. As the concentration of EG@MO increases, the real and imaginary parts of the dielectric constant rise, and the RL increases from -48 dB to -75 dB, indicating that the increase in concentration increases

the efficiency of electromagnetic wave absorption, but a significant decrease occurs when the concentration is greater than 9 wt.%. This is due to the fact that too much EG@MO forms a conductive network in the paraffin wax, and an impedance mismatch occurs in the transmission line that prevents the absorption of electromagnetic waves, which is also proposed by Tang et al. [1]. Resistivity/conductivity tests (Table S4) can further support this conclusion, with resistivity ≥109 Ω·m at concentrations of 3, 5, and 7%, but resistivity ≤102 Ω·m for samples at concentrations of 9 and 11%.Based on the above, it can be assumed that the optimal uptake efficiency is available at a concentration of 7% for EG@MO.

FMCW radar test

As shown in Figure S42 (a), a 24 GHz Frequency Modulated Continuous Wave (FMCW) radar was set up at a height of 1.5 m above the ground to detect the moving speed and position of a back and forth walking target (human) directly in front of it. A 2 mm thick PMMA plate and PDMS with 7 wt.% EG@MO-2 were placed 20 cm in front of the radar, respectively. The results are shown in Figure S42 (b1 to b3 and c1 to c3) and Supplementary Video 2. For no blocking and PMMA blocking, the trajectory and speed of walking forward and backward is same and can be detected. However, for the case of blocking with PDMS+7 wt.% EG@MO-2, signal disappeared after the board inserted. This proves that the EG@MO-2 successfully absorbs radar waves and makes the target "stealth".

The S-parameters of 2-mm-thick EG@MO-2, PMMA, and graphite at 23-25 GHz were simulated using CST, as shown in Fig. S34. S11 denotes the reflection of

microwaves, and at 24 GHz, the S11 of graphite is 0.99, which is almost completely reflected, and the S11 of EG@MO-2 and PMMA is close to 0.5, which is partially reflected. S21 indicates the positive transmission of microwaves, and PMMA has an S21 of 0.85, indicating that it is almost transparent to microwaves, and graphite has an S21 close to 0 because it reflects microwaves almost completely. EG@MO-2, on the other hand, has an S21 of about 0.38, indicating a partial positive transmission. Using the absorption efficiency formula $A=1-S_{11}^2-S_{21}^2$, it can be concluded that the absorption efficiency of EG@MO-2 is about 67%, while both PMMA and graphite are close to 0. This indicates that although 24 GHz is not the best operating frequency for EG@MO-2, it still has some electromagnetic wave absorption capability.

Electromagnetic interference shielding efficiency of EG@MO Films

Electromagnetic interference shielding efficiency (EMI SE) serves as a metric that quantifies a material's capability to protect against electromagnetic interferences. The total EMI SE (EMI SE_T) is the sum of the contributions from reflection (SE_R), absorption (SE_A) and multiple internal reflections (SE_{MR}). The total SE_T can be written as:

$$SE_T = SE_R + SE_A + SE_{MR} \tag{24}$$

For calculations, SE_{MR} is generally considered negligible when SE_T is higher than 15 dB. SE_R and SE_A can be expressed in terms of reflection and absorption coefficient considering the power of the incident electromagnetic waves inside the shielding material as:

$$SE_R = 10\log\left(\frac{1}{1-R}\right) = 10\log\left(\frac{1}{1-|S_{11}|^2}\right) \tag{25}$$

$$SE_A = 10\log\left(\frac{1-R}{T}\right) = 10\log\left(\frac{1-|S_{11}|^2}{|S_{21}|^2}\right) \tag{26}$$

EG@MO was subjected to compression into a 0.1 mm film (named as EG@MO-x-F) at a pressure of 20 MPa, yielding ultra-lightweight characteristics (Figure S18). The graphite layer was uniformly compressed and sandwiched with MnO_2 nanowires (Figure S19), thereby enhancing the EMI shielding performance. The S-parameter of EG@MO-x-F was measured using the waveguide method, as shown in Fig. S31, and its SE was calculated. Figure S41(a) shows the SE_R in the range of 12.4 to 18 GHz, which was approximately 15 to 20 dB on average for all samples. However, there was a notable decline near 16 GHz. From the absorption efficiency SE_A (Figure S41(b)), the average of each sample was approximately 100 dB, rising to 108 dB at 16 GHz. The data demonstrated that the SEA was considerably larger than the SE_R, suggesting that the EG@MO predominantly functions as absorptive shielding. Additionally, an evident absorption peak was observed at 16 GHz. Figure S41(c) shows the total shielding efficiency (SE_T) of EG@MO. The shielding efficiency of EG was lower at 81 dB, while the shielding performances of different contents of EG@MO were similar, with EG@MO-3-F reaching 117 dB. In comparison to the existing literature on thin-film and multilayer EMI shielding materials (Figure S30), EG@MO-2-F exhibited superior EMI shielding capabilities. Furthermore, the SE of EG@MO-2-F at different thicknesses is shown in Figure S39. The correlation between SE_T and thickness was not significant, but as the thickness increases, SE_R gradually decreases while SE_A gradually increases.

It indicates that the absorption gradually increases as a percentage of the total shielding efficiency with the increase in the layers.

Supplementary Tables

Table S1 Details of EG@MO

	EG	Mn : C mole ratio	Treating
EG@MO-1-Pre	0.12 g	1 : 13.16	None
EG@MO-2-Pre	0.24 g	1 : 26.32	
EG@MO-3-Pre	0.36 g	1 : 39.48	
EG@MO-4-Pre	0.60 g	1 : 65.79	
EG@MO-1	0.12 g	1 : 13.16	Microwave
EG@MO-2	0.24 g	1 : 26.32	
EG@MO-3	0.36 g	1 : 39.48	
EG@MO-4	0.60 g	1 : 65.79	
EG@MO-1-Cov	0.12 g	1 : 13.16	Conventional
EG@MO-2-Cov	0.24 g	1 : 26.32	
EG@MO-3-Cov	0.36 g	1 : 39.48	
EG@MO-4-Cov	0.60 g	1 : 65.79	

Table S2 Mulliken atomic populations

Atom	Population					Charge(e)	Spin(/2)
	Spin	s	p	d	Total		
O #1	up:	0.97	2.57	0	3.54	-1.03	0.05
	dn:	0.97	2.52	0	3.49		
O #2	up:	0.97	2.57	0	3.54	-1.03	0.05
	dn:	0.97	2.52	0	3.49		
O #3	up:	0.97	2.57	0	3.54	-1.03	0.05
	dn:	0.97	2.52	0	3.49		
O #4	up:	0.97	2.57	0	3.54	-1.03	0.05
	dn:	0.97	2.52	0	3.49		
O #5	up:	0.97	2.57	0	3.54	-1.03	0.05
	dn:	0.97	2.52	0	3.49		
O #6	up:	0.97	2.57	0	3.54	-1.03	0.05
	dn:	0.97	2.52	0	3.49		
O #7	up:	0.97	2.57	0	3.54	-1.03	0.05
	dn:	0.97	2.52	0	3.49		
O #8	up:	0.97	2.57	0	3.54	-1.03	0.05
	dn:	0.97	2.52	0	3.49		
O #9	up:	0.98	2.45	0	3.43	-0.92	-0.06
	dn:	0.98	2.51	0	3.49		
O #10	up:	0.98	2.45	0	3.43	-0.92	-0.06
	dn:	0.98	2.51	0	3.49		
O #11	up:	0.98	2.45	0	3.43	-0.92	-0.06
	dn:	0.98	2.51	0	3.49		
O #12	up:	0.98	2.45	0	3.43	-0.92	-0.06
	dn:	0.98	2.51	0	3.49		
O #13	up:	0.98	2.45	0	3.43	-0.92	-0.06
	dn:	0.98	2.51	0	3.49		
O #14	up:	0.98	2.45	0	3.43	-0.92	-0.06
	dn:	0.98	2.51	0	3.49		
O #15	up:	0.98	2.45	0	3.43	-0.92	-0.06
	dn:	0.98	2.51	0	3.49		
O #16	up:	0.98	2.45	0	3.43	-0.92	-0.06
	dn:	0.98	2.51	0	3.49		
Mn #1	up:	1	3	4.03	8.03	1.95	3.02
	dn:	1	3	1.02	5.02		
Mn #2	up:	1	3	4.03	8.03	1.95	3.02
	dn:	1	3	1.02	5.02		
Mn #3	up:	1	3	4.03	8.03	1.95	3.02
	dn:	1	3	1.02	5.02		
Mn #4	up:	1	3	4.03	8.03	1.95	3.02
	dn:	1	3	1.02	5.02		
Mn #5	up:	1	3	4.04	8.04	1.95	3.02

	dn:	1	3	1.02	5.02		
Mn #6	up:	1	3	4.04	8.04	1.95	3.02
	dn:	1	3	1.02	5.02		
Mn #7	up:	1	3	4.04	8.04	1.95	3.02
	dn:	1	3	1.02	5.02		
Mn #8	up:	1	3	4.04	8.04	1.95	3.02
	dn:	1	3	1.02	5.02		

Table S3 Mulliken bond populations

Bond	Population	Spin	Length (A)
O 7 - Mn 4	0.10	-0.09	1.87817
O 3 - Mn 6	0.10	-0.09	1.87817
O 8 - Mn 3	0.10	-0.09	1.87817
O 4 - Mn 5	0.10	-0.09	1.87817
O 6 - Mn 1	0.10	-0.09	1.87817
O 5 - Mn 2	0.10	-0.09	1.87817
O 2 - Mn 7	0.10	-0.09	1.87817
O 1 - Mn 8	0.10	-0.09	1.87817
O 2 - Mn 4	0.04	-0.04	1.88678
O 6 - Mn 8	0.04	-0.04	1.88678
O 5 - Mn 7	0.04	-0.04	1.88678
O 1 - Mn 3	0.04	-0.04	1.88678
O 8 - Mn 6	0.04	-0.04	1.88678
O 7 - Mn 5	0.04	-0.04	1.88678
O 4 - Mn 2	0.04	-0.04	1.88678
O 3 - Mn 1	0.04	-0.04	1.88678
O 16 -- Mn 8	0.10	-0.07	1.90113
O 13 -- Mn 5	0.10	-0.07	1.90113
O 11 -- Mn 3	0.10	-0.07	1.90113
O 10 -- Mn 2	0.10	-0.07	1.90113
O 15 -- Mn 7	0.10	-0.07	1.90113
O 9 - Mn 1	0.10	-0.07	1.90113
O 14 -- Mn 6	0.10	-0.07	1.90113
O 12 -- Mn 4	0.10	-0.07	1.90113
O 10 -- Mn 3	0.04	-0.04	1.90661
O 14 -- Mn 7	0.04	-0.04	1.90661
O 13 -- Mn 8	0.04	-0.04	1.90661
O 16 -- Mn 5	0.04	-0.04	1.90661
O 12 -- Mn 1	0.04	-0.04	1.90661
O 11 -- Mn 2	0.04	-0.04	1.90661
O 9 - Mn 4	0.04	-0.04	1.90661
O 15 -- Mn 6	0.04	-0.04	1.90661

* The bond that population < 0 not showed here

Table S4. Resistivity and conductivity of EG@MO-2 at various concentrations

Concentration	Run	Resistivity (Ω·m)	Conductivity (S/m)
3 wt.%	-	$\geq 10^{10}$ *	$\leq 10^{-10}$ *
5 wt.%	1	4.33×10^{9}	2.31×10^{-10}
	2	6.50×10^{9}	1.54×10^{-10}
	3	5.72×10^{9}	1.75×10^{-10}
7 wt.%	1	4.20×10^{9}	2.38×10^{-10}
	2	5.44×10^{9}	1.84×10^{-10}
	3	6.33×10^{9}	1.58×10^{-10}
9 wt.%	1	1.25×10^{1}	8.00×10^{-2}
	2	1.49×10^{1}	6.71×10^{-2}
	3	1.36×10^{1}	7.35×10^{-2}
11 wt.%	1	1.10×10^{1}	9.09×10^{-2}
	2	1.55×10^{1}	6.45×10^{-2}
	3	1.24×10^{1}	8.06×10^{-2}

* Out of range

Supplementary Figures

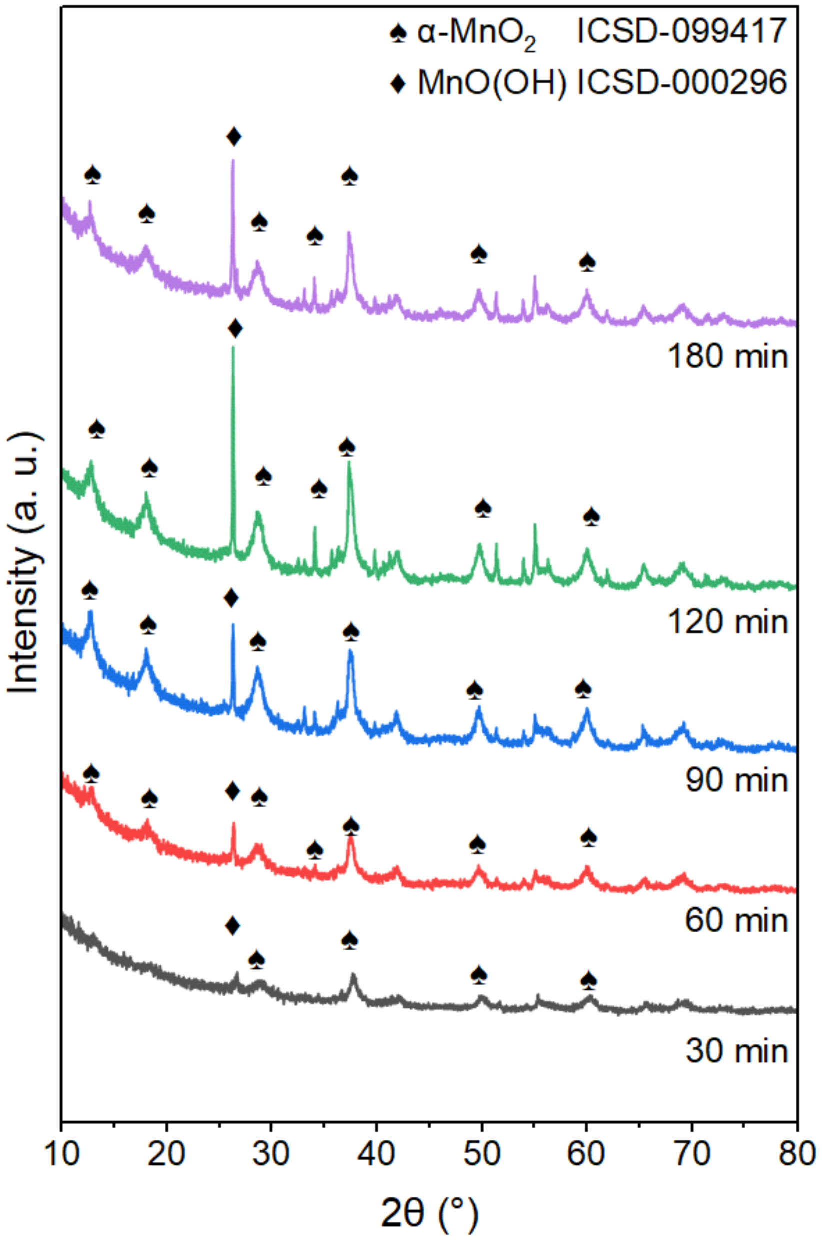

Figure S1. Reaction time of microwave hydrothermal at 180°C

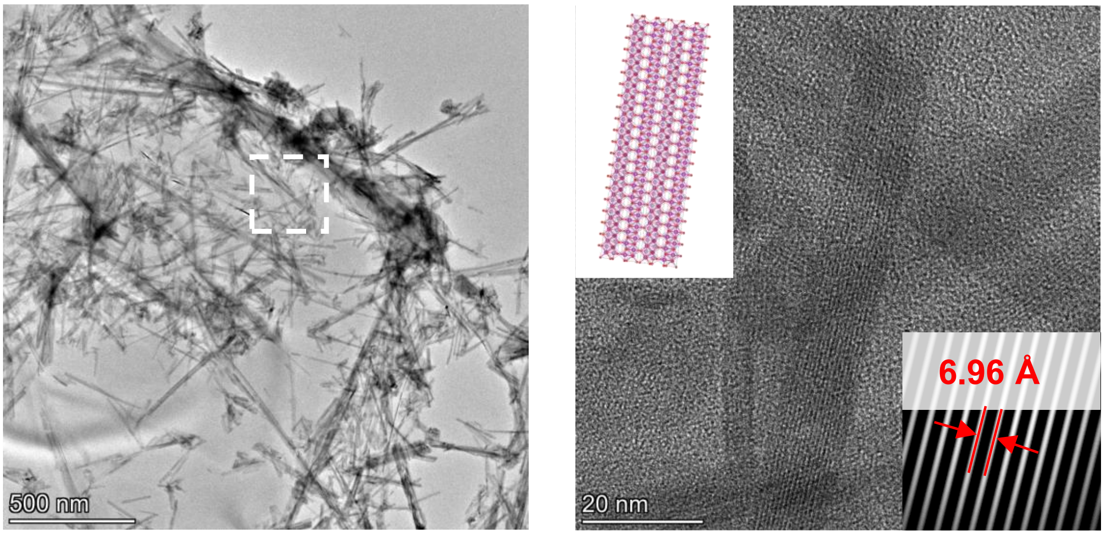

Figure S2. TEM image of EG@MO-2

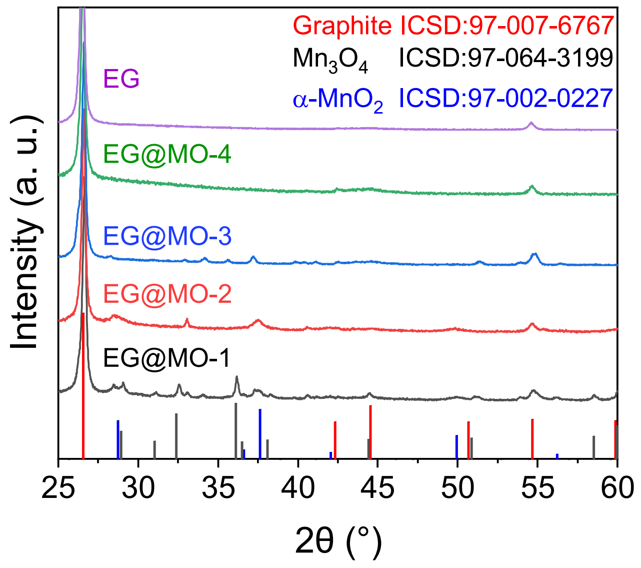

Figure S3. XRD pattern of EG and EG@MOs.

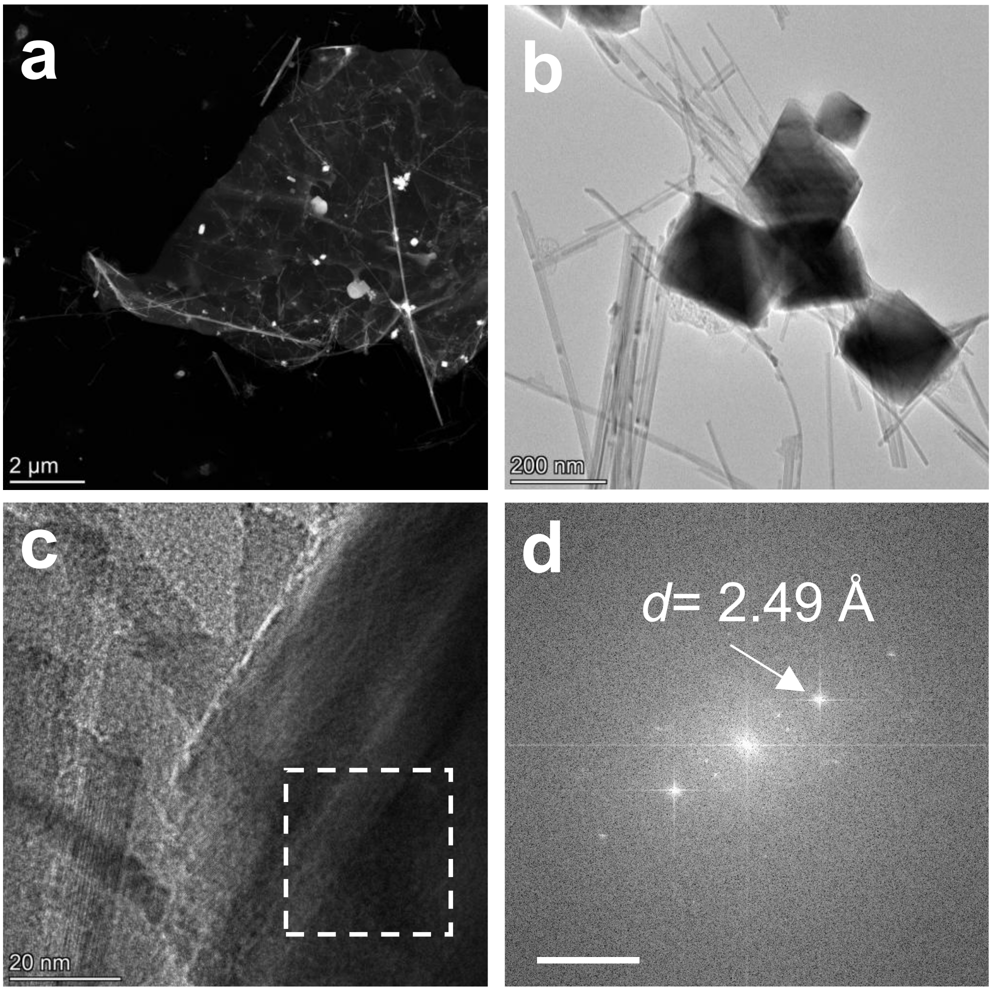

Figure S4. TEM image and FFT pattern of EG@MO-1

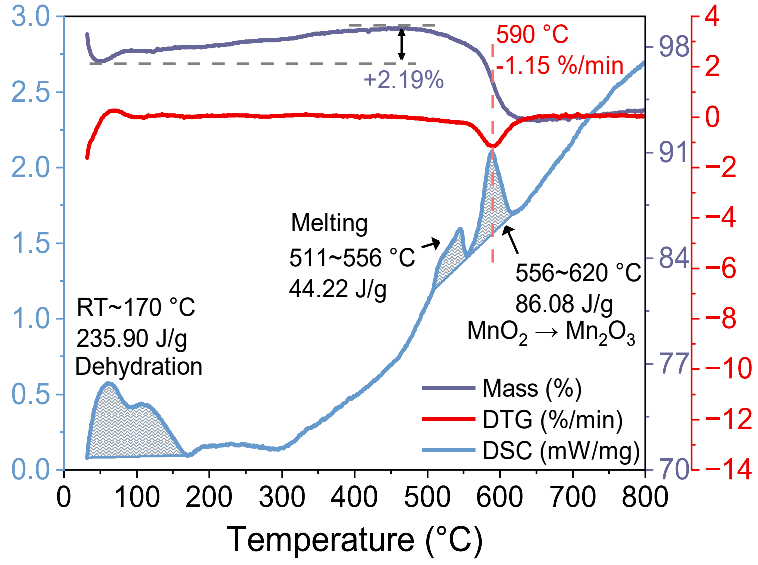

Figure S5. TG-DSC curve of MO in the range of RT~800 °C (10 °C/min)

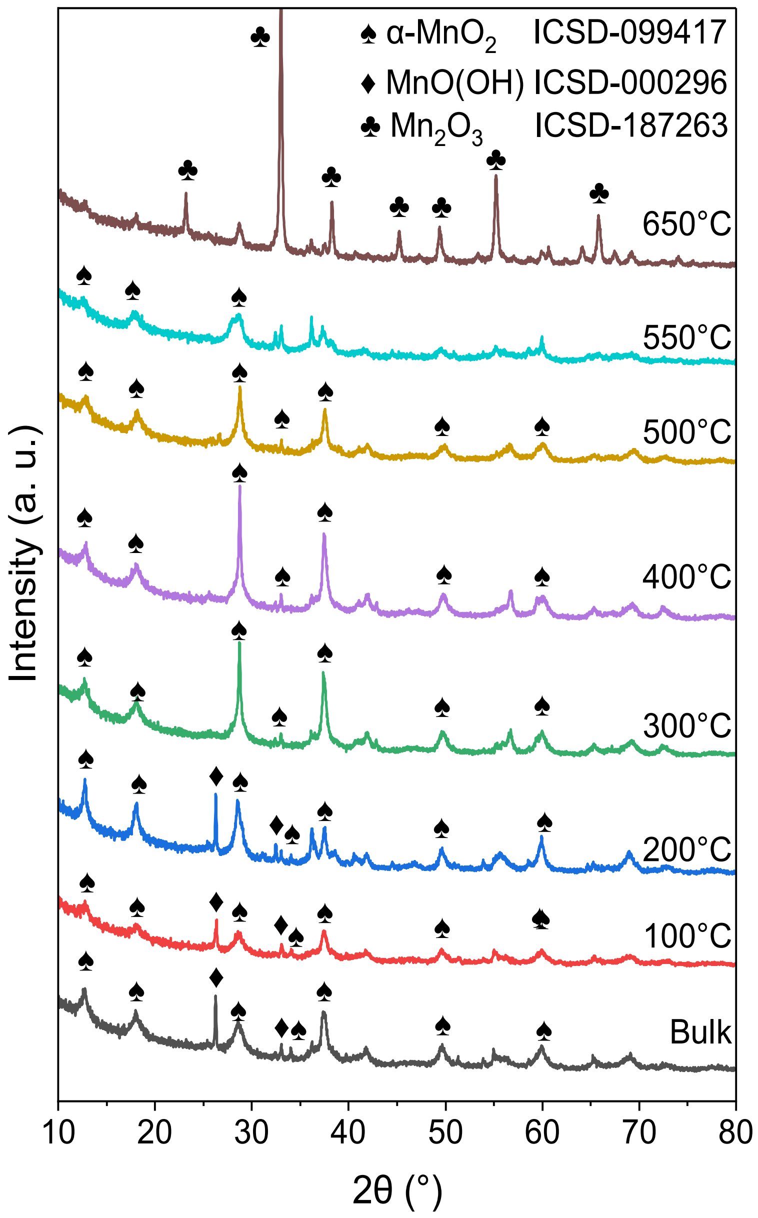

Figure S6. XRD patterns for the MO roasting at RT~650°C for 10 min each

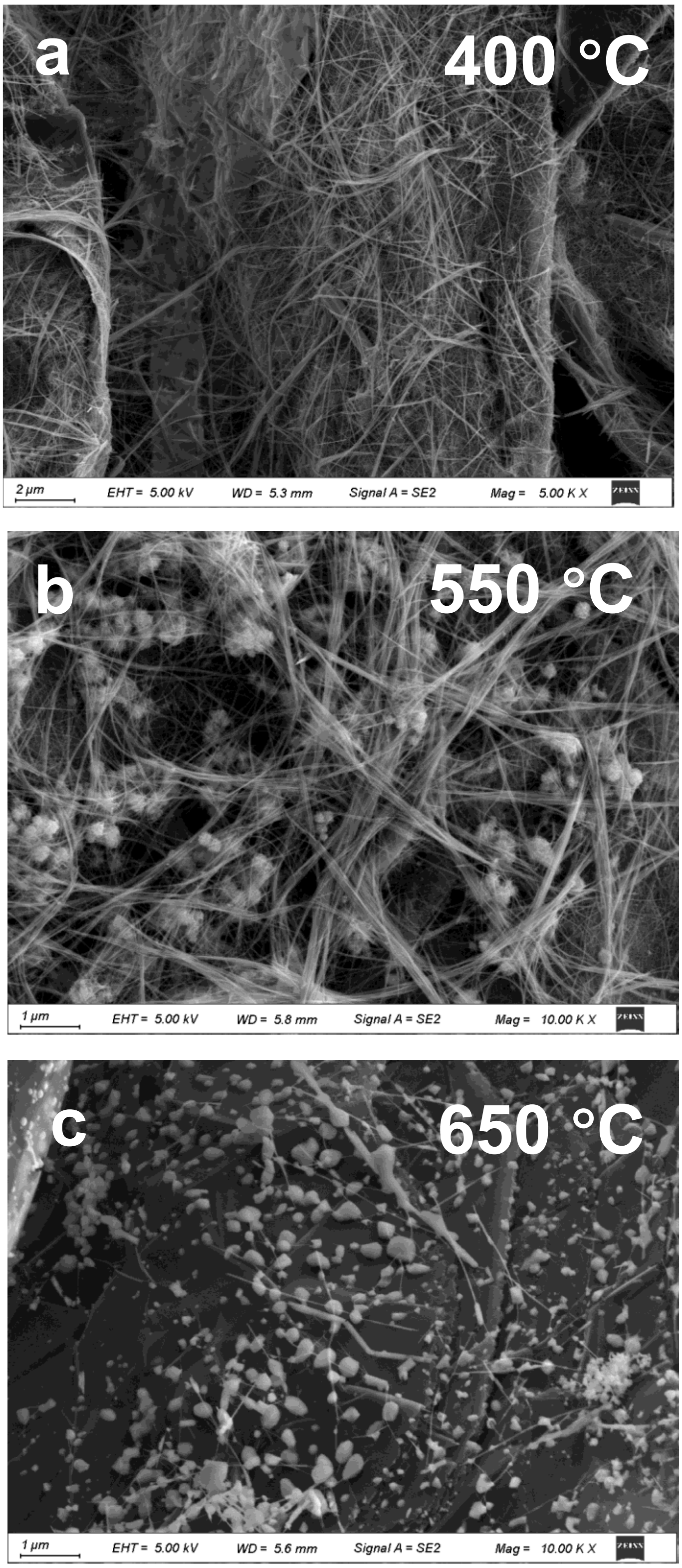

Figure S7. SEM images of the EG@MO treated under various temperature

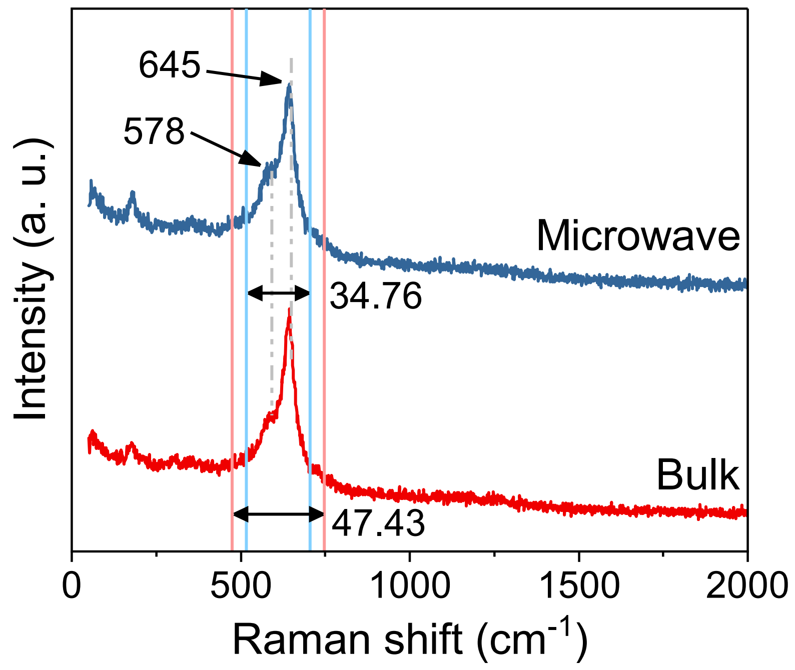

Figure S8. Raman shift of MO-Pre before and after microwave

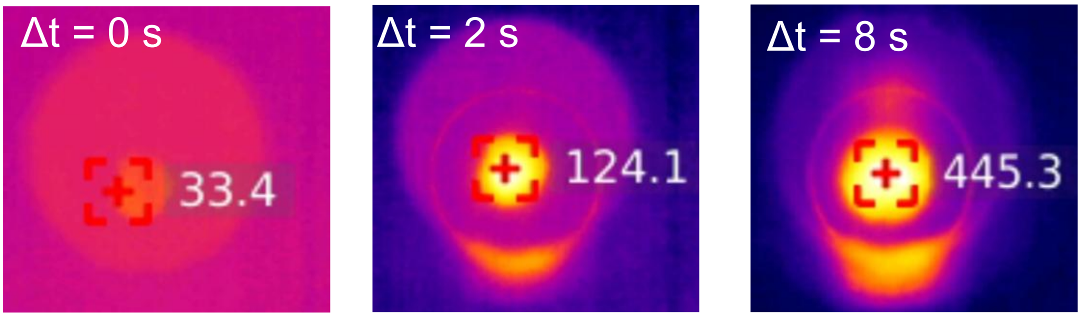

Figure S9 Infrared thermography of EG@MO under 400W of microwave

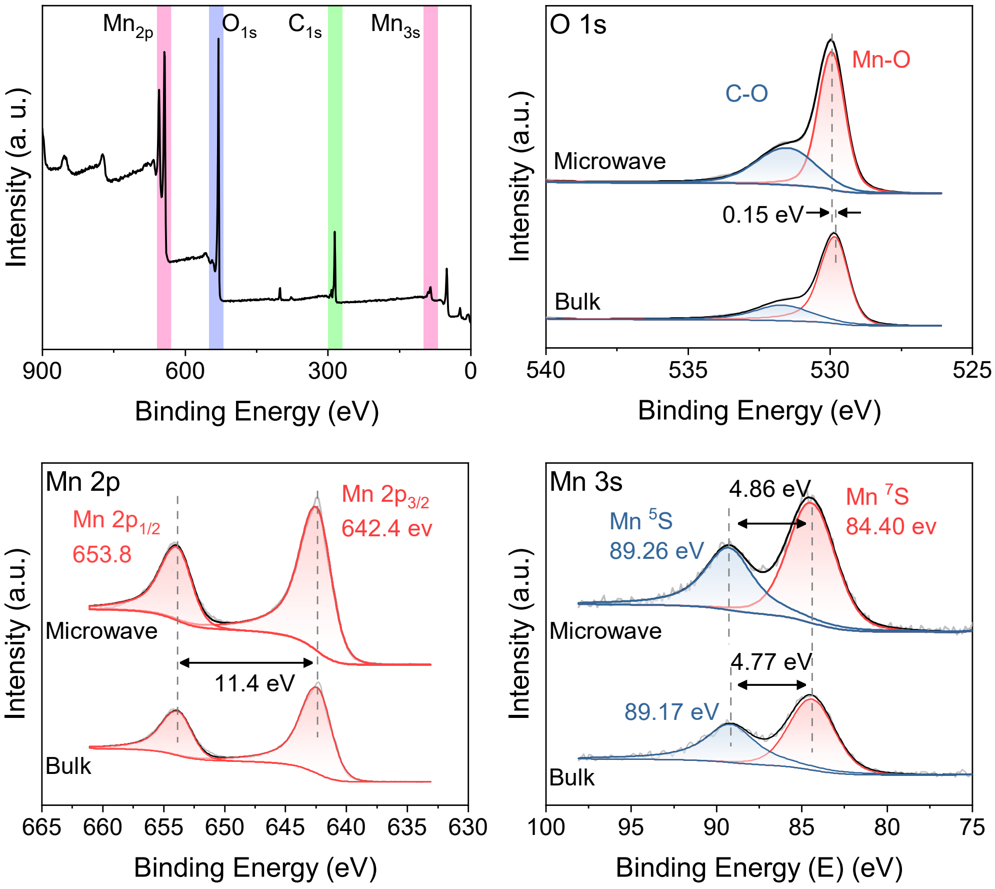

Figure S10. XPS spectra of Mn_{2p}, Mn_{3s}, and O_{1s} of MO before and after microwave

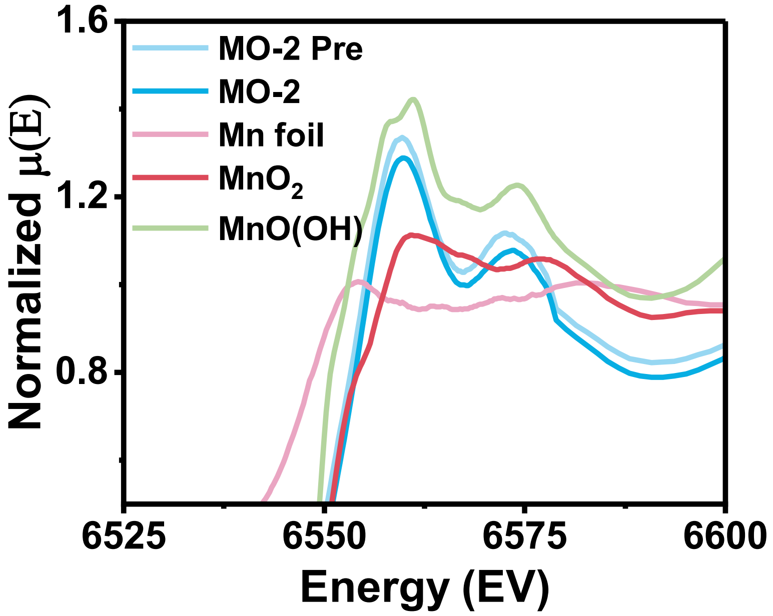

Figure S11 White line of the XANES

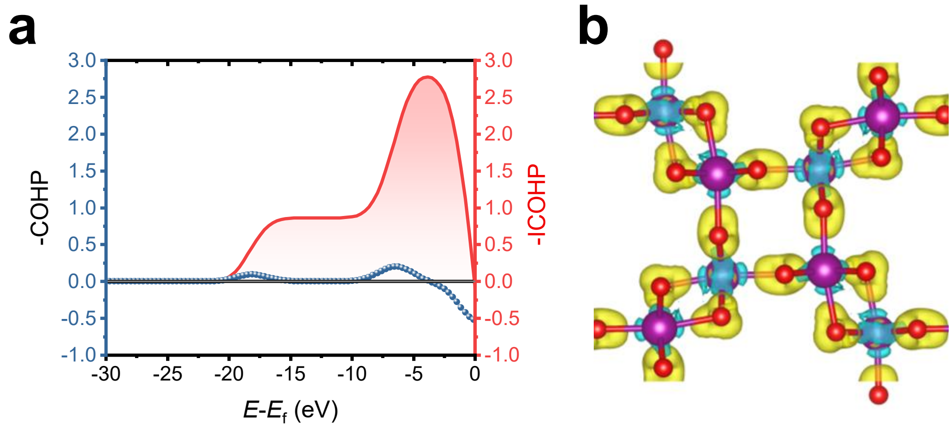

Figure S12. (a)COHP and ICOHP, (b) electron density difference of α-MnO2

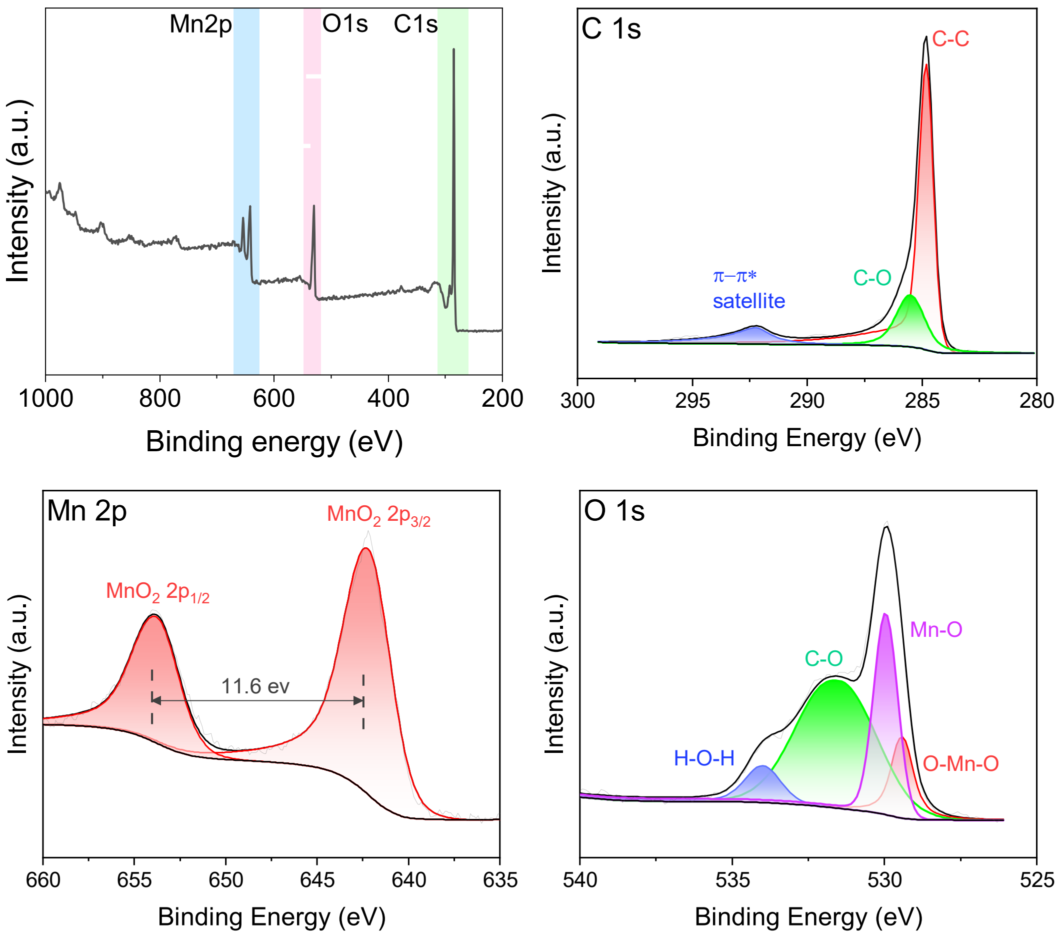

Figure S13. XPS spectra for EG@MO-2

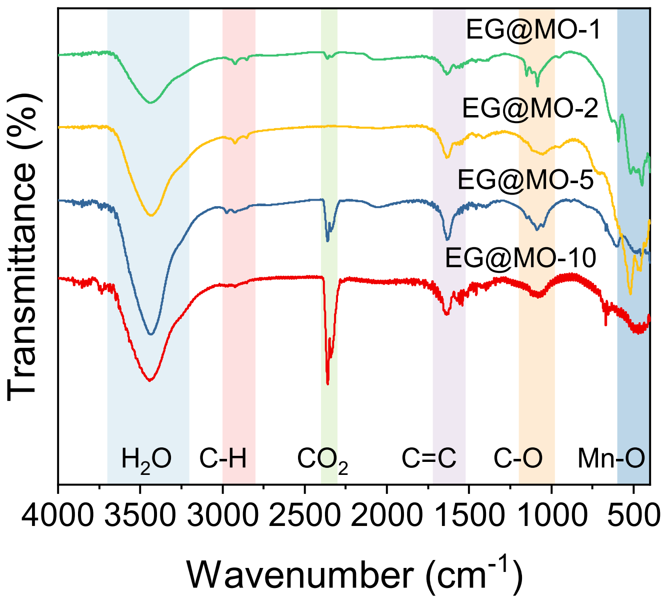

Figure S14 FT-IR spectra for EG@MO-1 to 4

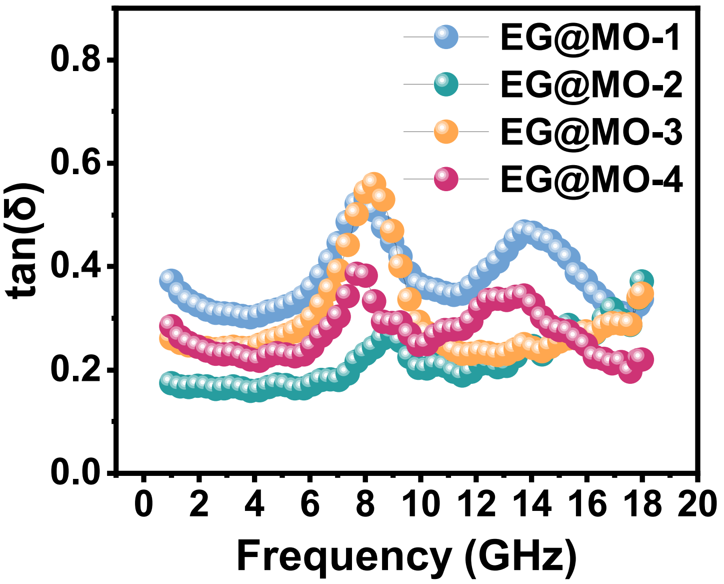

Figure S 15 loss tangent of permittivity

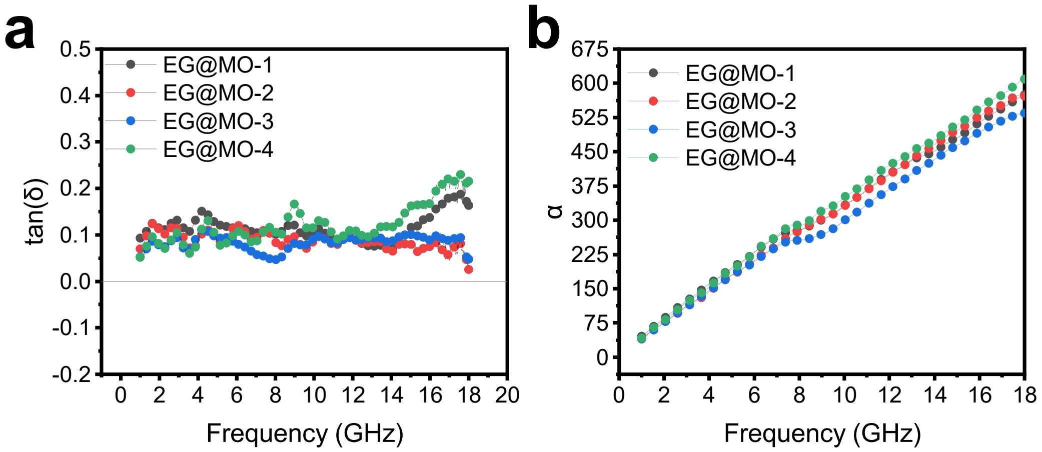

Figure S16 (a) loss tangent of permeability (b) loss factor of EG@MO 1 to 4

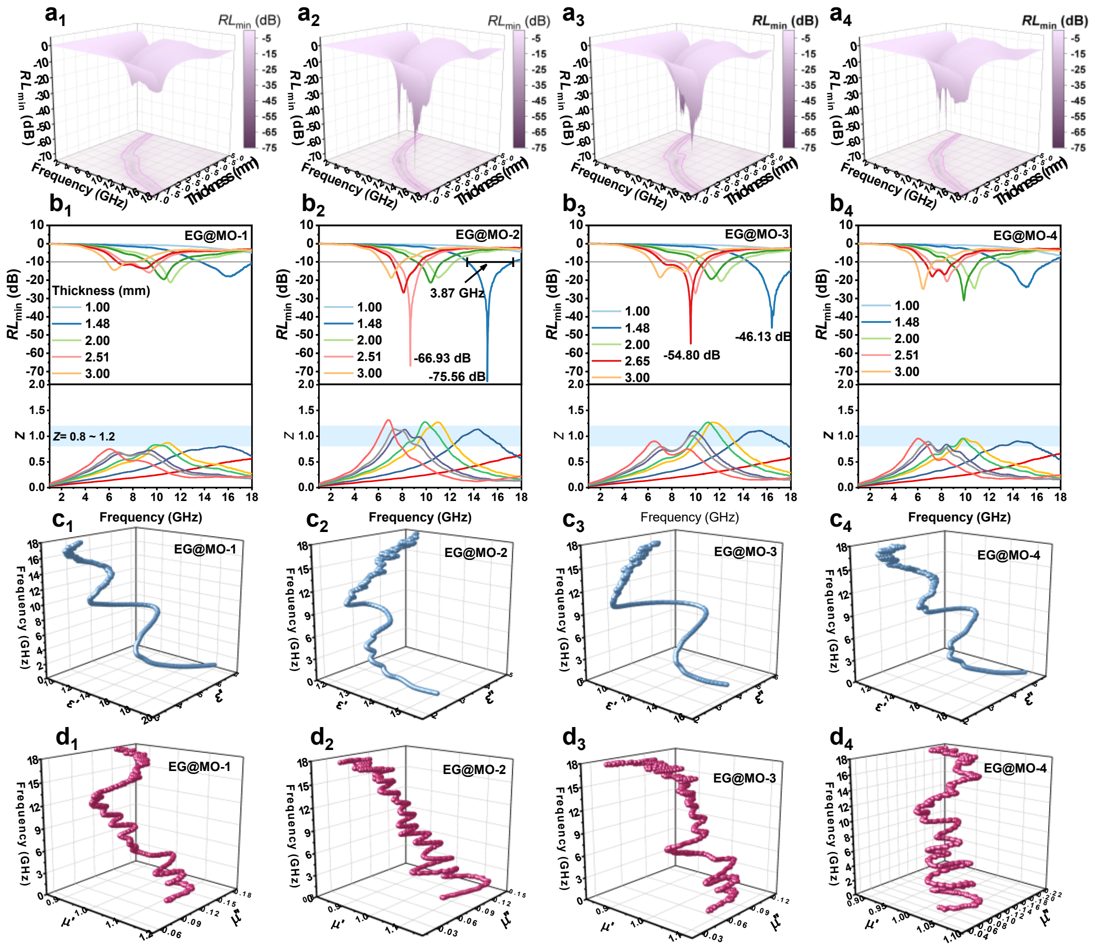

Figure S17 EM absorptions of EG@MO. (a1 – a4) RL as a function of thickness and frequency of EG@MO-1 to 4, (b1 – b4) RL_{min} at various thicknesses and impendence matching Z of EG@MO-1 to 4, (c1 – c4) dielectric and (d1-d4) magnetic Cole-Cole plot of EG@MO-1 to 4

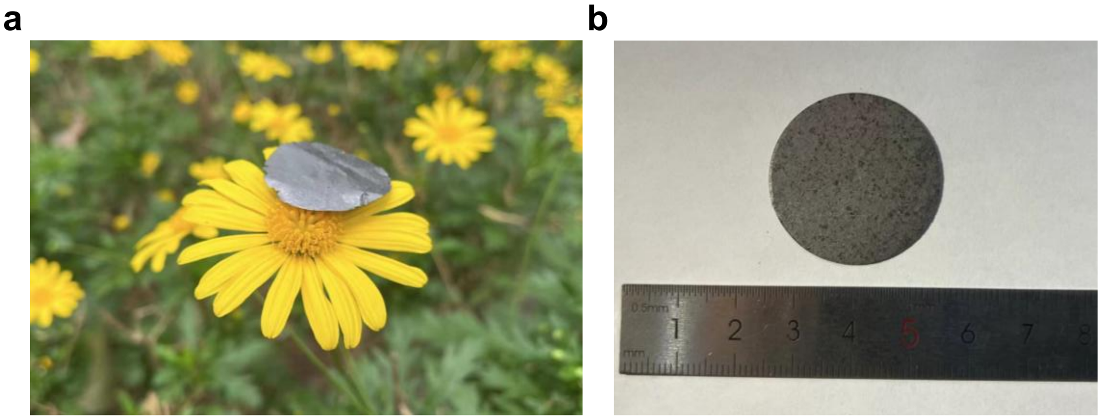

Figure S18. EG@MO Film (a) sitting on the paddle of a flower and (b) with a diameter of 30mm.

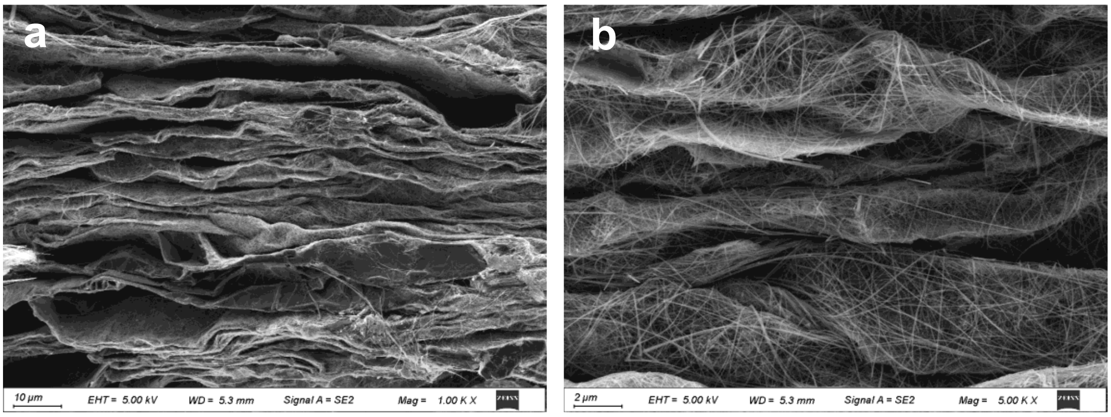

Figure S19. SEM image of EG@MO Film

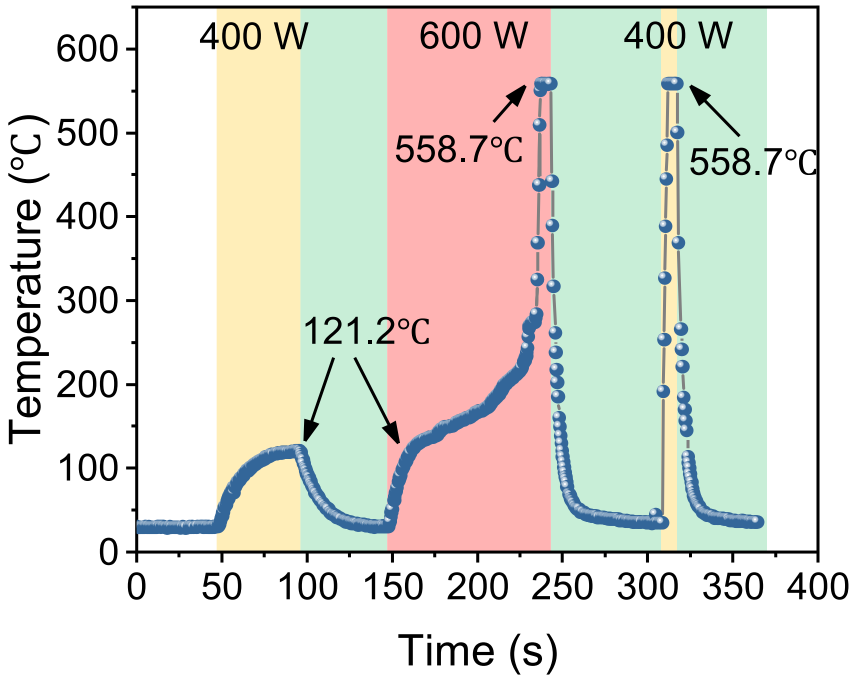

Figure S20. Temperature as a function of time for EG@MO-Pre heating in three circles at 400W, 600W and 400W of microwave

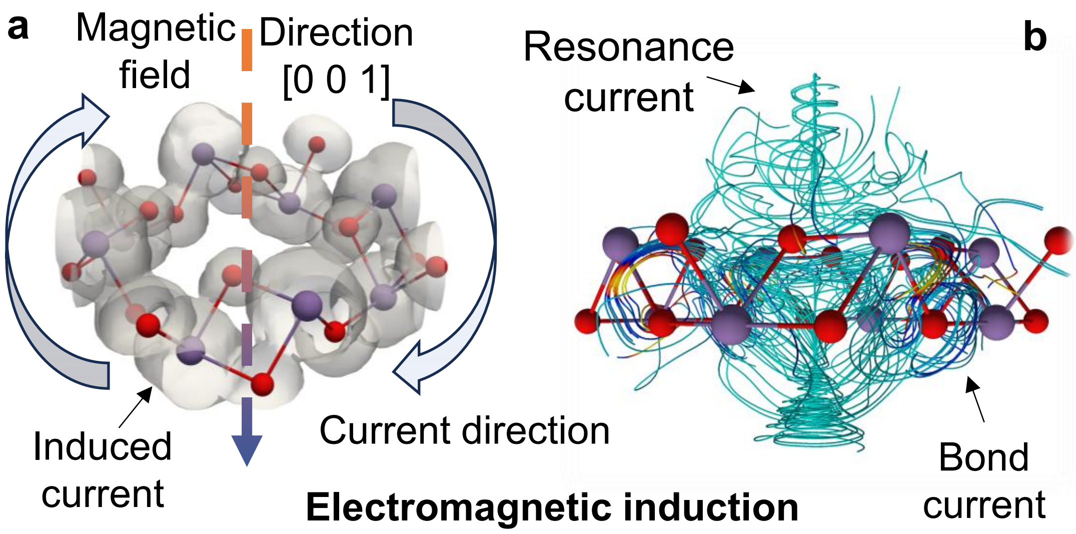

Figure S21. magnetically induced currents of α-MnO2

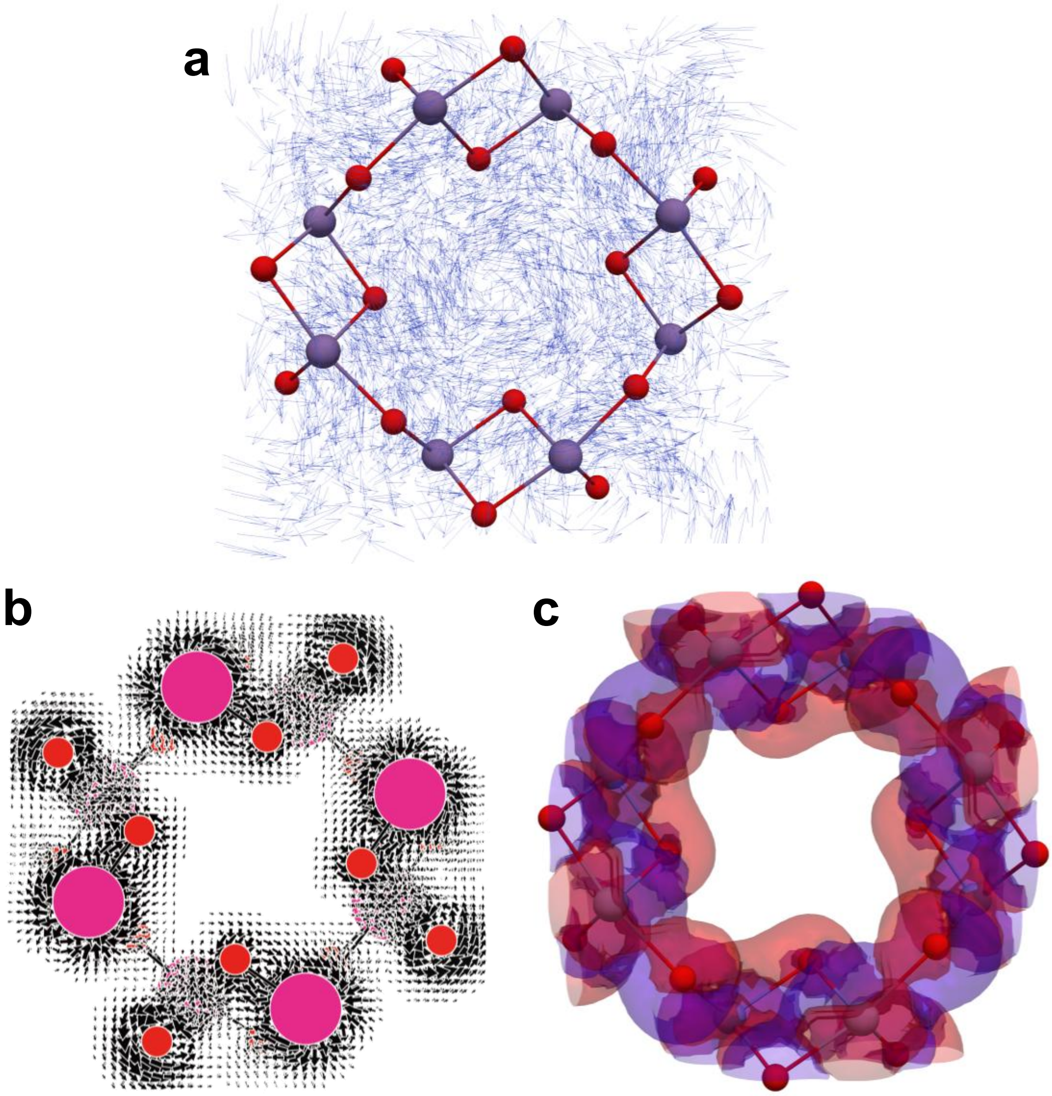

Figure S22 (a and b) magnetically induced currents and (c) Paratropic (red) and diatropic (blue) magnetically induced currents.

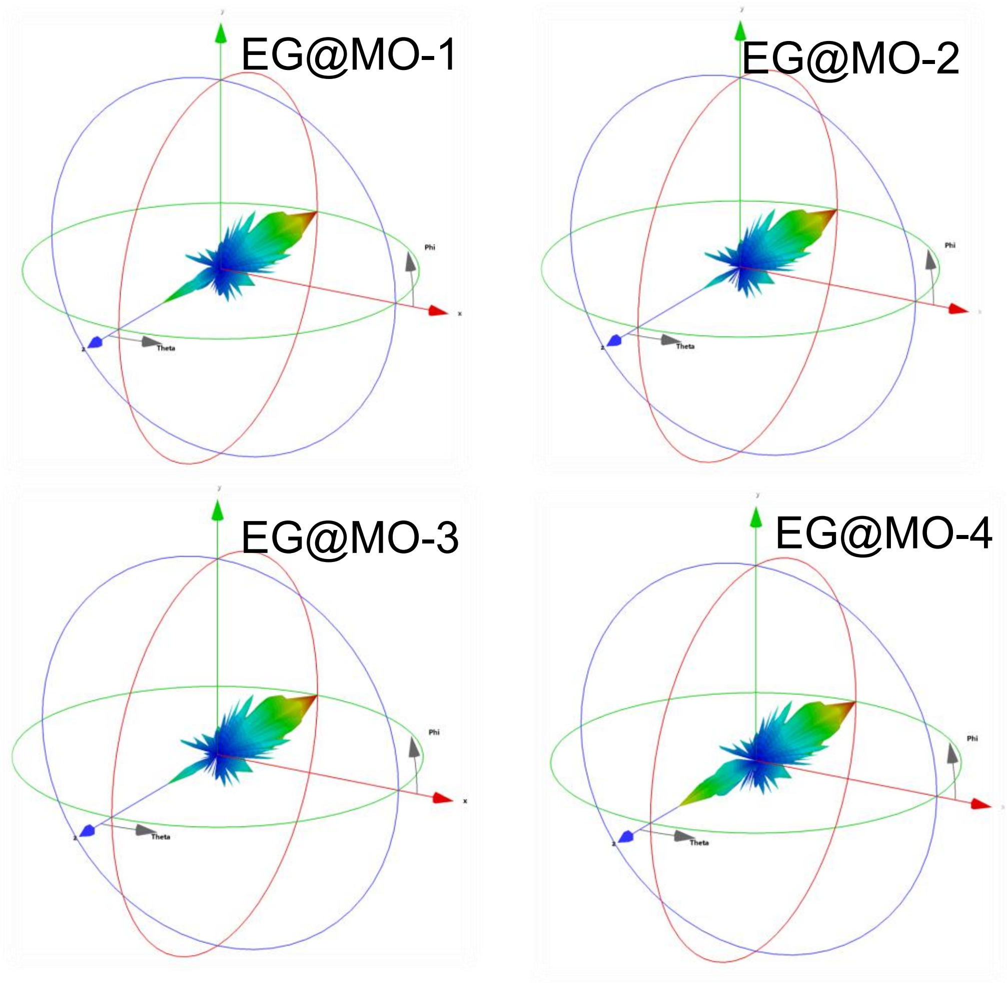

Figure S23. The far-field reflections of EG@MOs.

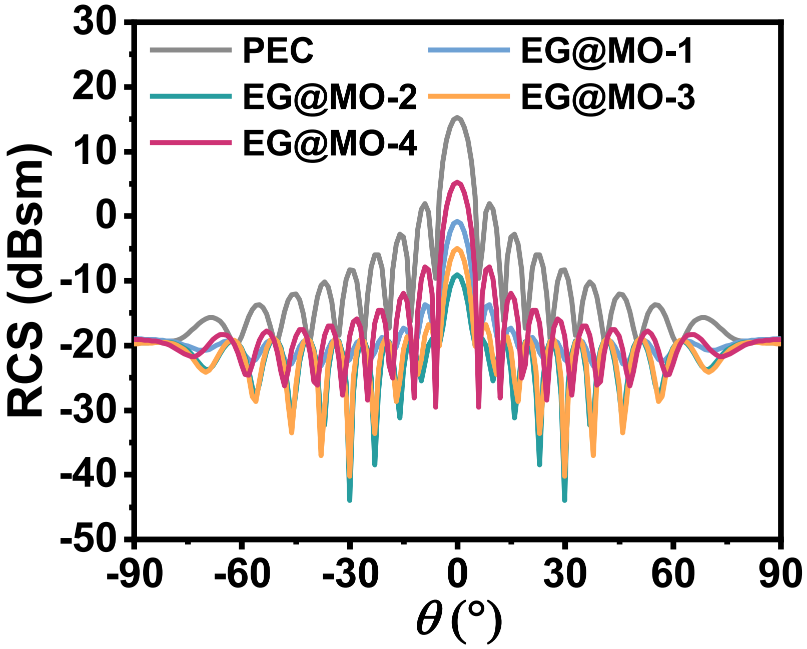

Figure S24. the RCS of EG@MOs as a function of θ.

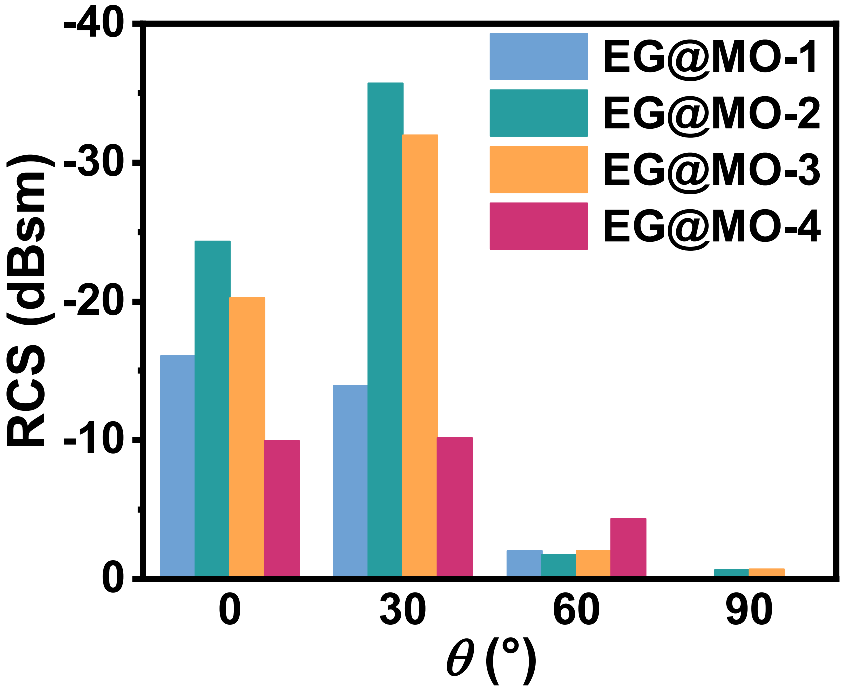

Figure S25. the RCS losses of EG@MOs as a function of θ.

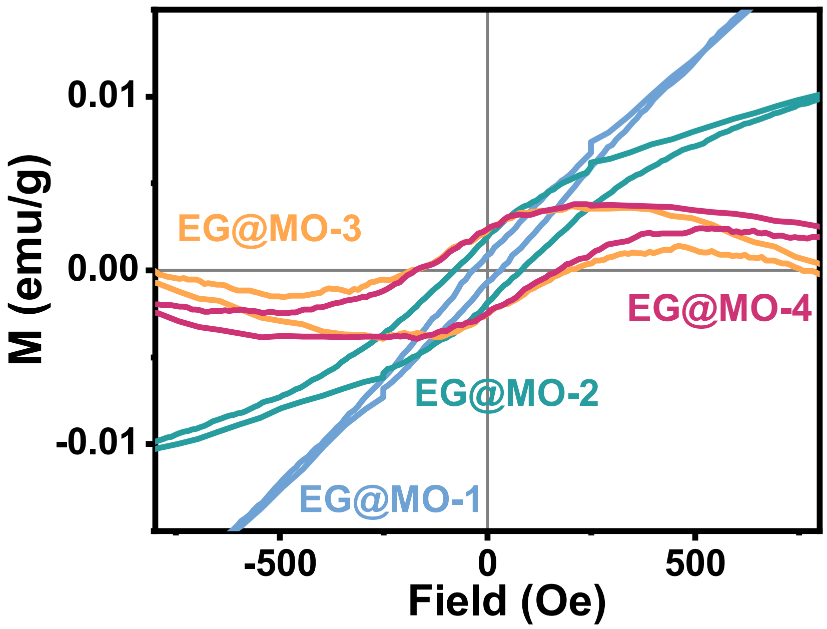

Figure S26 M-H curve in -1k~+1k Oe

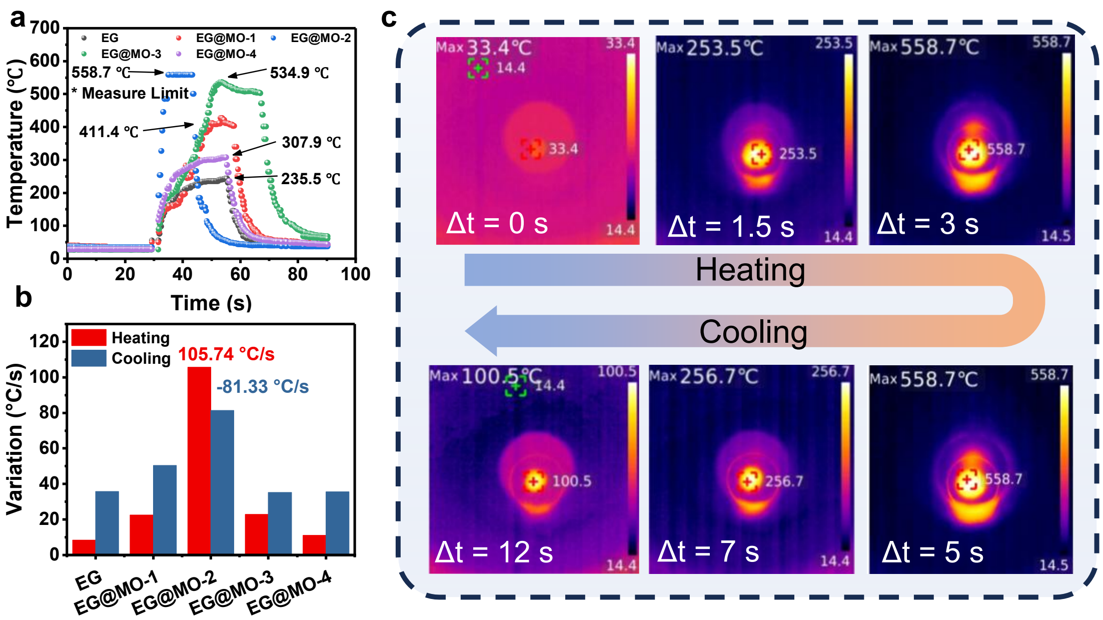

Figure S27 (a) Temperature as a function of time, (b) temperature variation, (c) IR thermography of EG@MOs at 400W of microwave.

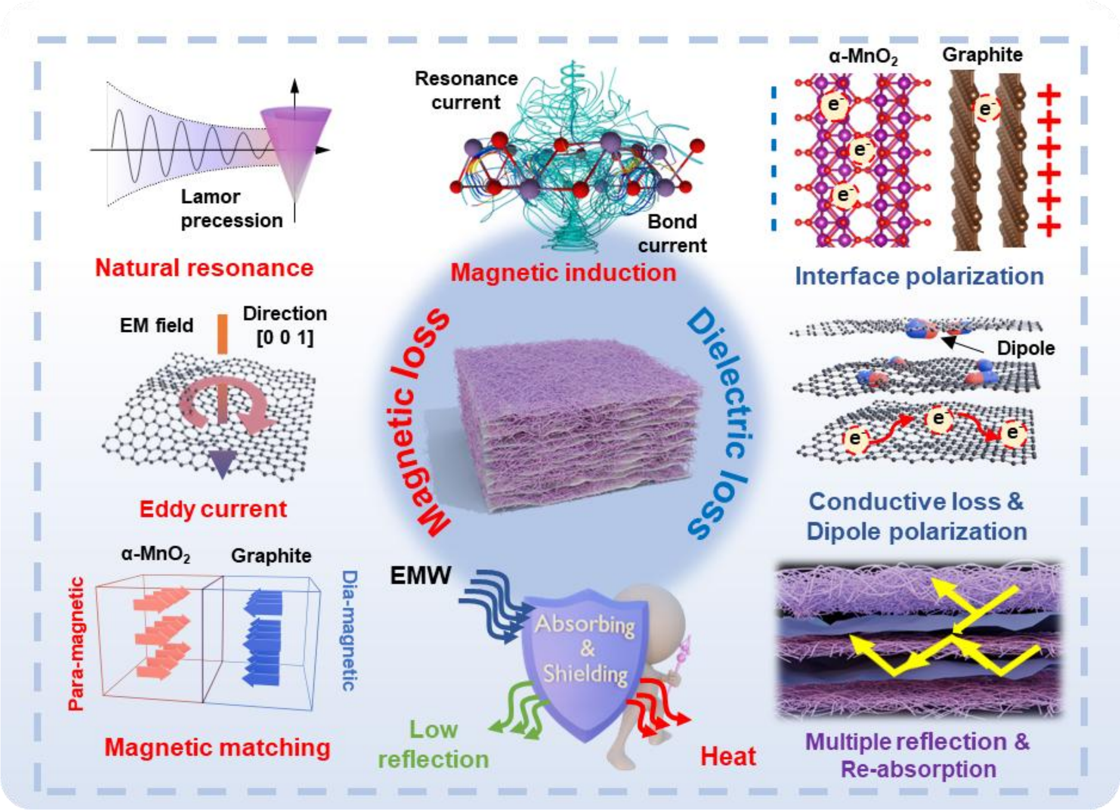

Figure S28 Schematic diagram of the electromagnetic wave absorption mechanism

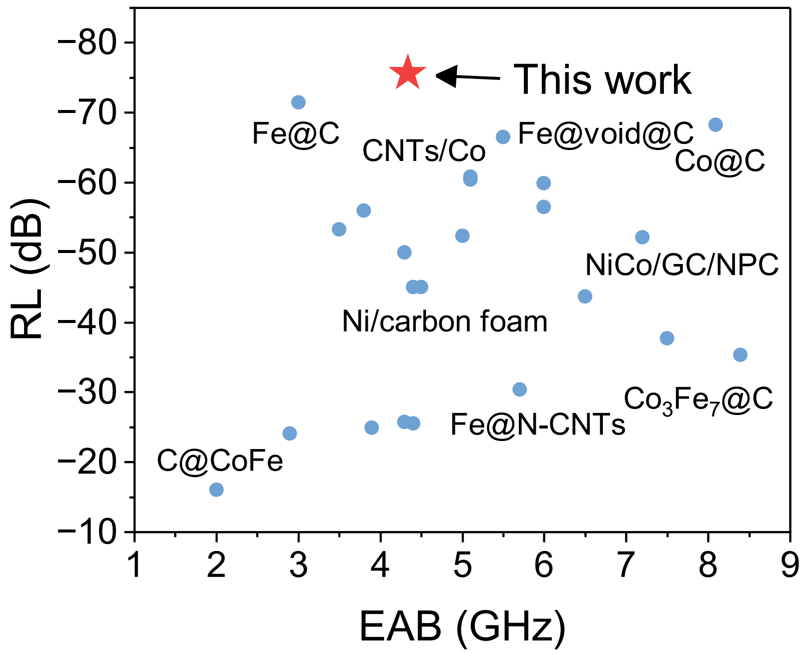

Figure S29 Comparison with recently developed carbon/magnetic metal composites[2–22]

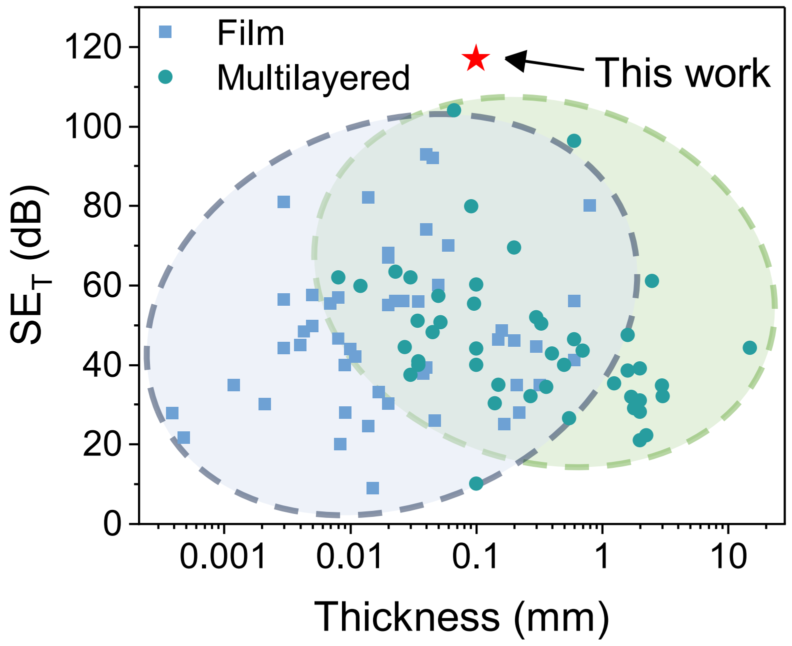

Figure S30. Comparison with recently developed film type[23–69] and multilayered type[70–114] EMI shielding materials

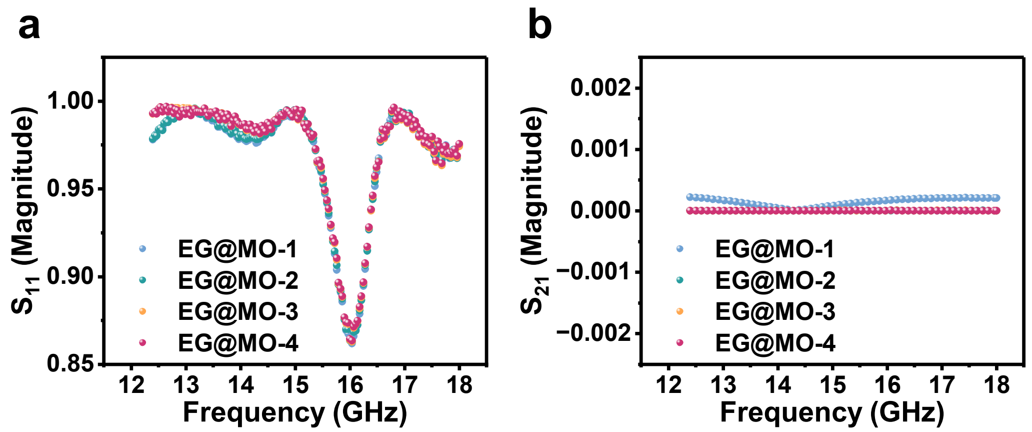

Figure S31. S-Parameters for EG@MO films.

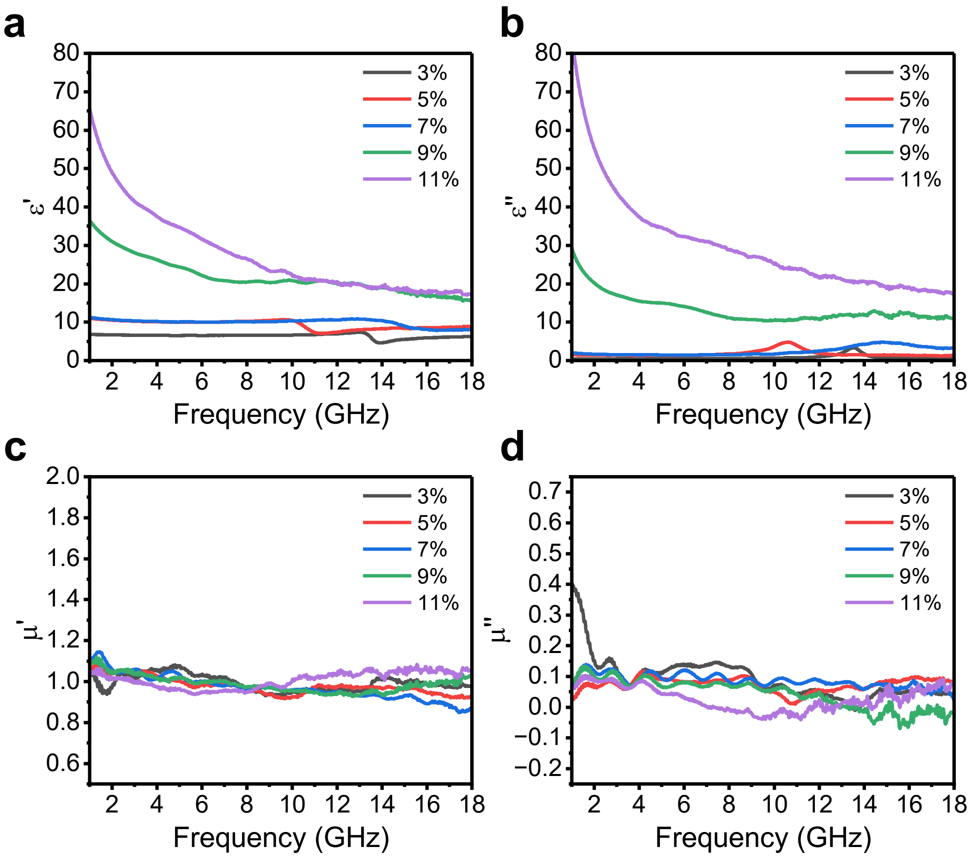

Figure S32. Electromagnetic properties (a) ε’, (b) ε’’, (c) μ’ and (d) μ’’ of EG@MO-2 at various concentrations.

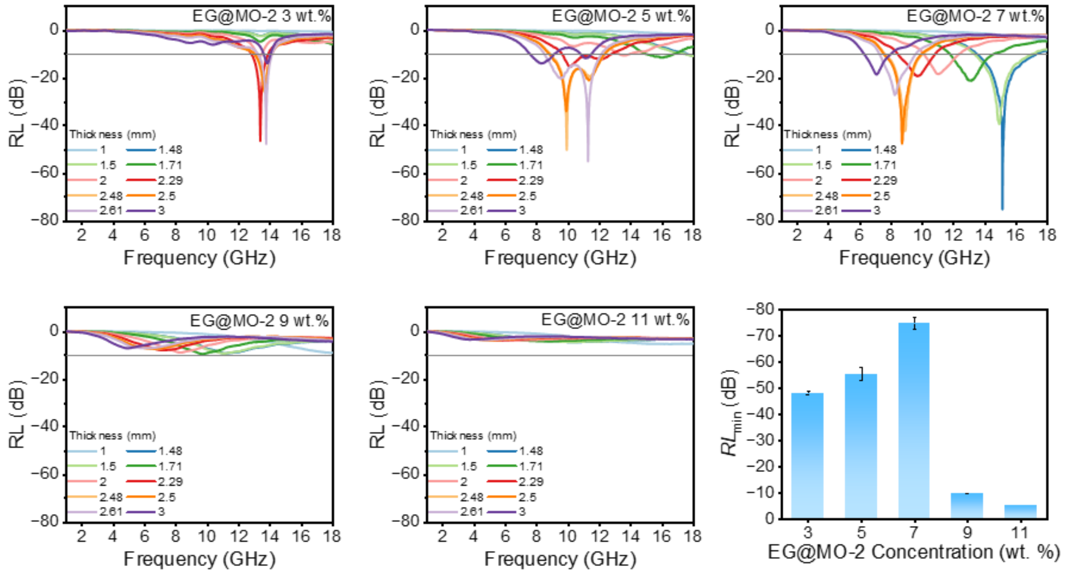

Figure S33. Reflection loss of EG@MO-2 at various concentrations.

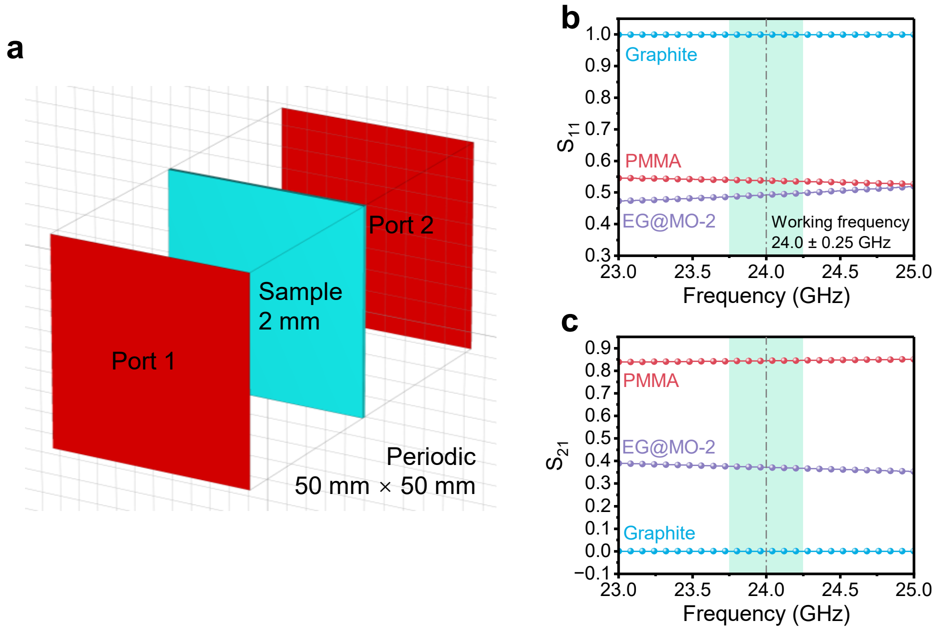

Figure S34. (a) Schematic diagram of the simulation of 7 wt.% EG@MO-2 with PMMA at 2 mm thickness; Simulated S-Parameters (b) S_{11} and (c) S_{21} of EG@MO-2, Graphite and PMMA at around 24 GHz.

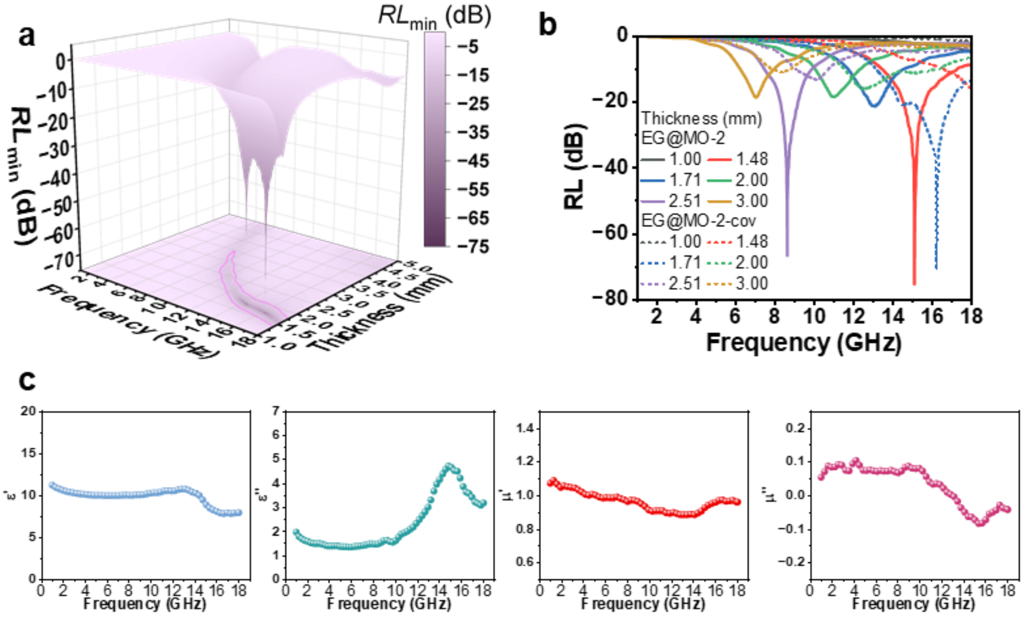

Figure S35. (a) Reflection loss, (b) comparison between EG@MO-2 and EG@MO-2-cov (c) electromagnetic parameters of EG@MO-2-cov.

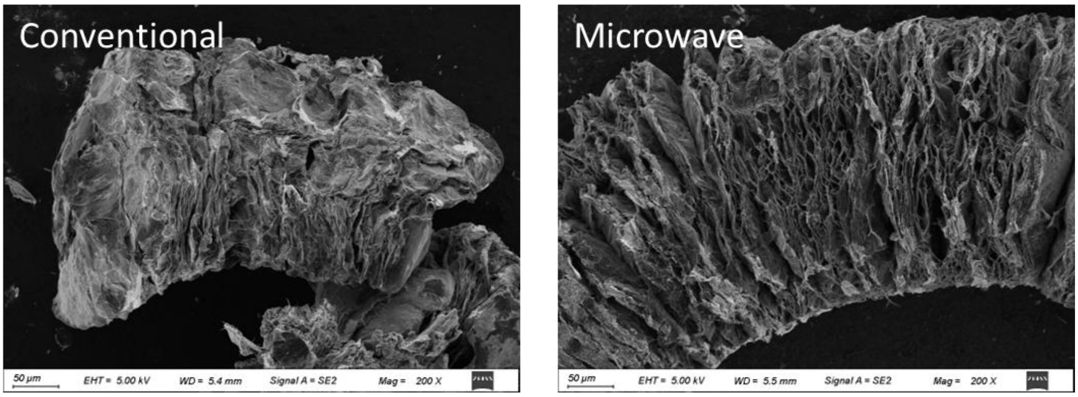

Figure S36. SEM image of EG@MO-2-cov and EG@MO-2.

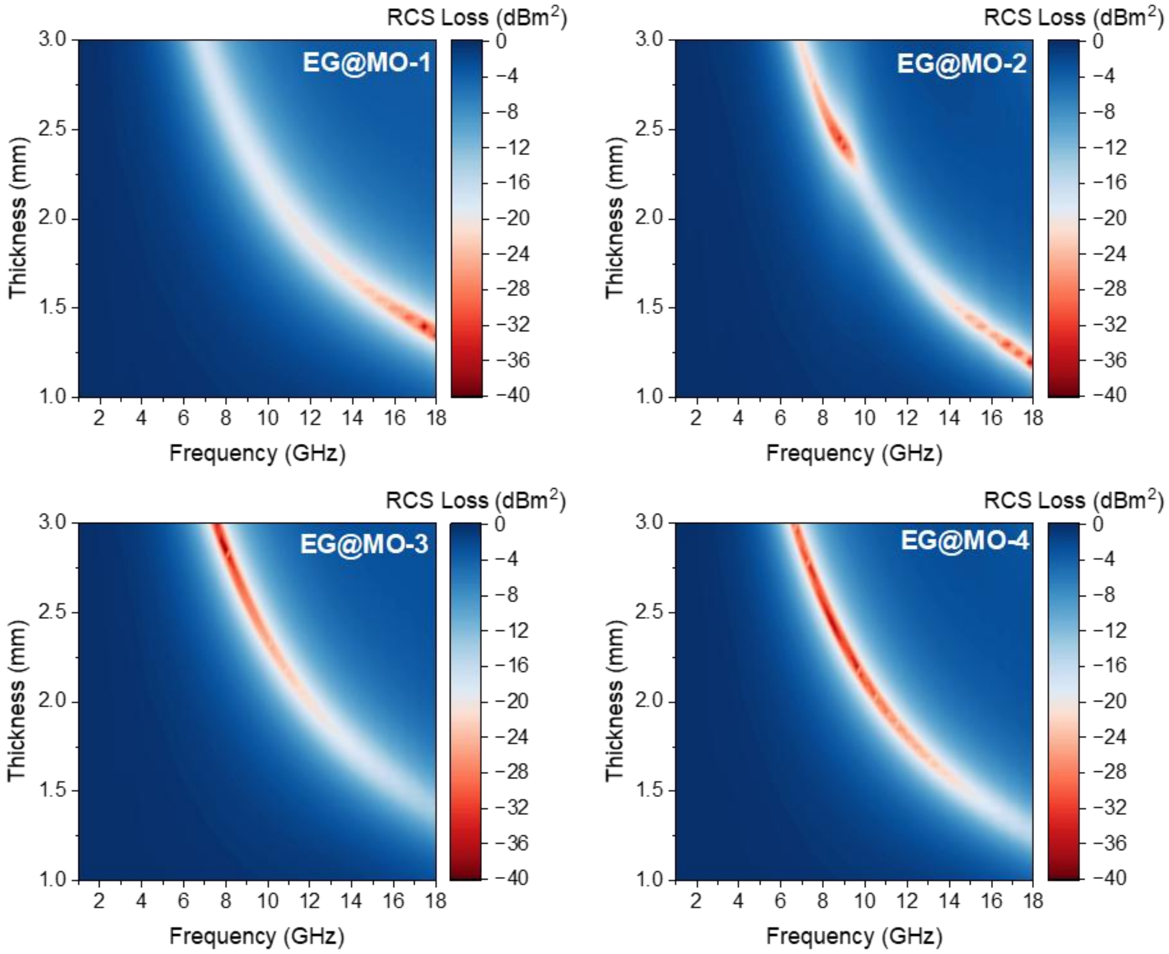

Figure S37. RCS loss distribution as a function of frequency and thickness.

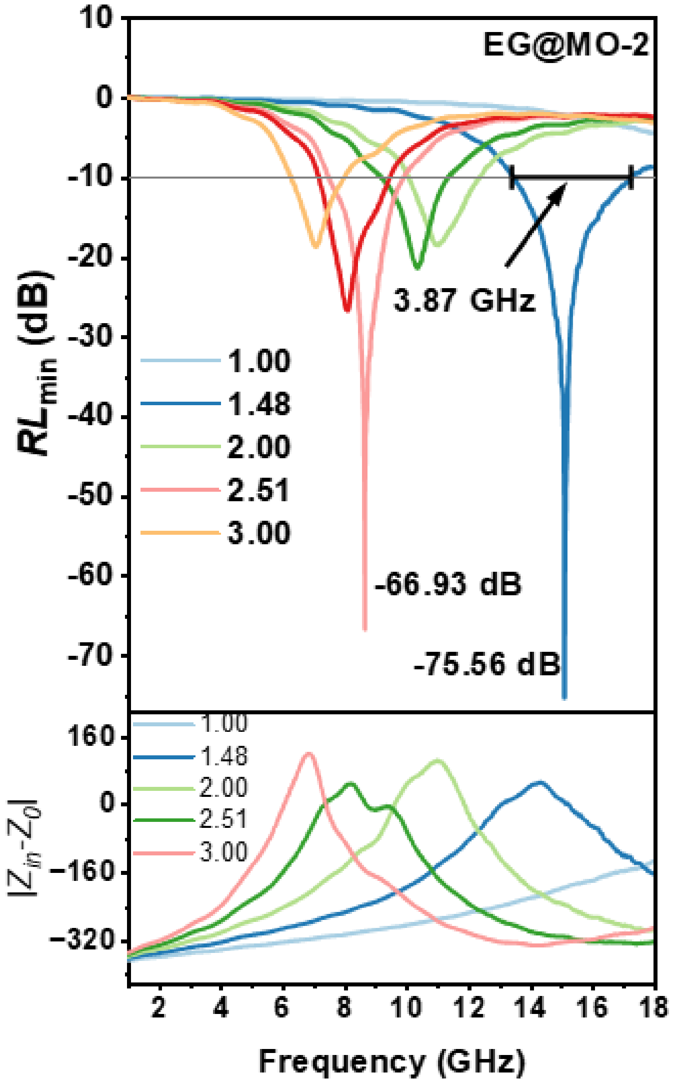

Figure S38. Reflection loss and impedance matching $|Z_{in}-Z_0|$

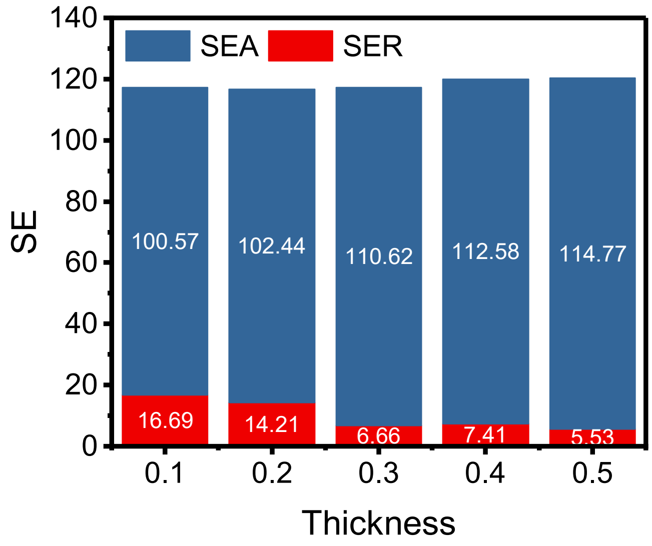

Figure S39 Average SE of EG@MO-2-F

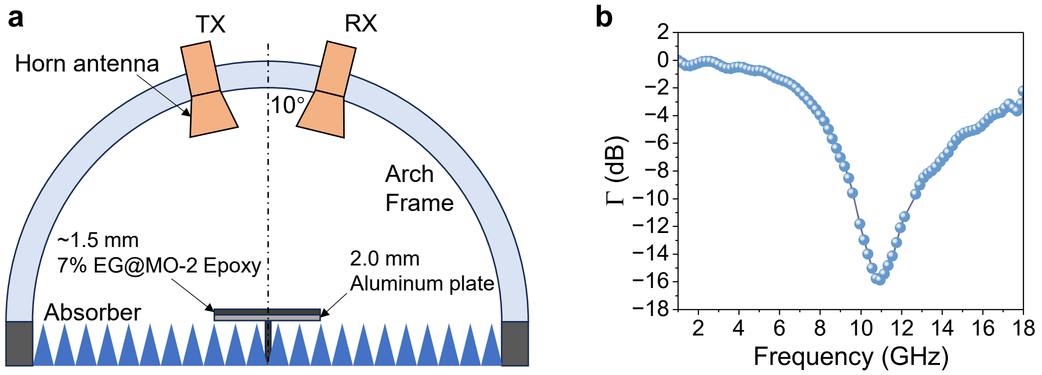

Figure S40. (a) schematic illustration of arch method testing system; (b) reflectivity Γ of EG@MO-2 measured with arch method at θ = 10°.

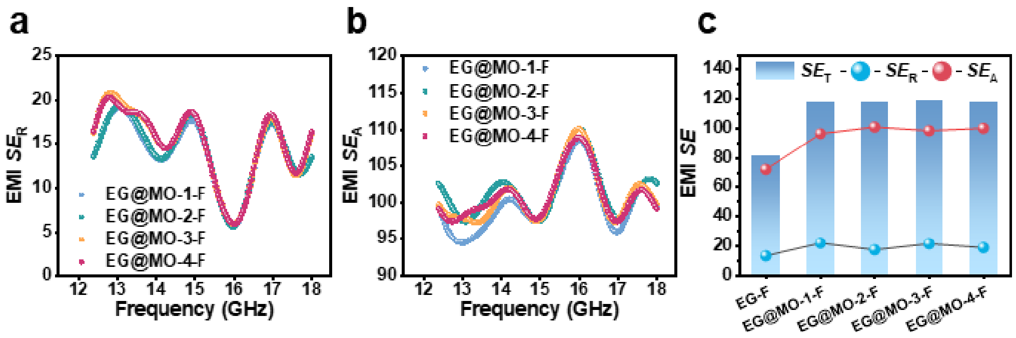

Figure S41 (a) EMI SE$_R$, (b) SE$_A$ and (c) SE$_T$ of EG@MOs film at thickness of 0.1 mm

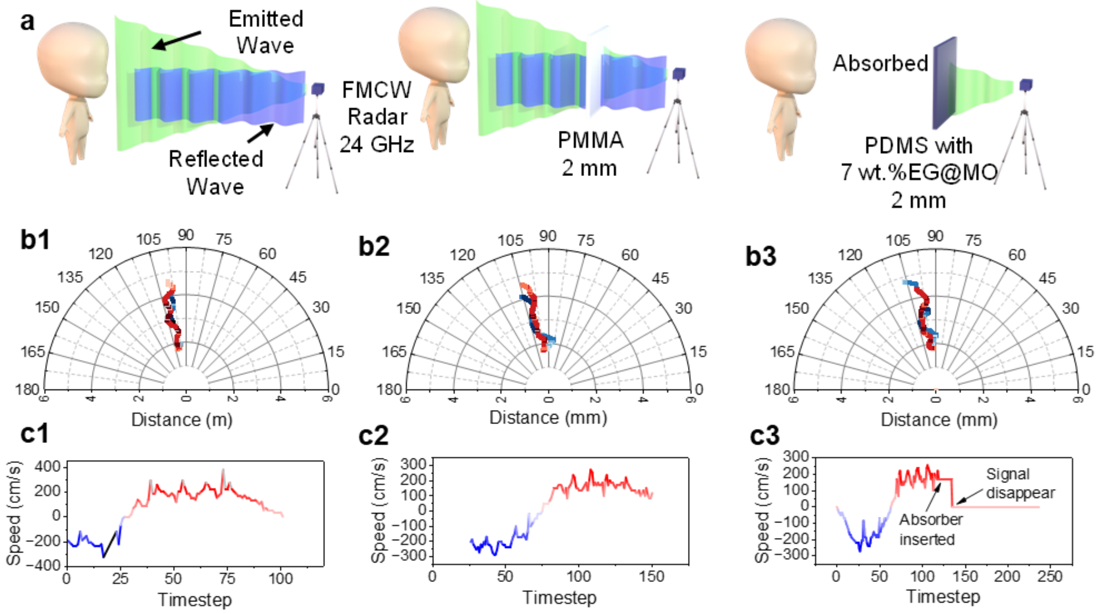

Figure S42 (a) schematic diagram of the radar test; (b1 to b3) trace and (c1 to c3) velocity of the target blocked with nothing, PMMA and PDMS with 7 wt. % of EG@MO-2.

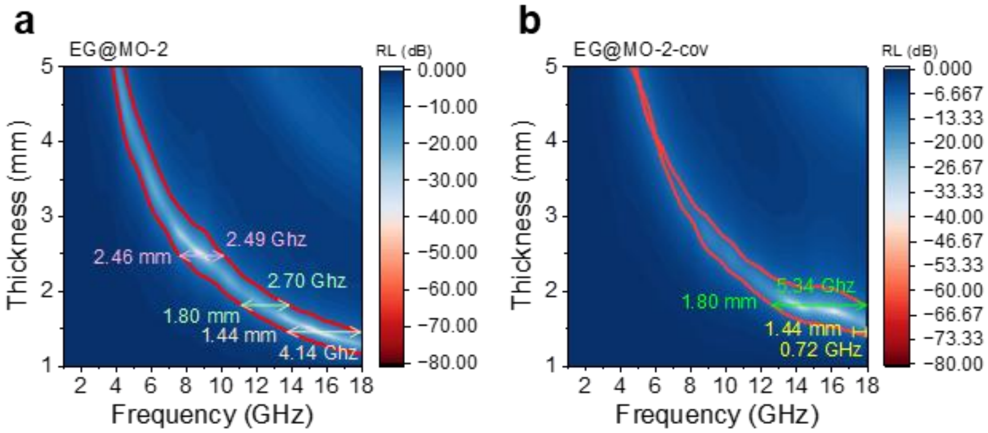

Figure S43 EAB of (a) EG@MO-2 and (b) EG@MO-2-cov at various thickness.

References

[1] Z. Tang, L. Xu, C. Xie, L. Guo, L. Zhang, S. Guo, J. Peng, Synthesis of CuCo2S4@Expanded Graphite with crystal/amorphous heterointerface and defects for electromagnetic wave absorption, Nat. Commun. 14 (2023) 1–11. https://doi.org/10.1038/s41467-023-41697-6.

[2] X. Xie, Y. Pang, H. Kikuchi, T. Liu, The synergistic effects of carbon coating and micropore structure on the microwave absorption properties of Co/CoO nanoparticles, Phys. Chem. Chem. Phys. 18 (2016) 30507–30514. https://doi.org/10.1039/C6CP05099A.

[3] H. Li, S. Bao, Y. Li, Y. Huang, J. Chen, H. Zhao, Z. Jiang, Q. Kuang, Z. Xie, Optimizing the Electromagnetic Wave Absorption Performances of Designed Co_3Fe_7@C Yolk–Shell Structures, ACS Appl. Mater. Interfaces 10 (2018) 28839–28849. https://doi.org/10.1021/acsami.8b08040.

[4] J. Xiong, Z. Xiang, B. Deng, M. Wu, L. Yu, Z. Liu, E. Cui, F. Pan, R. Liu, W. Lu, Engineering compositions and hierarchical yolk-shell structures of NiCo/GC/NPC nanocomposites with excellent electromagnetic wave absorption properties, Applied Surface Science 513 (2020) 145778. https://doi.org/10.1016/j.apsusc.2020.145778.

[5] J. Yan, Y. Huang, C. Chen, X. Liu, H. Liu, The 3D CoNi alloy particles embedded in N-doped porous carbon foams for high-performance microwave absorbers, Carbon 152 (2019) 545–555. https://doi.org/10.1016/j.carbon.2019.06.064.

[6] X. Li, D. Du, C. Wang, H. Wang, Z. Xu, *In situ* synthesis of hierarchical rose-like porous Fe@C with enhanced electromagnetic wave absorption, J. Mater. Chem. C 6 (2019) 558–567. https://doi.org/10.1039/C7TC04897A.

[7] D. Li, H. Liao, H. Kikuchi, T. Liu, Microporous Co@C Nanoparticles Prepared by Dealloying CoAl@C Precursors: Achieving Strong Wideband Microwave Absorption via Controlling Carbon Shell Thickness, ACS Appl. Mater. Interfaces 9 (2017) 44704–44714. https://doi.org/10.1021/acsami.7b13538.

[8] T. Huang, Z. Wu, J. Lin, Q. Yu, D. Tan, L. Li, A Facile Freeze-Drying Strategy To Prepare Hierarchically Porous Co/C Foams with Excellent Microwave Absorption Performance, ACS Appl. Electron. Mater. 1 (2019) 2541–2550. https://doi.org/10.1021/acsaelm.9b00565.

[9] Z. Fang, C. Li, J. Sun, H. Zhang, J. Zhang, The electromagnetic characteristics of carbon foams, Carbon 45 (2007) 2873–2879. https://doi.org/10.1016/j.carbon.2007.10.013.

[10] X. Zhao, Z. Zhang, L. Wang, K. Xi, Q. Cao, D. Wang, Y. Yang, Y. Du, Excellent microwave absorption property of Graphene-coated Fe nanocomposites, Sci Rep 3 (2007). https://doi.org/10.1038/srep03421.

[11] H.-B. Zhao, Z.-B. Fu, H.-B. Chen, M.-L. Zhong, C.-Y. Wang, Excellent Electromagnetic Absorption Capability of Ni/Carbon Based Conductive and Magnetic Foams Synthesized via a Green One Pot Route, ACS Appl. Mater. Interfaces 8 (2016) 1468–1477. https://doi.org/10.1021/acsami.5b10805.

[12] H.-B. Zhao, J.-B. Cheng, J.-Y. Zhu, Y.-Z. Wang, Ultralight CoNi/rGO aerogels

toward excellent microwave absorption at ultrathin thickness, J. Mater. Chem. C 7 (n.d.) 441–448. https://doi.org/10.1039/C8TC05239E.
[13] Y. Yin, X. Liu, X. Wei, R. Yu, J. Shui, Porous CNTs/Co Composite Derived from Zeolitic Imidazolate Framework: A Lightweight, Ultrathin, and Highly Efficient Electromagnetic Wave Absorber, ACS Appl. Mater. Interfaces 8 (2016) 34686–34698. https://doi.org/10.1021/acsami.6b12178.
[14] Y.-P. Zhang, C.-G. Zhou, W.-J. Sun, T. Wang, L.-C. Jia, D.-X. Yan, Z.-M. Li, C. Wang, T. Xu, C.-A. Wang, Microwave absorption properties of C/(C@CoFe) hierarchical core–shell spheres synthesized by using colloidal carbon spheres as templates, Ceramics International 42 (2016) 9178–9182. https://doi.org/10.1016/j.ceramint.2016.03.012.
[15] A. Iqbal, P. Sambyal, J. Kwon, M. Han, J. Hong, S.J. Kim, M.-K. Kim, Y. Gogotsi, C.M. Koo, H. Wang, K. Zheng, X. Zhang, T. Du, C. Xiao, X. Ding, C. Bao, L. Chen, X. Tian, B. Wang, C. Zhang, C. Mu, R. Yang, J. Xiang, J. Song, F. Wen, Z. Liu, Enhanced electromagnetic wave absorption properties of NiCo2 nanoparticles interspersed with carbon nanotubes, Journal of Magnetism and Magnetic Materials 471 (2019) 185–191. https://doi.org/10.1016/j.jmmm.2018.09.090.
[16] J. Tang, N. Liang, L. Wang, J. Li, G. Tian, D. Zhang, S. Feng, H. Yue, Three-dimensional nitrogen-doped reduced graphene oxide aerogel decorated with Ni nanoparticles with tunable and unique microwave absorption, Carbon 152 (2019) 575–586. https://doi.org/10.1016/j.carbon.2019.06.049.
[17] R. Shu, W. Li, Y. Wu, J. Zhang, G. Zhang, Nitrogen-doped Co-C/MWCNTs nanocomposites derived from bimetallic metal–organic frameworks for electromagnetic wave absorption in the X-band, Chemical Engineering Journal 362 (2019) 513–524. https://doi.org/10.1016/j.cej.2019.01.090.
[18] M. Ning, J. Li, B. Kuang, C. Wang, D. Su, Y. Zhao, H. Jin, M. Cao, One-step fabrication of N-doped CNTs encapsulating M nanoparticles (M = Fe, Co, Ni) for efficient microwave absorption, Applied Surface Science 447 (2018) 244–253. https://doi.org/10.1016/j.apsusc.2018.03.242.
[19] H. Liu, Y. Li, M. Yuan, G. Sun, H. Li, S. Ma, Q. Liao, Y. Zhang, In Situ Preparation of Cobalt Nanoparticles Decorated in N-Doped Carbon Nanofibers as Excellent Electromagnetic Wave Absorbers, ACS Appl. Mater. Interfaces 10 (2018) 22591–22601. https://doi.org/10.1021/acsami.8b05211.
[20] J. Zeng, X. Ji, Y. Ma, Z. Zhang, S. Wang, Z. Ren, C. Zhi, J. Yu, J. Li, L. Wang, D. Zhang, Y. Qu, G. Wang, G. Tian, A. Liu, H. Yue, S. Feng, Reduced graphene oxide modified mesoporous FeNi alloy/carbon microspheres for enhanced broadband electromagnetic wave absorbers, Mater. Chem. Front. 1 (2018) 1786–1794. https://doi.org/10.1039/C7QM00067G.
[21] B. Huang, J. Yue, Y. Wei, X. Huang, X. Tang, Z. Du, Enhanced microwave absorption properties of carbon nanofibers functionalized by FeCo coatings, Applied Surface Science 483 (2019) 98–105. https://doi.org/10.1016/j.apsusc.2019.03.301.
[22] Z. Deng, Y. Li, H.-B. Zhang, Y. Zhang, J.-Q. Luo, L.-X. Liu, Z.-Z. Yu, Lightweight

Fe@C hollow microspheres with tunable cavity for broadband microwave absorption, Composites Part B: Engineering 177 (2019) 107346. https://doi.org/10.1016/j.compositesb.2019.107346.
[23] G. Zhao, X. Cao, Q. Zhang, H. Deng, Q. Fu, A novel interpenetrating segregated functional filler network structure for ultra-high electrical conductivity and efficient EMI shielding in CPCs containing carbon nanotubes, Materials Today Physics 21 (2021) 100483. https://doi.org/10.1016/j.mtphys.2021.100483.
[24] S. Zhang, H. Sun, T. Lan, Z. Bai, X. Liu, Facile preparation of graphene film and sandwiched flexible poly(arylene ether nitrile)/graphene composite films with high EMI shielding efficiency, Composites Part A: Applied Science and Manufacturing 154 (2022) 106777. https://doi.org/10.1016/j.compositesa.2021.106777.
[25] J. Zhang, N. Kong, S. Uzun, A. Levitt, S. Seyedin, P.A. Lynch, S. Qin, M. Han, W. Yang, J. Liu, Scalable Manufacturing of Free-Standing, Strong Ti3C2Tx MXene Films with Outstanding Conductivity, Adv. Mater. (2020) 2001093.
[26] J. Zeng, X. Ji, Y. Ma, Z. Zhang, S. Wang, Z. Ren, C. Zhi, J. Yu, 3D Graphene Fibers Grown by Thermal Chemical Vapor Deposition, Advanced Materials 30 (2018) 1705380. https://doi.org/10.1002/adma.201705380.
[27] M. Ying, R. Zhao, X. Hu, Z. Zhang, W. Liu, J. Yu, X. Liu, X. Liu, H. Rong, C. Wu, Y. Li, X. Zhang, Wrinkled Titanium Carbide (MXene) with Surface Charge Polarizations through Chemical Etching for Superior Electromagnetic Interference Shielding, Angewandte Chemie 134 (2022) e202201323. https://doi.org/10.1002/ange.202201323.
[28] Y. Yang, S. Chen, W. Li, P. Li, J. Ma, B. Li, X. Zhao, Z. Ju, H. Chang, L. Xiao, H. Xu, Y. Liu, Reduced Graphene Oxide Conformally Wrapped Silver Nanowire Networks for Flexible Transparent Heating and Electromagnetic Interference Shielding, ACS Nano 14 (2020) 8754–8765. https://doi.org/10.1021/acsnano.0c03337.
[29] X. Yang, W. He, Q. Xu, H. Wang, H. Xing, J. Feng, X. Zhu, X. Li, J. Zhang, X. Zheng, Flexible and ultrathin GO@MXene sandwich-type multilayered film toward superior electromagnetic interference shielding in a wide gigahertz range of 3.95–18.0 GHz, Journal of Alloys and Compounds 946 (2023) 169338. https://doi.org/10.1016/j.jallcom.2023.169338.
[30] C. Xiang, R. Guo, S. Lin, S. Jiang, J. Lan, C. Wang, C. Cui, H. Xiao, Y. Zhang, Lightweight and ultrathin TiO2-Ti3C2TX/graphene film with electromagnetic interference shielding, Chemical Engineering Journal 360 (2019) 1158–1166. https://doi.org/10.1016/j.cej.2018.10.174.
[31] Q. Wei, S. Pei, X. Qian, H. Liu, Z. Liu, W. Zhang, T. Zhou, Z. Zhang, X. Zhang, H. Cheng, W. Ren, Superhigh Electromagnetic Interference Shielding of Ultrathin Aligned Pristine Graphene Nanosheets Film, Advanced Materials 32 (2020) 1907411. https://doi.org/10.1002/adma.201907411.
[32] M. Tan, D. Chen, Y. Cheng, H. Sun, G. Chen, S. Dong, G. Zhao, B. Sun, S. Wu, W. Zhang, J. Han, W. Han, X. Zhang, Anisotropically Oriented Carbon Films with Dual-Function of Efficient Heat Dissipation and Excellent Electromagnetic

Interference Shielding Performances, Adv Funct Materials 32 (2022) 2202057. https://doi.org/10.1002/adfm.202202057.
[33] B. Shen, W. Zhai, W. Zheng, G. Sun, G. Zhang, J. Liu, W. Xie, J. Kuo, X. Lu, M. Buyukada, F. Evrendilek, S. Sun, Ultrathin Flexible Graphene Film: An Excellent Thermal Conducting Material with Efficient EMI Shielding, Adv Funct Materials 24 (2014) 4542–4548. https://doi.org/10.1002/adfm.201400079.
[34] J. Liu, H. Zhang, R. Sun, Y. Liu, Z. Liu, A. Zhou, Z. Yu, Hydrophobic, Flexible, and Lightweight MXene Foams for High-Performance Electromagnetic-Interference Shielding, Advanced Materials 29 (2017) 1702367. https://doi.org/10.1002/adma.201702367.
[35] T.-W. Lee, S.-E. Lee, Y.G. Jeong, Highly Effective Electromagnetic Interference Shielding Materials based on Silver Nanowire/Cellulose Papers, ACS Appl. Mater. Interfaces 8 (2016) 13123–13132. https://doi.org/10.1021/acsami.6b02218.
[36] A. Iqbal, P. Sambyal, J. Kwon, M. Han, J. Hong, S.J. Kim, M.-K. Kim, Y. Gogotsi, C.M. Koo, Enhanced absorption of electromagnetic waves in Ti3C2T MXene films with segregated polymer inclusions, Composites Science and Technology 213 (2021) 108878. https://doi.org/10.1016/j.compscitech.2021.108878.
[37] T. Zhou, C. Xu, H. Liu, Q. Wei, H. Wang, J. Zhang, T. Zhao, Z. Liu, X. Zhang, Y. Zeng, H.-M. Cheng, W. Ren, Second Time-Scale Synthesis of High-Quality Graphite Films by Quenching for Effective Electromagnetic Interference Shielding, ACS Nano 14 (2020) 3121–3128. https://doi.org/10.1021/acsnano.9b08169.
[38] G. Han, Z. Ma, B. Zhou, C. He, B. Wang, Y. Feng, J. Ma, L. Sun, C. Liu, Cellulose-based Ni-decorated graphene magnetic film for electromagnetic interference shielding, Journal of Colloid and Interface Science 583 (2021) 571–578. https://doi.org/10.1016/j.jcis.2020.09.072.
[39] H. Duan, Y. Xu, D.-X. Yan, Y. Yang, G. Zhao, Y. Liu, Ultrahigh molecular weight polyethylene composites with segregated nickel conductive network for highly efficient electromagnetic interference shielding, Materials Letters 209 (2017) 353–356. https://doi.org/10.1016/j.matlet.2017.08.053.
[40] N. Chikyu, T. Nakano, G. Kletetschka, Y. Inoue, Excellent electromagnetic interference shielding characteristics of a unidirectionally oriented thin multiwalled carbon nanotube/polyethylene film, Materials & Design 195 (2020) 108918. https://doi.org/10.1016/j.matdes.2020.108918.
[41] Y. Chen, L. Pang, Y. Li, H. Luo, G. Duan, C. Mei, W. Xu, W. Zhou, K. Liu, S. Jiang, Ultra-thin and highly flexible cellulose nanofiber/silver nanowire conductive paper for effective electromagnetic interference shielding, Composites Part A: Applied Science and Manufacturing 135 (2020) 105960. https://doi.org/10.1016/j.compositesa.2020.105960.
[42] H. Chen, Y. Wen, Y. Qi, Q. Zhao, L. Qu, C. Li, Pristine Titanium Carbide MXene Films with Environmentally Stable Conductivity and Superior Mechanical Strength, Adv Funct Materials 30 (2020) 1906996. https://doi.org/10.1002/adfm.201906996.
[43] P.J. Bora, A.G. Anil, K.J. Vinoy, P.C. Ramamurthy, Outstanding Absolute

Electromagnetic Interference Shielding Effectiveness of Cross-Linked PEDOT:PSS Film, Adv Materials Inter 6 (2019) 1901353. https://doi.org/10.1002/admi.201901353.
[44] M.H. Al-Saleh, G.A. Gelves, U. Sundararaj, Copper nanowire/polystyrene nanocomposites: Lower percolation threshold and higher EMI shielding, Composites Part A: Applied Science and Manufacturing 42 (2011) 92–97. https://doi.org/10.1016/j.compositesa.2010.10.003.
[45] B. Zhou, Q. Li, P. Xu, Y. Feng, J. Ma, C. Liu, C. Shen, An asymmetric sandwich structural cellulose-based film with self-supported MXene and AgNW layers for flexible electromagnetic interference shielding and thermal management, Nanoscale 13 (2021) 2378–2388. https://doi.org/10.1039/D0NR07840A.
[46] L. Zhang, N.T. Alvarez, M. Zhang, M. Haase, R. Malik, D. Mast, V. Shanov, Preparation and characterization of graphene paper for electromagnetic interference shielding, Carbon 82 (2015) 353–359. https://doi.org/10.1016/j.carbon.2014.10.080.
[47] H. Zhang, X. Sun, Z. Heng, Y. Chen, H. Zou, M. Liang, Robust and Flexible Cellulose Nanofiber/Multiwalled Carbon Nanotube Film for High-Performance Electromagnetic Interference Shielding, Ind. Eng. Chem. Res. 57 (2018) 17152–17160. https://doi.org/10.1021/acs.iecr.8b04573.
[48] Z. Zeng, M. Chen, H. Jin, W. Li, X. Xue, L. Zhou, Y. Pei, H. Zhang, Z. Zhang, Thin and flexible multi-walled carbon nanotube/waterborne polyurethane composites with high-performance electromagnetic interference shielding, Carbon 96 (2016) 768–777. https://doi.org/10.1016/j.carbon.2015.10.004.
[49] I. Yu, J. Ko, T.-W. Kim, D.S. Lee, N.D. Kim, S. Bae, S.-K. Lee, J. Choi, S.S. Lee, Y. Joo, Effect of sorted, homogeneous electronic grade single-walled carbon nanotube on the electromagnetic shielding effectiveness, Carbon 167 (2020) 523–529. https://doi.org/10.1016/j.carbon.2020.06.047.
[50] F. Xie, F. Jia, L. Zhuo, Z. Lu, L. Si, J. Huang, M. Zhang, Q. Ma, Ultrathin MXene/aramid nanofiber composite paper with excellent mechanical properties for efficient electromagnetic interference shielding, Nanoscale 11 (2019) 23382–23391. https://doi.org/10.1039/C9NR07331K.
[51] Y. Wan, P. Xiong, J. Liu, F. Feng, X. Xun, F.M. Gama, Q. Zhang, F. Yao, Z. Yang, H. Luo, Y. Xu, Ultrathin, Strong, and Highly Flexible $Ti_3C_2T_x$ MXene/Bacterial Cellulose Composite Films for High-Performance Electromagnetic Interference Shielding, ACS Nano 15 (2021) 8439–8449. https://doi.org/10.1021/acsnano.0c10666.
[52] S. Wan, X. Li, Y. Chen, N. Liu, Y. Du, S. Dou, L. Jiang, Q. Cheng, High-strength scalable MXene films through bridging-induced densification, Science 374 (2021) 96–99. https://doi.org/10.1126/science.abg2026.
[53] W.-L. Song, J. Wang, L.-Z. Fan, Y. Li, C.-Y. Wang, M.-S. Cao, Interfacial Engineering of Carbon Nanofiber–Graphene–Carbon Nanofiber Heterojunctions in Flexible Lightweight Electromagnetic Shielding Networks, ACS Appl. Mater. Interfaces 6 (2014) 10516–10523. https://doi.org/10.1021/am502103u.
[54] F. Shahzad, M. Alhabeb, C.B. Hatter, B. Anasori, S. Man Hong, C.M. Koo, Y.

Gogotsi, Electromagnetic interference shielding with 2D transition metal carbides (MXenes), Science 353 (2016) 1137–1140. https://doi.org/10.1126/science.aag2421.
[55] Y. Liu, J. Zeng, D. Han, K. Wu, B. Yu, S. Chai, F. Chen, Q. Fu, Graphene enhanced flexible expanded graphite film with high electric, thermal conductivities and EMI shielding at low content, Carbon 133 (2018) 435–445. https://doi.org/10.1016/j.carbon.2018.03.047.
[56] R. Liu, M. Miao, Y. Li, J. Zhang, S. Cao, X. Feng, Ultrathin Biomimetic Polymeric $Ti_3C_2T_x$ MXene Composite Films for Electromagnetic Interference Shielding, ACS Appl. Mater. Interfaces 10 (2018) 44787–44795. https://doi.org/10.1021/acsami.8b18347.
[57] J. Lipton, J.A. Röhr, V. Dang, A. Goad, K. Maleski, F. Lavini, M. Han, E.H.R. Tsai, G.-M. Weng, J. Kong, E. Riedo, Y. Gogotsi, A.D. Taylor, Scalable, Highly Conductive, and Micropatternable MXene Films for Enhanced Electromagnetic Interference Shielding, Matter 3 (2020) 546–557. https://doi.org/10.1016/j.matt.2020.05.023.
[58] X. Li, X. Yin, S. Liang, M. Li, L. Cheng, L. Zhang, 2D carbide MXene Ti2CTX as a novel high-performance electromagnetic interference shielding material, Carbon 146 (2019) 210–217. https://doi.org/10.1016/j.carbon.2019.02.003.
[59] J. Li, H. Liu, J. Guo, Z. Hu, Z. Wang, B. Wang, L. Liu, Y. Huang, Z. Guo, Flexible, conductive, porous, fibrillar polymer–gold nanocomposites with enhanced electromagnetic interference shielding and mechanical properties, J. Mater. Chem. C 5 (2017) 1095–1105. https://doi.org/10.1039/C6TC04780G.
[60] C. Lei, Y. Zhang, D. Liu, K. Wu, Q. Fu, Metal-Level Robust, Folding Endurance, and Highly Temperature-Stable MXene-Based Film with Engineered Aramid Nanofiber for Extreme-Condition Electromagnetic Interference Shielding Applications, ACS Appl. Mater. Interfaces 12 (2020) 26485–26495. https://doi.org/10.1021/acsami.0c07387.
[61] A. Iqbal, J. Kwon, M.-K. Kim, C.M. Koo, MXenes for electromagnetic interference shielding: Experimental and theoretical perspectives, Materials Today Advances 9 (2021) 100124. https://doi.org/10.1016/j.mtadv.2020.100124.
[62] M. Han, C.E. Shuck, R. Rakhmanov, D. Parchment, B. Anasori, C.M. Koo, G. Friedman, Y. Gogotsi, Beyond $Ti_3C_2T_x$: MXenes for Electromagnetic Interference Shielding, ACS Nano 14 (2020) 5008–5016. https://doi.org/10.1021/acsnano.0c01312.
[63] A. Ghaffarkhah, M. Kamkar, H. Riazi, E. Hosseini, Z.A. Dijvejin, K. Golovin, M. Soroush, M. Arjmand, Scalable manufacturing of flexible and highly conductive $Ti_3C_2T_x$/PEDOT:PSS thin films for electromagnetic interference shielding, New J. Chem. 45 (2021) 20787–20799. https://doi.org/10.1039/D1NJ04513J.
[64] H. Cheng, Y. Pan, Q. Chen, R. Che, G. Zheng, C. Liu, C. Shen, X. Liu, Ultrathin flexible poly(vinylidene fluoride)/MXene/silver nanowire film with outstanding specific EMI shielding and high heat dissipation, Adv Compos Hybrid Mater 4 (2021) 505–513. https://doi.org/10.1007/s42114-021-00224-1.
[65] Z. Chen, D. Yi, B. Shen, L. Zhang, X. Ma, Y. Pang, L. Liu, X. Wei, W. Zheng,

Semi-transparent biomass-derived macroscopic carbon grids for efficient and tunable electromagnetic shielding, Carbon 139 (2018) 271–278. https://doi.org/10.1016/j.carbon.2018.06.070.
[66] A. Chaudhary, S. Kumari, R. Kumar, S. Teotia, B.P. Singh, A.P. Singh, S.K. Dhawan, S.R. Dhakate, Lightweight and Easily Foldable MCMB-MWCNTs Composite Paper with Exceptional Electromagnetic Interference Shielding, ACS Appl. Mater. Interfaces 8 (2016) 10600–10608. https://doi.org/10.1021/acsami.5b12334.
[67] C. Chang, J. Yang, G. Zhang, S. Long, X. Wang, J. Yang, Fabrication of segregated poly(arylene sulfide sulfone)/graphene nanoplate composites reinforced by polymer fibers for electromagnetic interference shielding, Nano Materials Science 4 (2022) 285–293. https://doi.org/10.1016/j.nanoms.2021.11.001.
[68] W.-T. Cao, F.-F. Chen, Y.-J. Zhu, Y.-G. Zhang, Y.-Y. Jiang, M.-G. Ma, F. Chen, Binary Strengthening and Toughening of MXene/Cellulose Nanofiber Composite Paper with Nacre-Inspired Structure and Superior Electromagnetic Interference Shielding Properties, ACS Nano 12 (2018) 4583–4593. https://doi.org/10.1021/acsnano.8b00997.
[69] W. Cao, C. Ma, S. Tan, M. Ma, P. Wan, F. Chen, Ultrathin and Flexible CNTs/MXene/Cellulose Nanofibrils Composite Paper for Electromagnetic Interference Shielding, Nano-Micro Lett. 11 (2019) 1–17. https://doi.org/10.1007/s40820-019-0304-y.
[70] L. Zou, S. Zhang, X. Li, C. Lan, Y. Qiu, Y. Ma, Step-by-step strategy for constructing multilayer structured coatings toward high-efficiency electromagnetic interference shielding, Adv. Mater. Interfaces 3 (2016) 1500476.
[71] M. Zhu, X. Yan, H. Xu, Y. Xu, L. Kong, Highly conductive and flexible bilayered MXene/cellulose paper sheet for efficient electromagnetic interference shielding applications, Ceramics International 47 (2021) 17234–17244. https://doi.org/10.1016/j.ceramint.2021.03.034.
[72] F. Zhang, P. Ren, Z. Guo, J. Wang, Z. Chen, Z. Zong, J. Hu, Y. Jin, F. Ren, Asymmetric multilayered MXene-AgNWs/cellulose nanofiber composite films with antibacterial properties for high-efficiency electromagnetic interference shielding, Journal of Materials Science & Technology 129 (2022) 181–189. https://doi.org/10.1016/j.jmst.2022.04.039.
[73] G. Yin, Y. Wang, W. Wang, D. Yu, Multilayer structured PANI/MXene/CF fabric for electromagnetic interference shielding constructed by layer-by-layer strategy, Colloids and Surfaces A: Physicochemical and Engineering Aspects 601 (2020) 125047. https://doi.org/10.1016/j.colsurfa.2020.125047.
[74] Y. Yao, S. Jin, D. Wang, J. Wang, D. Li, X. Lv, Q. Shu, Flexible magnetoelectric coupling nanocomposite films with multilayer network structure for dual-band EMI shielding, Composites Science and Technology 222 (2022) 109387. https://doi.org/10.1016/j.compscitech.2022.109387.
[75] M. Yang, Q. Wei, J. Li, Y. Wang, H. Guo, L. Gao, L. Huang, X. He, Y. Li, Y. Yuan, Flexible Composite Carbon Films Prepared by a Pancake-Making Method for Electromagnetic Interference Shielding, Adv Materials Inter 7 (2020) 1901815.

https://doi.org/10.1002/admi.201901815.
[76] Y.-Y. Wang, W.-J. Sun, D.-X. Yan, K. Dai, Z.-M. Li, Ultralight carbon nanotube/graphene/polyimide foam with heterogeneous interfaces for efficient electromagnetic interference shielding and electromagnetic wave absorption, Carbon 176 (2021) 118–125.
[77] Y. Wang, W. Wang, X. Ding, D. Yu, Multilayer-structured Ni-Co-Fe-P/polyaniline/polyimide composite fabric for robust electromagnetic shielding with low reflection characteristic, Chemical Engineering Journal 380 (2020) 122553. https://doi.org/10.1016/j.cej.2019.122553.
[78] S. Wang, D. Li, L. Jiang, D. Fang, Flexible and mechanically strong MXene/FeCo@C decorated carbon cloth: A multifunctional electromagnetic interference shielding material, Composites Science and Technology 221 (2022) 109337. https://doi.org/10.1016/j.compscitech.2022.109337.
[79] S. Wang, D. Li, L. Jiang, Synergistic Effects between MXenes and Ni Chains in Flexible and Ultrathin Electromagnetic Interference Shielding Films, Adv Materials Inter 6 (2019) 1900961. https://doi.org/10.1002/admi.201900961.
[80] Z. Tan, H. Zhao, F. Sun, L. Ran, L. Yi, L. Zhao, J. Wu, Fabrication of Chitosan/MXene multilayered film based on layer-by-layer assembly: Toward enhanced electromagnetic interference shielding and thermal management capacity, Composites Part A: Applied Science and Manufacturing 155 (2022) 106809. https://doi.org/10.1016/j.compositesa.2022.106809.
[81] Q. Song, F. Ye, X. Yin, W. Li, H. Li, Y. Liu, K. Li, K. Xie, X. Li, Q. Fu, L. Cheng, L. Zhang, B. Wei, Carbon Nanotube–Multilayered Graphene Edge Plane Core–Shell Hybrid Foams for Ultrahigh-Performance Electromagnetic-Interference Shielding, Advanced Materials 29 (2017) 1701583. https://doi.org/10.1002/adma.201701583.
[82] K. Qian, Q. Zhou, H. Wu, J. Fang, M. Miao, Y. Yang, S. Cao, L. Shi, X. Feng, Carbonized cellulose microsphere@void@MXene composite films with egg-box structure for electromagnetic interference shielding, Composites Part A: Applied Science and Manufacturing 141 (2021) 106229. https://doi.org/10.1016/j.compositesa.2020.106229.
[83] Y. Mao, D. Wang, S. Fu, Layer-by-layer self-assembled nanocoatings of Mxene and P, N-co-doped cellulose nanocrystals onto cotton fabrics for significantly reducing fire hazards and shielding electromagnetic interference, Composites Part A: Applied Science and Manufacturing 153 (2022) 106751. https://doi.org/10.1016/j.compositesa.2021.106751.
[84] Q. Liu, Y. Zhang, Y. Liu, C. Li, Z. Liu, B. Zhang, Q. Zhang, Magnetic field-induced strategy for synergistic CI/Ti3C2T /PVDF multilayer structured composite films with excellent electromagnetic interference shielding performance, Journal of Materials Science & Technology 110 (2022) 246–259. https://doi.org/10.1016/j.jmst.2021.06.084.
[85] H. Liu, R. Fu, X. Su, B. Wu, H. Wang, Y. Xu, X. Liu, Electrical insulating MXene/PDMS/BN composite with enhanced thermal conductivity for electromagnetic shielding application, Composites Communications 23 (2021)

100593.
[86] L. Liang, P. Xu, Y. Wang, Y. Shang, J. Ma, F. Su, Y. Feng, C. He, Y. Wang, C. Liu, Flexible polyvinylidene fluoride film with alternating oriented graphene/Ni nanochains for electromagnetic interference shielding and thermal management, Chemical Engineering Journal 395 (2020) 125209. https://doi.org/10.1016/j.cej.2020.125209.
[87] Z. Li, Z. Lin, M. Han, Y. Mu, P. Yu, Y. Zhang, J. Yu, Flexible electrospun carbon nanofibers/silicone composite films for electromagnetic interference shielding, electrothermal and photothermal applications, Chemical Engineering Journal 420 (2021) 129826. https://doi.org/10.1016/j.cej.2021.129826.
[88] Y. Li, B. Xue, S. Yang, Z. Cheng, L. Xie, Q. Zheng, Flexible multilayered films consisting of alternating nanofibrillated cellulose/Fe3O4 and carbon nanotube/polyethylene oxide layers for electromagnetic interference shielding, Chemical Engineering Journal 410 (2021) 128356. https://doi.org/10.1016/j.cej.2020.128356.
[89] S. Li, K. Qian, S. Thaiboonrod, H. Wu, S. Cao, M. Miao, L. Shi, X. Feng, Flexible multilayered aramid nanofiber/silver nanowire films with outstanding thermal durability for electromagnetic interference shielding, Composites Part A: Applied Science and Manufacturing 151 (2021) 106643. https://doi.org/10.1016/j.compositesa.2021.106643.
[90] M. Li, M. Zhang, Y. Zhao, S. Jiang, Q. Xu, F. Han, J. Zhu, L. Liu, A. Ge, Multilayer structured CNF/rGO aerogels and rGO film composites for efficient electromagnetic interference shielding, Carbohydrate Polymers 286 (2022) 119306. https://doi.org/10.1016/j.carbpol.2022.119306.
[91] J. Joseph, A. Sharma, B. Sahoo, J. Paul, A.M. Sidpara, PVA/ MLG/ MWCNT hybrid composites for X band EMI shielding – Study of mechanical, electrical, thermal and tribological properties, Materials Today Communications 23 (2020) 100941. https://doi.org/10.1016/j.mtcomm.2020.100941.
[92] J. Joseph, A.K. Koroth, D.A. John, A.M. Sidpara, J. Paul, Highly filled multilayer thermoplastic/graphene conducting composite structures with high strength and thermal stability for electromagnetic interference shielding applications, J of Applied Polymer Sci 136 (2019) 47792. https://doi.org/10.1002/app.47792.
[93] X. Jin, J. Wang, L. Dai, X. Liu, L. Li, Y. Yang, Y. Cao, W. Wang, H. Wu, S. Guo, Z. Chen, C. Xu, C. Ma, W. Ren, H. Cheng, H.-Y. Wu, L.-C. Jia, D.-X. Yan, J. Gao, X.-P. Zhang, P.-G. Ren, Z.-M. Li, Flame-retardant poly(vinyl alcohol)/MXene multilayered films with outstanding electromagnetic interference shielding and thermal conductive performances, Chemical Engineering Journal 380 (2020) 122475. https://doi.org/10.1016/j.cej.2019.122475.
[94] J. Gu, S. Hu, H. Ji, H. Feng, W. Zhao, J. Wei, M. Li, Multi-layer silver nanowire/polyethylene terephthalate mesh structure for highly efficient transparent electromagnetic interference shielding, Nanotechnology 31 (2020) 185303. https://doi.org/10.1088/1361-6528/ab6d9d.
[95] H. Jia, X. Yang, Q.-Q. Kong, L.-J. Xie, Q.-G. Guo, G. Song, L.-L. Liang, J.-P. Chen, Y. Li, C.-M. Chen, Free-standing, anti-corrosion, super flexible graphene

oxide/silver nanowire thin films for ultra-wideband electromagnetic interference shielding, J. Mater. Chem. A 9 (2021) 1180–1191. https://doi.org/10.1039/D0TA09246K.

[96] X. Ji, D. Chen, J. Shen, S. Guo, Flexible and flame-retarding thermoplastic polyurethane-based electromagnetic interference shielding composites, Chemical Engineering Journal 370 (2019) 1341–1349. https://doi.org/10.1016/j.cej.2019.03.293.

[97] G. Hu, C. Wu, Q. Wang, F. Dong, Y. Xiong, Ultrathin nanocomposite films with asymmetric gradient alternating multilayer structures exhibit superhigh electromagnetic interference shielding performances and robust mechanical properties, Chemical Engineering Journal 447 (2022) 137537. https://doi.org/10.1016/j.cej.2022.137537.

[98] L. He, Y. Shi, Q. Wang, D. Chen, J. Shen, S. Guo, Strategy for constructing electromagnetic interference shielding and flame retarding synergistic network in poly (butylene succinate) and thermoplastic polyurethane multilayered composites, Composites Science and Technology 199 (2020) 108324. https://doi.org/10.1016/j.compscitech.2020.108324.

[99] Z. Guo, P. Ren, B. Fu, F. Ren, Y. Jin, Z. Sun, Multi-layered graphene-Fe3O4/poly (vinylidene fluoride) hybrid composite films for high-efficient electromagnetic shielding, Polymer Testing 89 (2020) 106652. https://doi.org/10.1016/j.polymertesting.2020.106652.

[100] Q. Chen, K. Zhang, L. Huang, Y. Li, Y. Yuan, Reduced Graphene Oxide/MXene Composite Foam with Multilayer Structure for Electromagnetic Interference Shielding and Heat Insulation Applications, Adv Eng Mater 24 (2022) 2200098. https://doi.org/10.1002/adem.202200098.

[101] J. Zhou, J. Yu, D. Bai, H. Liu, L. Li, Mechanically Robust Flexible Multilayer Aramid Nanofibers and MXene Film for High-Performance Electromagnetic Interference Shielding and Thermal Insulation, Nanomaterials 11 (2021) 3041. https://doi.org/10.3390/nano11113041.

[102] B. Zhou, Z. Zhang, Y. Li, G. Han, Y. Feng, B. Wang, D. Zhang, J. Ma, C. Liu, Flexible, Robust, and Multifunctional Electromagnetic Interference Shielding Film with Alternating Cellulose Nanofiber and MXene Layers, ACS Appl. Mater. Interfaces 12 (2020) 4895–4905. https://doi.org/10.1021/acsami.9b19768.

[103] L.-Q. Zhang, B. Yang, J. Teng, J. Lei, D.-X. Yan, G.-J. Zhong, Z.-M. Li, Tunable electromagnetic interference shielding effectiveness via multilayer assembly of regenerated cellulose as a supporting substrate and carbon nanotubes/polymer as a functional layer, J. Mater. Chem. C 5 (2017) 3130–3138. https://doi.org/10.1039/C6TC05516H.

[104] J. Zhang, J. Li, G. Tan, R. Hu, J. Wang, C. Chang, X. Wang, Thin and Flexible Fe–Si–B/Ni–Cu–P Metallic Glass Multilayer Composites for Efficient Electromagnetic Interference Shielding, ACS Appl. Mater. Interfaces 9 (2017) 42192–42199. https://doi.org/10.1021/acsami.7b12504.

[105] Y. Yuan, W. Yin, M. Yang, F. Xu, X. Zhao, J. Li, Q. Peng, X. He, S. Du, Y. Li, Lightweight, flexible and strong core-shell non-woven fabrics covered by reduced

graphene oxide for high-performance electromagnetic interference shielding, Carbon 130 (2018) 59–68. https://doi.org/10.1016/j.carbon.2017.12.122.
[106] S. Shi, Z. Peng, J. Jing, L. Yang, Y. Chen, R. Kotsilkova, E. Ivanov, Preparation of Highly Efficient Electromagnetic Interference Shielding Polylactic Acid/Graphene Nanocomposites for Fused Deposition Modeling Three-Dimensional Printing, Ind. Eng. Chem. Res. 59 (2020) 15565–15575. https://doi.org/10.1021/acs.iecr.0c02400.
[107] Q. Qi, L. Ma, B. Zhao, S. Wang, X. Liu, Y. Lei, C.B. Park, An Effective Design Strategy for the Sandwich Structure of PVDF/GNP-Ni-CNT Composites with Remarkable Electromagnetic Interference Shielding Effectiveness, ACS Appl. Mater. Interfaces 12 (2020) 36568–36577. https://doi.org/10.1021/acsami.0c10600.
[108] Z. Ma, S. Kang, J. Ma, L. Shao, Y. Zhang, C. Liu, A. Wei, X. Xiang, L. Wei, J. Gu, Ultraflexible and Mechanically Strong Double-Layered Aramid Nanofiber–$Ti_3C_2T_x$ MXene/Silver Nanowire Nanocomposite Papers for High-Performance Electromagnetic Interference Shielding, ACS Nano 14 (2020) 8368–8382. https://doi.org/10.1021/acsnano.0c02401.
[109] H.J. Im, J.Y. Oh, S. Ryu, S.H. Hong, The design and fabrication of a multilayered graded GNP/Ni/PMMA nanocomposite for enhanced EMI shielding behavior, RSC Adv. 9 (2021) 11289–11295. https://doi.org/10.1039/C9RA00573K.
[110] P. He, M.-S. Cao, Y.-Z. Cai, J.-C. Shu, W.-Q. Cao, J. Yuan, Self-assembling flexible 2D carbide MXene film with tunable integrated electron migration and group relaxation toward energy storage and green EMI shielding, Carbon 157 (2020) 80–89. https://doi.org/10.1016/j.carbon.2019.10.009.
[111]Q. Gao, Y. Pan, G. Zheng, C. Liu, C. Shen, X. Liu, Flexible multilayered MXene/thermoplastic polyurethane films with excellent electromagnetic interference shielding, thermal conductivity, and management performances, Adv Compos Hybrid Mater 4 (2021) 274–285. https://doi.org/10.1007/s42114-021-00221-4.
[112] C. Gao, Y. Shi, R. Huang, Y. Feng, Y. Chen, S. Zhu, Y. Lv, W. Shui, Z. Chen, Creating multilayer-structured polystyrene composites for enhanced fire safety and electromagnetic shielding, Composites Part B: Engineering 242 (2022) 110068. https://doi.org/10.1016/j.compositesb.2022.110068.
[113] Z. Du, K. Chen, Y. Zhang, Y. Wang, P. He, H.-Y. Mi, Y. Wang, C. Liu, C. Shen, Engineering multilayered MXene/electrospun poly(lactic acid) membrane with increscent electromagnetic interference (EMI) shielding for integrated Joule heating and energy generating, Composites Communications 26 (2021) 100770. https://doi.org/10.1016/j.coco.2021.100770.
[114] M. Amini, M. Kamkar, F. Rahmani, A. Ghaffarkhah, F. Ahmadijokani, M. Arjmand, Multilayer Structures of a $Zn_{0.5}Ni_{0.5}Fe_2O_4$-Reduced Graphene Oxide/PVDF Nanocomposite for Tunable and Highly Efficient Microwave Absorbers, ACS Appl. Electron. Mater. 3 (2021) 5514–5527. https://doi.org/10.1021/acsaelm.1c00940.
